# Income Source Confusion Using the SILC

**DOI:** 10.1093/poq/nfad025

**Published:** 2023-08-09

**Authors:** Christopher Robert Bollinger, Iva Valentinova Tasseva

**Affiliations:** Gatton Endowed Professor and University Research Professor, Department of Economics, Gatton College of Business and Economics, University of Kentucky, Lexington, KY, US; Assistant Professor in International Social and Public Policy, Department of Social Policy, London School of Economics and Political Science, London, UK

## Abstract

We use a unique panel of household survey data—the Austrian version of the European Union Statistics on Income and Living Conditions (SILC) for 2008–2011—which have been linked to individual administrative records on both state unemployment benefits and earnings. We assess the extent and structure of misreporting across similar benefits and between benefits and earnings. We document that many respondents fail to report participation in one or more of the unemployment programs. Moreover, they inflate earnings for periods when they are unemployed but receiving unemployment compensation. To demonstrate the impact of income source confusion on estimators, we estimate standard Mincer wage equations. Since unemployment is associated with lower education, the reports of unemployment benefits as earnings bias downward the returns to education. Failure to report unemployment benefits also leads to substantial sample bias when selecting on these benefits, as one might in estimating the returns to job training.

## Introduction

Surveys collect income by source asking specific questions about labor market earnings, entitlement payments, and social safety net programs. Marquis and Moore (1990), Bollinger and David (1997), Bollinger and David (2000), Lynn et al. (2012), Meyer and Mittag (2019), Celhay, Meyer, and Mittag (2021), and Meyer, Mittag, and Goerge (2022) have demonstrated or suggested that source confusion in survey respondents may impact survey responses. Angel, Heuberger, and Lamei (2018) use linked survey and administrative data on Austria to show that while there is substantial error in reporting the level and receipt of many sources, the overall estimates of poverty are far less biased. While they do not explore the detailed reasons, one possible explanation is that some incomes are reported in the wrong place. Cross-reporting may imply that individuals get the specific source wrong but total income close to correct.

The allocation of income into the correct source is important for many research questions (see Bollinger and David 1997; Lynn et al. 2012). The literature using linked survey and administrative data has studied the data error properties separately in earnings or in state benefits. Hokayem, Bollinger, and Ziliak (2015) and Bollinger et al. (2019) examine nonresponse and measurement error in US earnings, and Meyer, Mok, and Sullivan (2015) and Meyer and Mittag (2019) in state transfers. Bollinger and David (2000, 2005) document the persistence of response errors over time in food stamps. Mathiowetz and Duncan (1988) examine unemployment spells, but not the benefit amount. Kapteyn and Ypma (2007) and Jenkins and Rios-Avila (2021a) analyze measurement error in earnings with Swedish and UK data, respectively, and Lynn et al. (2012) study measurement error in UK benefit receipt. It is rare to have linked survey and administrative data and even rarer to have such data for multiple income sources.

We use data from the Austrian version of the European Union Statistics on Income and Living Conditions (SILC) for 2008–2011, linked to administrative records on both Austrian state benefits and earnings. While Angel, Heuberger, and Lamei (2018)  and Angel et al. (2019) use the same linked data, they focus on the measurement of poverty and examine the relationship between household and survey characteristics and response error, respectively. Fuchs et al. (2020) show that the take-up of social assistance is overestimated with the SILC, compared to the administrative data, due to errors in the reported incomes.

There are various reasons why individuals misreport income in household surveys. Errors may be due to genuine reporting mistakes or deliberate misreporting (Tourangeau, Rips, and Rasinski 2000). Reporting mistakes could be due to program name confusion or confusion of conceptually related benefits. Individuals with many income sources may only know the total. They may also consider some benefits as earnings if those benefits are associated with their employment. Survey design may also make it easier to report different incomes as one. Deliberate misreporting may occur if items are sensitive, for example, benefits for low-income families—then individuals may conceal receipt or report them as earnings.

Our paper has two goals. First, to assess the extent and structure of misreporting across similar benefits and between benefits and earnings. Bollinger and David (2000), Celhay, Meyer, and Mittag (2021), and others have considered program confusion, but to the best of our knowledge ours is the first paper to examine reporting benefits as earnings. The three sources of income with the highest underreporting in the SILC are Austria’s three main unemployment programs: Unemployment Insurance, Unemployment Assistance, and Assistance for Covering Living Costs. The programs are not mutually exclusive. As we document below, many respondents fail to report participation in one or more of the programs. Moreover, it appears that they inflate earnings for periods when they are receiving unemployment compensation, particularly those who fail to report receipt of the benefit entirely.

Second, we demonstrate the impact of the correlated misreporting on estimates typical in the literature. Survey data are often used for estimation of wage equations because of the rich covariates available (which are often unavailable in administrative data). We document that reports of unemployment as earnings lead to a downward bias in estimates of the returns to education. We then examine the role of job training for future earnings and employment. We document that the returns to job training can be biased, in some cases quite substantially, including sign changes. The misreporting of various unemployment benefits biases the sample, as well as leading to typical types of measurement error bias.

## Unemployment Benefits in Austria

The three main unemployment benefits in Austria, recorded in the SILC, are the Unemployment Insurance (UI, “Arbeitslosengeld”), Unemployment Assistance (UA, “Notstandshilfe”), and Assistance for Covering Living Costs (ACLC, “Beihilfe zur Deckung des Lebensunterhaltes”). All three benefits are administered by the same labor market agency and allow for limited labor market participation. While UI is insurance based and is the biggest unemployment program, UA and ACLC are smaller programs targeting low-income individuals.

*UI* is *an insurance-based benefit* and is provided for a limited time, generally 20 up to 52 weeks. Qualified individuals receive a proportion (usually at 55 percent) of previous earnings for duration, dependent on age and employment history. UI is the main unemployment program in Austria, and has the highest participation of the three.

UA is targeted at low-income groups using an income test on the unemployed person’s and their partner’s (if present) earnings. It extends benefits when low-income individuals exhaust their UI benefit, so at a given point in time one can receive either UI or UA, but not both. Rather than receiving UA automatically after UI, individuals need to apply for UA but transition is supported by the labor market agency. UA can be received for up to 12 months, but follow-up applications are possible. The benefit amount is paid out as a proportion of UI—depending on circumstances, at 92 percent or 95 percent of UI—and may be capped after 6 months of receipt.

While on UI and UA, the labor market agency may require participants to take part in job training programs. *ACLC* provides income support during those programs *for low-income individuals* only and can be received simultaneously with UI or UA. The benefit lasts for the duration of the at-least-week-long training. The amount equals the difference between a guaranteed minimum and the current entitlement to UI/UA.

There are several reasons why respondents may cross-report one benefit as another in the SILC. Similar program names may cause confusion, but the program names in German are very different from each other. However, all three benefits are paid during periods of unemployment, administered by the same labor market agency, and the amounts are linked. They are similar conceptually, which may lead to program confusion (see, e.g., Balarajan and Collins 2013). For example, UA may get reported as UI, if respondents think of UA as a less generous version of UI. But respondents may also report UI as UA if they cannot recall correctly the timing of UI receipt (a problem known as “backward telescoping”). Benefit cross-reporting may occur if respondents do not know the individual benefit amounts, but know the total they receive; or if respondents find it quicker to report everything as one benefit.

Respondents may also report benefits as earnings in the SILC. There may be stigma (Currie 2006)—UA and ACLC are targeted at low-income individuals who may misreport them as earnings. Individuals may think of benefits as earnings—UI and UA are linked to previous employment, while ACLC pays for job training. Or individuals may not know the individual income amounts but the total they receive and report only one source.

## Data: The Austrian SILC

The SILC is a household income survey conducted in all EU countries and beyond (Atkinson and Marlier 2010). The Austrian SILC used in our analysis is for survey years 2008–2011 (income reference period for the calendar years 2007–2010). The sample is based on all private households registered in the central population register (“Zentrales Melderegister”). The data include information on individual and household characteristics, including specific income sources. SILC serves as the main source for official statistics on income, poverty, and inequality, produced by Statistics Austria and Eurostat.

The key feature of the data, which allows us to provide novel evidence on income cross-reporting, is that the data include individual-level survey reports and administrative records on both earnings and a range of state benefits. Next, we describe in more detail the main data characteristics.

### Linked Survey Reports and Administrative Records

Starting in 2012, SILC survey participants are no longer asked to provide information on earnings and certain types of state benefit; this information is acquired directly from various administrative sources (Heuberger, Glaser, and Kafka 2013). To provide a continuous data series, Statistics Austria retrospectively matched the individuals in the survey to their administrative data for the survey years 2008–2011 (Heuberger, Glaser, and Kafka 2013).

Survey respondents were matched using a pseudonymized personal identifier used nationally for each administrative data source and in the central population register used for the SILC sampling frame (Heuberger, Glaser, and Kafka 2013). The SILC collects data about all household members at an address. Someone who is living but is not registered at an address—and so is not part of the SILC sampling frame—may end up in the sample. Their identifier then needs to be obtained but may not be found due to errors in the survey or administrative data (e.g., incorrect birth date) or if nonexistent (e.g., for younger individuals or individuals with nonAustrian citizenship). Identifiers were found for 95.6 percent of the sample in 2008, 97.7 percent in 2009, 96.8 percent in 2010, and 99.4 percent in 2011 (Statistics Austria 2014).^1^

The earnings data derive from wage tax data electronically supplied by employers to the tax authority. The wage tax data include data on all taxable earnings of employees and pensioners, potentially excluding any undeclared under-the-table earnings that may be captured in the survey. The benefit data are provided by the authority administering benefit payments (see Angel, Heuberger, and Lamei 2018).

Recent work by Kapteyn and Ypma (2007) and Jenkins and Rios-Avila (2021a) suggests that mismatch can play a role. The Austrian system samples from an original frame that already has the identifier, hence the match is generally not ex post. The high rates of deterministic match are evidence that this likely does not play a role. Kapteyn and Ypma (2007), Paulus (2015), Bollinger et al. (2018), Bollinger et al. (2019), and Jenkins and Rios-Avila (2021a) all suggest that administrative earnings may fail to reflect under-the-table earnings. We show that some of what is often believed to be under-the-table earnings may actually be cross-reporting benefits as earnings. We note that generally administrative records of state benefits are believed to be quite accurate (Bollinger and David 1997; Meyer and Mittag 2019). We proceed assuming the benefit administrative data represent the truth; we remain ambiguous about the earnings records.

The survey information on earnings and benefits is based on individual-level interviews with adult respondents aged 16+.^2^ We use the SILC “additional data” files with detailed information for the level of gross earnings and state benefits and the number of months in receipt for each income. These data give us the necessary level of detail to distinguish between the different types of unemployment benefit. Angel, Heuberger, and Lamei (2018)  and Angel et al. (2019) use the SILC harmonized income variables, which aggregate all unemployment benefits into a single variable.

The reference period of the survey and administrative data is the calendar year before the date of interview. We compare the gross annual amounts based on the survey versus administrative data. Both the survey and administrative data measure gross annual earnings. The administrative records measure annual benefit amounts, while the survey measures monthly amounts. We multiply the monthly benefit amount by the number of months in receipt to derive gross annual benefit amounts in the survey.^3^

### Sample

The SILC follows individuals in a four-year rotating panel. Individuals are staggered so that each year 25 percent of the sample is renewed. Individuals initially surveyed in 2008 appear in all four years of our sample. Individuals initially interviewed in either 2005 or 2011 appear only once. Table 1 exhibits the number of observations in our sample by wave.

**Table 1.** Sample characteristics: SILC 2008–2011.

	All	Men	Women
Age	47.4	46.6	48.1
Education:			
Low	23.4	17.3	29.0
Middle	59.7	61.9	57.7
High	16.9	20.8	13.4
With partner	62.5	66.1	59.2
Country of birth:			
Austria	86.0	86.8	85.4
EU-27	5.9	5.1	6.6
Other	8.1	8.1	8.0
Full-time, full-year employed	30.8	41.8	21.0
With survey earnings	54.3	59.4	49.8
Proxy interview:			
No	82.6	80.4	84.5
Partner	7.8	11.0	4.8
Someone else	9.6	8.5	10.6
Job training:			
Did not take	67.6	67.1	68.0
Mostly paid with own resources	5.1	4.7	5.4
Employer	13.8	16.3	11.5
Labor market agency	1.4	1.3	1.5
Other institutions	0.8	0.8	0.8
Missing/n/a	11.4	9.8	12.8
UI in the survey:			
Missing/imputed	0.1	0.2	0.1
No receipt	94.8	93.8	95.6
With receipt:	5.1	6.0	4.2
*1 year only*	74.2	71.9	77.0
*Multiple years*	25.8	28.1	23.0
UA in the survey:			
No receipt	98.3	98.2	98.5
With receipt:	1.7	1.8	1.5
*1 year only*	73.5	70.1	76.6
*Multiple years*	26.5	29.9	23.4
ACLC in the survey:			
Missing/imputed	0.1		
No receipt	99.2		
With receipt:	0.7		
*1 year only*	88.6		
*Multiple years*	11.4		
Observations:			
1 wave only	8,903	4,231	4,672
2 waves	12,559	5,951	6,608
3 waves	15,461	7,302	8,159
4 waves	8,047	3,790	4,257
All observations	44,970	21,274	23,696

*Source:* Own calculations with the SILC.

*Note:* The sample includes individuals aged 16+. The table shows the mean age and the share (in %) of individuals by different characteristics. Groups with missing/imputed cases in survey earnings, country of birth and UA receipt, and ACLC receipt by gender are too small to disclose.

We include individuals aged 16+, but limit earnings regressions to ages 19–64. We exclude missing/imputed values for earnings as an outcome and a control variable. Where a categorical control variable (e.g., occupation) has missing/imputed values, we use a dummy variable to indicate this.

Table 1 provides summary statistics for the full sample (column 1), men (column 2), and women (column 3), including observations with missing/imputed values. The proportion of cases with missing/imputed UI/ACLC survey is 0.1 percent, while the missing/imputed cases in survey earnings are too few to disclose.

There are 44,970 observations in total, with slightly more women (23,696) than men (21,274). Mean age is 47.4 years. The majority of people (59.7 percent) have middle-level education, though men tend to be more educated than women. Men are also more likely to be in full-time, full-year employment than women and to have earnings—59.4 percent of men versus 49.8 percent of women—suggesting a prevalence of part-time and/or part-year employment among women. The sample has 86 percent born in Austria, 5.9 percent in the rest of the EU-27, and 8.1 percent in Turkey, the Western Balkans, or other countries. In the sample, 21 percent reported job training, with 1.4 percent having training by the labor market agency. Of respondents, 5.1 percent received UI, 1.7 percent UA and 0.7 percent ACLC. More men (6 percent) report UI than women (4.2 percent), but the proportions by sex are similar for UA (ACLC receipt by sex is too small to disclose). Most UI/UA/ACLC recipients report receipt in one year only, while around a quarter of UI and UA and 11.4 percent of ACLC recipients had spells spanning multiple years.

## Results

To document the extent and relationship of misreporting in the three unemployment programs and labor market earnings, we employ a number of approaches. First, we examine cross tabulations and average benefit and earnings amounts compared to administrative amounts. Second, we estimate linear probability models of benefit receipt in the survey conditional on receipt in the administrative data, with demographic control variables included. We include administrative earnings and the difference in survey and administrative earnings as well as measures of reporting accuracy (false positive, false negative, and true positive) as main variables of interest. Finally, we examine how the levels of misreporting, especially for earnings, are correlated with the accuracy of reporting other benefits.

### Benefits and Earnings Underreporting

Table 2 documents the extent of earnings and benefit misreporting in the survey for each income source separately and for the total of the three benefits (UI+UA+ACLC). The first row reports the rates of false negatives: the share of recipients according to the administrative data who do not report the benefit in the survey. The rate is high for all three benefits: 42.6 percent for UI, 52.1 percent for UA, and 77.4 percent for ACLC. The false negative rate for respondents receiving any of the unemployment benefits is lower, at 38.4 percent. The overall receipt of any unemployment benefits is better captured in the survey than each individual benefit. The false negative rate for earnings is 8.9 percent. One explanation is that some individuals simply minimize effort on the survey by denying income sources.

**Table 2.** Misreporting of unemployment benefits and earnings.

		UI	UA	ACLC	Total	Earnings
False –	%	42.6	52.1	77.4	38.4	8.9
	Obs.	1,399	683	185	1,517	2,174
a) False +	%	0.8	0.2	0.6	0.6	6.2
	Obs.	317	105	263	245	1,195
b) False +	%	22.0	1.5	5.0		
	Obs.	151	44	202		
*True +*						
Mean administrative amount		2,847	4,419	2,138	3,646	29,795
Mean survey amount		3,030	4,397	2,690	3,784	28,727
Error in % of administrative amount:						
≤–50%		8.7	14.0		9.3	3.9
*>*–50% & ≤–10%		28.6	20.0		26.7	29.8
*>*–10% & *<*10%		23.4	28.1		27.0	39.0
≥10% & *<*50%		18.9	20.8		20.0	17.7
≥50%		20.3	17.0		17.0	9.7
SD of error		1,840	2,307	2,130	1,990	19,677
Correlation administrative and survey amounts		0.74	0.68	0.64	0.78	0.71
Observations		1,887	629	54	2,435	22,234
*All (true +/false +/false –)*						
Mean administrative amount		2,257	3,365	662	2,937	26,334
Mean survey amount		1,911	2,232	1,537	2,418	25,696
Error in % of administrative amount:						
≤–50%		43.4	54.4	38.0	41.6	11.9
*>*–50% & ≤–10%		15.0	8.9		15.5	25.8
*>*–10% & *<*10%		12.3	12.5		15.7	33.9
≥10% & *<*50%		9.9	9.2		11.6	15.3
≥50% (or undefined if false +)		19.5	15.0	55.0	15.7	13.1
SD of error		2,618	3,240	2,858	2,728	19,786
Correlation administrative and survey amounts		0.44	0.41	−0.02	0.58	0.71
Observations		3,603	1,417	502	4,197	25,603

*Source:* Own calculations with the SILC.

*Note:* Total = UI + UA + ACLC. Rates in percent and number of observations shown for false negative/positive. False negative in percent is the share of benefit recipients according to the administrative data who do not report the benefit in the survey. Two definitions of false positive are considered: a) in percent equals the share of benefit/earnings nonrecipients according to the administrative data who report the benefit/earnings in the survey; b) in percent equals the number of benefit nonrecipients according to the administrative data who report a receipt in the survey, as a share of the number of recipients of the other two benefits in the administrative data (by definition, there is no estimate for the total and earnings). Mean amounts and errors are based on the sample of true positives only, that is, those who receive benefits/earnings in both the administrative and survey data, or the total sample of true positives/false positives/false negatives. The error in amount equals the difference between the survey and administrative amount. Groups with different size of error in ACLC are too small to disclose. Observations with missing/imputed benefit values are excluded. Sample is restricted to those aged 16+.

We report two definitions of false positive. The first, typically found in the literature, is the share of all administrative nonrecipients of the income source who report receipt in the survey. False positives tend to be low as a rate, especially for social safety net programs, as the denominator is large. Indeed, for each unemployment benefit as well as the total, the rate is less than 1 percent, though the number of false positives is not insubstantial—105 for UA to 317 for UI. The issue of underreporting benefit receipt seems to be more serious, consistent with findings of underreporting UK and US state benefits in survey data (Lynn et al. 2012; Meyer, Mok, and Sullivan 2015; Meyer and Mittag 2019). For earnings, 6.2 percent of those with no administrative earnings report earnings. One explanation for this is under-the-table earnings. However, another explanation may be reporting of social safety net income as earnings.

The second definition of false positive measures the extent of false positives for individuals, conditional on administrative receipt of one of the other unemployment programs. These false positives are suggestive of program confusion of some type. The rate is relatively high for UI—22 percent of UA/ACLC recipients falsely report a UI receipt in the survey.

In the remaining rows, we document misreporting of amounts first for those with receipt in both survey and administrative measures, and for all who either receive or report receipt. The average differences for all four income sources are typically not large, but this masks substantial variation. For true positives, across all four sources, a large proportion are within 10 percent. This is particularly true for earnings and UA, and for the combined total of the three benefits. Only for UI are underreports of 10 percent to 50 percent more common. For all those who receive or report receipt, large false negatives for UI/UA/ACLC dominate the distributions. For earnings, differences within 10 percent dominate, although false negatives and false positives place more weight in the tails.

### Benefits and Earnings Cross-Reporting

#### Cross-reporting at the extensive margin

Accuracy of reporting at the extensive margin—correctly reporting a source of income—is important for many applications, such as estimating benefit take-up rates and understanding overall reporting behavior. Table 3 provides cross tabulations focusing on combinations of survey income receipt, given administrative receipt. Each row provides the proportion of those in one administrative category reporting benefit and income receipt in the survey. For example, the first row are individuals who, based on administrative data, only received UI income. While 38.3 percent correctly reported this, fully 11.2 percent reported only earnings. We draw attention to the fact that very few individuals receiving “UI only” in the administrative record reported receiving other benefits in the survey, while 11 percent of those who received “UA only” reported only receiving UI.

**Table 3.** Combinations of income receipt, given administrative receipt.

	Survey data									
Admin data	UI only	UA only	Earn. only	UI + earn.	UA + earn.	UI + UA	UI + UA + earn.	ACLC + others	None	*Obs.*
UI only	38.3		11.2	10.2					31.1	206
UA only	11.0	48.6	6.4						22.8	346
UA onlyEarn. only			91.0	0.3				0.1	8.5	21,042
UA onlyUI + earn.	2.7		38.0	51.1				2.4	2.9	2,490
UA onlyUA + earn.		12.0	27.3	14.3	18.7		6.7	7.7		300
UA onlyUI + UA				.		16.8		18.5	16.8	119
UA onlyUI + UA + earn.			23.4	28.8	5.4		16.6	6.8		368
UA onlyACLC + others			31.4	8.7				22.3	29.8	242
None	0.1	0.2	5.4	0.3				0.1	93.8	18,395
Observations	304	328	22,348	1,652	112	73	112	317	19,623	

*Source:* Own calculations with the SILC.

*Note:* Each row provides the proportion of those in one administrative category reporting benefit and income receipt in the survey. UI = unemployment insurance benefit; UA = unemployment assistance; ACLC = assistance for covering living costs; earn. = earnings. Missing values (.) indicate cells with too few cases (cannot be disclosed). Numbers on the diagonal are highlighted in light gray. Otherwise, numbers with a value of 10 percent or more are highlighted in dark gray. Observations with missing/imputed administrative/survey values are excluded. Sample is restricted to those aged 16+.

We highlight three important regularities in this table. First, the diagonal that represents when the survey response agrees with administrative record in receipt typically has entries at or below 50 percent. The only exceptions are the “Earnings only” and “none” rows. Second, the column “Earnings only” represents individuals who report only labor market earnings in the survey. The high percentages in this column demonstrate that many respondents simply report myriad benefit and earnings combinations as earnings only. Finally, we note that those who are only on UI, UA, or some combination of UI, UA, and ACLC (the first, second, sixth, and eighth rows) have relatively high percentages in the “none” column, reflecting complete denial of benefit receipt.

Next, we estimate linear probability models for the probability of reporting a benefit receipt in the survey, conditional on administrative receipt for UI and UA. We report a focused subset of coefficients here; full specifications and robustness checks including logit estimates are in Supplementary Material tables S1–S4. Due to disclosure restrictions, we are unable to provide estimates for ACLC. These models investigate false negative reports and allow us to control for myriad demographic characteristics, earnings and receipt and report of the other programs. These models are not meant to identify causal effects, but rather highlight associations similar to those in table 3 while allowing us to control for other factors.

Table 4 presents results for UI. In column (1), we condition on the logarithm of the administrative amount of UI; the administrative amount as well as the discrepancy between the survey and administrative amounts of earnings (in thousands); and dummies for false positive, false negative, and true positive in earnings, UA, and ACLC, with true negative being omitted. This first specification focuses on administrative characteristics of the UI program and the reporting of other incomes. In column (2), we control for a rich set of covariates, including basic demographic, household, economic, and survey structure (see notes to table 4).

**Table 4.** Probability of reporting the unemployment insurance (UI) benefit in the survey, conditional on receiving it.

	(1)		(2)	
	*β*		*β*	
	*(se)*	*ρ*	*(se)*	*ρ*
Constant	−0.384		−0.221	
	(0.071)	.000	(0.143)	.123
Woman			−0.034	
			(0.022)	.122
Ln admin UI	0.118		0.136	
	(0.008)	.000	(0.013)	.000
Admin earnings (in thousand)	−0.008		−0.008	
	(0.001)	.000	(0.001)	.000
Survey–admin earnings (in thousand)	−0.013		−0.013	
	(0.001)	.000	(0.001)	.000
Earnings: True –	ref		ref	
False +	0.161		0.156	
	(0.069)	.019	(0.070)	.026
False –	−0.048		−0.015	
	(0.045)	.287	(0.045)	.743
True +	0.270		0.257	
	(0.036)	.000	(0.037)	.000
UA: True –	ref		ref	
False +	−0.143		−0.152	
	(0.080)	.076	(0.087)	.082
False –	−0.002		−0.006	
	(0.031)	.940	(0.030)	.854
True +	0.064		−0.017	
	(0.036)	.077	(0.038)	.661
ACLC: True –	ref		ref	
False +	0.043		0.006	
	(0.048)	.366	(0.048)	.893
Admin benefit duration (in months)			−0.019	
			(0.006)	.002
Job training: Did not take			ref	
Labor market agency			0.112	
			(0.030)	.000
Mostly paid with own resources			0.071	
			(0.037)	.056
Employer			0.032	
			(0.031)	.310
Other institutions			0.041	
			(0.079)	.600
No proxy			ref	
Partner is proxy			−0.099	
			(0.033)	.002
Someone else is proxy			−0.101	
			(0.032)	.002
Controls	No		Yes	
R-squared	.137		.183	
Observations	3,155		3,155	

*Source:* Own calculations with the SILC.

*Note:* This table shows an estimation of a linear probability model. The dependent variable equals 1 if the benefit amount is positive in both the survey and administrative data; and 0 if the survey amount is 0 while the administrative amount is positive. “True +” implies positive income amounts in both the survey and administrative data; “false +” means positive amount in the survey and zero in the administrative data; “false –“ means zero in the survey and positive amount in the administrative data; and “true –” means zero amounts in both the survey and administrative data. Column (2) adds controls for: age group (in 5-year age bands), number of children in the household (0, 1, 2, 3+), number of adults in the household (1, 2, 3+), the highest achieved education level (low, middle, high), if in a couple, health status (6 categories), region (Vienna, borough with more than 100,000 residents, borough with 10,000–100,000 residents, borough with less than 10,000 residents), occupation (12 categories), industry (25 categories), being a civil servant, country of birth (7 categories), wave (interviewed for the 1st, 2nd, 3rd, 4th time), interaction between wave and year (2008 to 2011), interview type (in person or by phone), same interviewer as last year. Sample is restricted to those aged 16+. Observations with missing/imputed administrative/survey UI or earnings are excluded. Cells with too few observations cannot be disclosed and are not shown: UA with missing/imputed values; ACLC false -, true +, and with missing/imputed values; and job training with missing or n/a values. Standard errors clustered by individual and shown in parentheses. *p-*values shown next to standard errors and based on a two-tailed significance test.

The statistically significant coefficients of interest in both specifications are qualitatively and quantitatively similar. The results are robust to differences across characteristics, suggesting strong underlying associations. We find that higher administrative earnings and larger earnings overreports in the survey are negatively associated with correctly reporting receipt of UI, with coefficients of −0.008 and −0.013, respectively. One explanation is a negative correlation among errors if administrative earnings fail to capture parts of true earnings; another explanation is that respondents report UI as earnings. The combination of coefficients on true positive for earnings, and the levels, also suggest that respondents may be reporting UI twice in the survey: once as earnings and then again as UI. In the survey, people are first asked about their earnings and then about benefit entitlements. This may lead to some of the cross-reporting. Table 4 also presents results consistent with cross-reporting of UI and UA. Reporting UI in the survey declines with a false positive in UA by 0.152, compared to a true negative.

The amount of UI matters: the coefficient on log of UI administrative amount is 0.136; a 1 percent increase in UI is associated with a 0.1 percent higher probability of reporting UI receipt. Participating in job training paid by the labor market agency is also positively associated with reporting UI in the survey (a coefficient of 0.112).

Holding constant the UI amount, the longer one receives UI, the less likely one is to report its receipt. One explanation could be stigma associated with a long spell of UI because the respondent was unable to find employment. Alternatively, this could be due to recall error if the spell crossed years. We find that the later in the interview year respondents are interviewed, and thus the larger the time gap between the income reference and data collection period, the less likely they are to accurately report UI receipt in the survey (see Supplementary Material table S1).

Table 5 presents estimates for the probability of reporting UA in the survey, conditional on administrative receipt. These results suggest it is possible UA is also being misreported as earnings or other benefits. As with UI, higher administrative earnings and earnings overstatement in the survey are associated with lower probability of reporting UA. Furthermore, the probability of reporting UA in the survey is reduced with a false positive in earnings, by 0.247. These results suggest respondents may be putting UA into earnings. Results are also consistent with respondents reporting UA as UI. Reporting UA declines with a false positive in UI, relative to a true negative. The coefficient (−0.298) is bigger than the coefficient for reporting UI of a false positive in UA (−0.152 in table 4). Similarly to table 4, the common coefficients across both specifications are generally qualitatively and quantitatively similar.

**Table 5.** Probability of reporting the unemployment assistance (UA) in the survey, conditional on receiving it.

	(1)		(2)	
	*β*		*β*	
	*(se)*	*ρ*	*(se)*	*ρ*
Constant	−0.109		0.459	
	(0.093)	.242	(0.168)	.006
Woman			−0.023	
			(0.032)	.480
Ln admin UA	0.094		0.035	
	(0.011)	.000	(0.015)	.022
Admin earnings (in thousand)	−0.012		−0.010	
	(0.003)	.000	(0.003)	.004
Survey–admin earnings (in thousand)	−0.015		−0.013	
	(0.002)	.000	(0.002)	.000
Earnings: True –	ref		ref	
False +	−0.279		−0.247	
	(0.057)	.000	(0.058)	.000
False –	0.023		0.017	
	(0.042)	.584	(0.044)	.694
True +	0.011		−0.007	
	(0.042)	.794	(0.046)	.880
UI: True –	ref		ref	
False +	−0.312		−0.298	
	(0.045)	.000	(0.046)	.000
False –	−0.107		−0.052	
	(0.038)	.005	(0.039)	.176
True +	−0.058		−0.049	
	(0.036)	.106	(0.039)	.215
Missing/imputed	−0.039		−0.092	
	(0.045)	.376	(0.043)	.033
ACLC: True –	ref		ref	
False +	0.177		0.034	
	(0.042)	.000	(0.048)	.477
Admin benefit duration (in months)			0.015	
			(0.006)	.013
Job training: Did not take			ref	
Labor market agency			0.181	
			(0.037)	.000
Mostly paid with own resources			0.029	
			(0.071)	.682
Employer			−0.065	
			(0.067)	.335
No proxy			ref	
Partner is proxy			−0.078	
			(0.065)	.227
Someone else is proxy			0.027	
			(0.049)	.582
Controls	No		Yes	
R-squared	.234		.298	
Observations	1,262		1,262	

*Source and note:* See table 4. Observations with missing/imputed administrative/survey UA or earnings are excluded. Cells with too few observations cannot be disclosed and are not shown: ACLC false –, true +, and with missing/imputed values; and job training by other institutions and with n/a values.

The amount of UA matters, consistent with results for UI. However, unlike UI, controlling for demographic characteristics does markedly reduce this association. A 1 percent increase in the UA amount increases the probability of reporting UA in the survey by 0.035 percent. Job training by the labor market agency also means it is more likely to accurately report UA receipt (by 0.181). A longer benefit duration is positively associated with reporting UA. Unlike with UI, UA recipients can send follow-up applications to extend benefit duration, and hence be on UA for long spells.

Supplementary Material tables S5 and S6 repeat the above analyses by gender. Women seem more prone to confusion between UI and UA than men. Misreporting of both UI and UA is positively associated with the level of administrative earnings and the difference between survey and administrative earnings. Women appear less likely to report a benefit receipt in both earnings and the benefit. Reporting UI receipt in the survey increases with a false positive in earnings by 0.31 for men, suggesting reporting UI receipt twice, while we find a small negative coefficient for women (Supplementary Material table S5). Women may be less prone to inflate their total income than men, but more likely to report benefits in the wrong category.

#### Cross-reporting at the intensive margin

While false negatives are large and important, errors at the intensive margin—in the reported amount of an income source—are also important to understanding overall reporting behavior. Table 6 provides information on how the level of reporting (the exact Euro amount) is associated with reporting of different benefits. It compares the mean survey and administrative amounts (columns 2 and 3) separately by benefit type, earnings, and total income (all unemployment benefits + earnings) conditional on benefit receipt and report. It also presents the mean error in level and in percent of the mean administrative income (column 5), the standard deviation of the errors (column 6), and correlation between the survey and administrative amounts (column 7).

**Table 6.** Mean survey and administrative amounts by benefits true positive, false negative, and false positive.

	Survey	Admin	Δ	… in % of Admin Income	SD of Δ	*ρ*
Conditional on UI true +						
UI	2,987	2,802	185	1.09	1,804	.74
UA	166	323	−156	−0.92	756	.69
ACLC	81	4	77	0.45	571	.00
Earnings	13,660	13,809	−149	−0.88	11,268	.64
Income	16,894	16,938	−44	−0.26	11,075	.63
Observations	1,801					
Conditional on UI false –						
UI	0	1,946	−1,946	−11.00	2,068	
UA	309	342	−34	−0.19	1,247	.57
ACLC	127	3	125	0.71	1,044	−.01
Earnings	19,126	15,405	3,722	21.03	11,437	.67
Income	19,562	17,696	1,867	10.55	11,351	.65
Observations	1,340					
Conditional on UI false +						
UI	3,643	0	3,643	44.44	2,903	
UA	442	2,271	−1,828	−22.30	2,933	.30
ACLC	122	10	112	1.36	689	−.01
Earnings	8,336	5,917	2,419	29.51	11,219	.35
Income	12,543	8,198	4,345	53.00	11,051	.27
Observations	299					
Conditional on UA true +						
UI	1,115	1,034	81	0.98	1,710	.62
UA	4,500	4,558	−58	−0.70	2,295	.70
ACLC	165	3	162	1.96	678	−.01
Earnings	2,613	2,664	−50	−0.61	2,696	.84
Income	8,393	8,258	135	1.64	3,009	.79
Observations	514					
Conditional on UA false –						
UI	1,903	1,279	623	6.56	3,036	.29
UA	0	2,904	−2,904	−30.56	2,770	
ACLC	80	2	78	.82	527	−.01
Earnings	9,541	5,316	4,225	44.47	9,765	.53
Income	11,524	9,501	2,022	21.28	9,408	.53
Observations	599					
Conditional on UA false +						
UI	1,226	1,578	−351	−5.11	2,412	.53
UA	3,807	0	3,807	55.31	3,062	
ACLC	119	0	119	1.73	462	
Earnings	2,588	5,304	−2,716	−39.46	8,669	.25
Income	7,740	6,882	859	12.48	8,300	.50
Observations	94					
Conditional on ACLC false –						
UI	916	808	108	1.14	1,111	.78
UA	37	57	−20	−0.21	431	−.02
ACLC	0	635	−635	−6.74	884	
Earnings	8,996	7,918	1,079	11.45	4,233	.89
Income	9,949	9,418	531	5.64	4,391	.87
Observations	41					
Conditional on ACLC false +						
UI	2,103	2,388	−285	−2.67	3,086	.46
UA	1,416	1,787	−371	−3.47	2,132	.69
ACLC	2,522	0	2,522	23.62	3,034	
Earnings	4,966	6,501	−1,535	−14.38	17,798	.63
Income	11,007	10,675	331	3.10	17,835	.62
Observations	211					

*Source:* Own calculations with the SILC.

*Note:* Income = UI + UA + ACLC + Earnings. Δ = mean survey—mean administrative amount. SD of Δ = standard deviation of (survey amount—administrative amount). *ρ* = correlation between survey and administrative amounts. Group with ACLC true positives is too small to disclose. Sample includes those aged 16+. Observations with missing/imputed earnings or benefits (UI, UA, or ACLC) are excluded.

While on average there is a gap between the survey and administrative amounts, the errors by income source tend to partly offset each other and reduce the overall error in income. The errors in the UI/UA true positive groups, as measured by both average delta and standard deviation of delta, are the smallest, suggesting that respondents who correctly report receipt of at least one of their benefits tend to make fewer mistakes in levels.

For those who fail to report receipt of UI or UA, the error in earnings (and income) is higher both in mean value and as measured by the standard error of the difference. While the error for income in these cases is still large, it is smaller on average than either the underreporting of the benefit or the overreporting of the earnings.

Table 7 examines earnings errors (survey–administrative earnings) by combined benefits status. We take the sample of respondents overreporting earnings and receiving benefits in period *t* while not receiving benefits in period *s*. We split the benefit status in period *t* by false negatives and true positives, with an error in the reported benefit amount of *<*10 percent versus ≥10 percent. The average error in earnings is smaller in the periods when no benefits are received, particularly for respondents who fail to report any of the benefits they receive or when they make a large error in the amount they report. The difference in earnings overreports with benefit receipt suggests that some of the overreport is benefits.

**Table 7.** The mean of survey–admin earnings, conditional on overreporting earnings and receiving benefits in period *t* while not receiving benefits in period *s.*

		*Survey*–*admin earnings*	
	Obs.	Mean in *t*	Mean in *s*
*Benefits in t*			
False –	295	8,007	2,523
True + & abs error in sum of benefits			
*<* 10%	64	3,259	−637
≥ 10%	206	5,468	−1,434

*Source:* Own calculations with the SILC.

*Note:* This table shows the mean of the difference between survey and administrative earnings. A positive mean implies earnings are on average overreported in the survey relative to administrative data. The sample includes those aged 16+ and who overreport their earnings in the survey relative to the administrative data and receive any of UI, UA, or ACLC according to the administrative data in period *t*, while not receiving any of the benefits in period *s* according to both the survey and administrative data. Observations are further split into groups, depending on the survey value of the benefits in period *t*: if a true + for the combined receipt of benefits and the absolute error in the sum of benefits (admin–survey sum of benefits) is *<*/≥ 10 percent of the administrative amount; and a false – for the combined receipt of benefits. Observations with missing/imputed administrative/survey earnings are excluded.

Table 8 examines the relationship between the level of misreporting in earnings and benefits. We combine errors across all three benefits and measure it as the administrative amount minus the survey. The dependent variable is the error in earnings reports measured as the survey minus the administrative amount. A coefficient of 1 would indicate Euro-for-Euro transfer from one source to the other. Column 1 shows that men nearly one-for-one misreport benefits as earnings (and vice versa). We split the benefit reporting errors into four categories: false negatives, false positives, and true positives with benefits underreporting or overreporting. Noting that false negatives are a large group (38.4 percent), the coefficient of 1.072 for men indicates that these individuals report their benefits nearly one-to-one into earnings. False positive men underreport earnings by roughly 60 cents for each Euro overreported. For those who are true positives and underreport their benefit, they appear to report about 2/3 of the missing amount as earnings. For those who overreport their benefit, they underreport earnings by the same amount.

**Table 8.** OLS model of the error in earnings (survey–admin earnings) on the reverse error in sum of benefits (admin–survey benefits).

	Men				Women			
	(1)		(2)		(3)		(4)	
	*β*		*β*		*β*		*β*	
	*(se)*	*ρ*	*(se)*	*ρ*	*(se)*	*ρ*	*(se)*	*ρ*
Constant	5,409.008		5,392.915		3,824.330		3,836.059	
	(1,355.158)	.000	(1,374.510)	.000	(1,108.241)	.001	(1,112.910)	.001
Admin–survey benefits	0.957				0.478			
	(0.096)	.000			(0.069)	.000		
Admin–survey benefits: True –			ref				ref	
False –			1.072				0.537	
			(0.147)	.000			(0.119)	.000
True + & survey underreport			0.660				0.450	
			(0.261)	.011			(0.193)	.020
True + & survey overreport			1.096				0.660	
			(0.186)	.000			(0.107)	.000
False +			0.594				0.272	
			(0.291)	.041			(0.126)	.031
Controls	Yes		Yes		Yes		Yes	
R-squared	.056		.056		.019		.019	
Observations	20,446		20,446		22,771		22,771	

*Source:* Own calculations with the SILC.

*Note:* The outcome variable is the error in earnings (survey–admin earnings). The key independent variable is the reverse error in the sum of all unemployment benefits, UI, UA, and ACLC (admin–survey sum of benefits), overall or by category: with a true – for the combined receipt of all benefits, that is, error equals 0; true +; false –; and a false +. The controls include: age group, number of children in the household, number of adults in the household, if in a couple, region, education, occupation, industry, if a civil servant, country of birth, self-reported health, duration of earnings receipt in months according to the administrative data, proxy interview, month of interview, survey wave, interaction between wave and year, interview type, and if the same interviewer as last year. Sample is restricted to those aged 16+. Observations with missing/imputed administrative/survey earnings/benefits are excluded. Standard errors clustered by individual and shown in parentheses. *p-*values shown next to standard errors and based on a two-tailed significance test.

For women the story is more muted, with smaller coefficients. A similar pattern exists, though: those with false negatives replacing earnings, false positives underreporting earnings, and those who make errors in only the level tend to replace at fractional amounts. There is clearly evidence that people are having difficulty placing income in the proper source. However, we cannot identify if this is simply confusion due to timing, stigma, or other possible explanations.

### Implications of Income Source Confusion

We focus on misreporting of earnings as a dependent variable in two applications. We note that since misreporting of earnings is correlated with variables related to unemployment, coefficients on these variables can be biased. We highlight this using estimates of the returns to education. Since unemployment is associated with lower education, reporting unemployment compensation in earnings inflates earnings disproportionately for that group.

Our second focus is on construction of a sample. When mismeasurement of a key variable in sample construction is correlated with the dependent variable, bias can occur. We highlight this in estimation of the returns to job training. Sample selection hinges on being unemployed in one time period. Along with subsequent misallocation of earnings for those with persistent unemployment, this leads to substantial bias in the estimates of job training.

#### Bias in the returns to education

Table 9 documents the distribution of education by benefit status: “recipient” if receiving any of UI, UA, or ACLC and “nonrecipient” if not receiving unemployment benefits. The key finding is that *recipients* tend to be less educated.

**Table 9.** Education shares (%) by unemployment benefits status.

	Nonrecipients				Recipients			
	Men		Women		Men		Women	
Education	Survey	Admin	Survey	Admin	Survey	Admin	Survey	Admin
Low	9.6	9.3	18.4	18.3	24.7	21.7	29.0	27.8
Middle	67.1	66.6	64.6	64.4	64.0	67.5	58.9	61.0
High	23.3	24.1	17.0	17.2	11.3	10.8	12.1	11.3
Observations	14,434	13,639	16,029	15,570	1,510	2,345	1,280	1,837

*Source:* Own calculations with the SILC.

*Note:* Recipient status based on a receipt of at least one of UI, UA, or ACLC. Education refers to the highest level of education achieved, following on the International Standard Classification of Education (ISCED): *low—*lower secondary or less, that is, compulsory schooling (Pflichtschule); *middle—*upper secondary (Lehre [Berufschule]; Andere berufsbildende mittlere Schule; AHS (Allgeimeinbildende höhere Schule) Oberstufe; berufsbildende mittlere Schule) and post-secondary (Krankenpflegeschule, Berufsbildende höhere Schule—Normalform); *high—*tertiary education including craftsman education and university degree (Meister-, Werkmeisterausbildung; Berufsbildende höhere Schule—Kolleg, Abiturientenlehrgang; Universität, Akademie, Fachhochschule: Erstabschluss; Universität: Doktoratsstudium als Zweitabschluss). Observations with missing/imputed administrative/survey benefits are excluded. Sample is restricted to those aged 19–64.

There is a long-term interest of policymakers and researchers in understanding the association between earnings and education. Given the differences in education by benefits status, we test if the misreporting of unemployment compensation as earnings biases the returns to education. Focusing on full-year, full-time workers, including those who were unemployed but would have worked the full year otherwise, we construct two samples: one using the survey measures of earnings, UI, UA, and ACLC to determine who is a full-year, full-time worker and one using the administrative measures. We estimate standard Mincer wage equations for men and women for survey versus administrative earnings. As in other regressions, we control for a rich set of covariates (see table 10 notes).

**Table 10.** Returns to education: log-earnings regression.

	Men				Women			
	Survey (1)		Admin (2)		Survey (3)		Admin (4)	
	*β*		*β*		*β*		*β*	
	*(se)*	*ρ*	*(se)*	*ρ*	*(se)*	*ρ*	*(se)*	*ρ*
Education: Low	ref		ref		ref		ref	
Middle	0.286		0.320		0.319		0.333	
	(0.025)	.000	(0.033)	.000	(0.027)	.000	(0.037)	.000
High	0.557		0.626		0.621		0.657	
	(0.028)	.000	(0.037)	.000	(0.034)	.000	(0.046)	.000
Constant	10.135		10.125		9.885		9.875	
	(0.053)	.000	(0.070)	.000	(0.075)	.000	(0.105)	.000
Controls	Yes		Yes		Yes		Yes	
R-squared	.272		.325		.298		.339	
Observations	10,020		9,567		5,572		5,309	

*Source:* Own calculations with the SILC.

*Note:* OLS estimation of log-earnings regressions. Sample is restricted to those aged 19–64; and based on survey versus administrative information for full-year and full-time employed or unemployed, but who would have worked the full year otherwise. Education refers to the highest level of education achieved (see table 9 for details). Columns (1) and (3) are based on survey earnings and columns (2) and (4) on administrative earnings. Observations with imputed administrative/survey earnings, UI, UA, or ACLC are excluded. The controls include: age group (5-year bands), region, if a civil servant, country of birth, proxy interview, month of interview, survey wave, interaction between wave and year, interview type, and if the same interviewer as last year. Standard errors clustered by individual and shown in parentheses. *p-*values shown next to standard errors and based on a two-tailed significance test.

In table 10, for *men*, the returns to both middle and high education, relative to low, are higher in the administrative than survey data. Jointly testing the differences in the coefficients between the two datasets rejects the null hypothesis of equivalence (*p-*value of .041). For *women*, we also find differences in the returns to education by dataset, although the differences are smaller and not significantly different (*p-*value of .624). The lower rates of cross-reporting benefits as earnings for women likely drive the weaker results.

Differences in the results from table 10 may stem from two issues. First, the samples are different. The survey data contain individuals who were not working, but reported benefits as labor market earnings, and the administrative data include individuals who failed to report their earnings in the survey. The second bias stems from earnings misreporting: either cross-reporting of benefits as earnings or simple measurement error in earnings alone. Both sample and misreporting errors can include error due to exclusion of parts of true earnings in the administrative earnings. Though we do not know its extent, we show that some of the error is due to cross-reporting. To isolate misreporting of earnings, we repeat the analysis in table 11 by restricting the sample to the same people in both datasets, ensuring that differences in coefficients are entirely due to the intensive misreporting of earnings. For women, most of the difference in the returns to education by dataset goes away, suggesting differences in the sample dominate the results. In comparison, for men the differences mostly remain, so it is differences in the earnings reports that mainly cause the bias in the returns to education.

**Table 11.** Returns to education: log-earnings regression *(sample with both positive administrative and survey earnings).*

	Men						Women					
	Survey (1)		*Intermediate* (2)		Admin (3)		Survey (4)		*Intermediate* (5)		Admin (6)	
	*β*		*β*		*β*		*β*		*β*		*β*	
	*(se)*	*ρ*	*(se)*	*ρ*	*(se)*	*ρ*	*(se)*	*ρ*	*(se)*	*ρ*	*(se)*	*ρ*
Education: Low	ref		ref		ref		ref		ref		ref	
Middle	0.265		0.278		0.283		0.314		0.318		0.308	
	(0.024)	.000	(0.024)	.000	(0.027)	.000	(0.026)	.000	(0.026)	.000	(0.028)	.000
High	0.527		0.550		0.590		0.610		0.624		0.613	
	(0.027)	.000	(0.028)	.000	(0.031)	.000	(0.033)	.000	(0.033)	.000	(0.037)	.000
Constant	10.156		10.149		10.152		9.835		9.848		9.791	
	(0.051)	.000	(0.055)	.000	(0.059)	.000	(0.069)	.000	(0.070)	.000	(0.075)	.000
Controls	Yes		Yes		Yes		Yes		Yes		Yes	
R-squared	.267		.273		.295		.285		.291		.301	
Observations	9,379		9,379		9,379		5,133		5,133		5,133	

*Source and note:* See table 10. Sample is further restricted to those with both positive administrative and survey earnings. Columns (1) and (4) are based on survey earnings; columns (3) and (6) on administrative earnings; and columns (2) and (5) on an “intermediate” earnings measure with administrative earnings for those with administrative receipt of any unemployment benefit—UI, UA, or ACLC—and survey earnings reports for everybody else.

We decompose the errors in earnings using an “intermediate” earnings measure that uses the administrative earnings of those with unemployment benefits and survey earnings reports for everyone else. Differences in coefficients between the survey and intermediate earnings capture the errors from benefit recipients—a combination of cross-reporting and simple misreporting—while differences in coefficients between the intermediate and administrative earnings capture errors from earnings misreporting only. For men, errors from benefit recipients account for a large share of the total bias in the coefficients for middle and high education, suggesting that cross-reporting matters. For women, errors by benefit recipients lead to a downward bias in the returns to education, whereas errors by everybody else go in the opposite direction, though the differences are not statistically significant.

#### Bias in the returns to job training

Policymakers often tie unemployment programs to job training. We look at the implication of errors in survey incomes for the efficacy of job training in raising earnings and employment. Our sample is constructed of individuals who received unemployment benefits or reported being unemployed for at least one month in year *t*. Two samples are constructed: one using only survey measures of UI, UA, and ACLC to establish unemployment and one using administrative measures of UI, UA, and ACLC. The outcome variables are earnings and employment in *t *+* *1, based on survey versus administrative earnings. For the few individuals who received multiple job training spells, we include only the last one.

We estimate a standard Mincer wage regression with indicators for three types of job training: self-funded, employer-funded, and funded by the labor market agency. We control for a rich set of covariates (see table 12 notes). Small sample sizes prevent separate estimation by sex.

**Table 12.** Returns to job training: log-earnings regression.

	Survey (1)		Admin (2)	
	*β*		*β*	
	*(se)*	*ρ*	*(se)*	*ρ*
Job training: Did not take	ref		ref	
Labor market agency	0.143		0.222	
	(0.078)	.068	(0.092)	.016
Mostly paid with own resources	−0.136		−0.314	
	(0.114)	.234	(0.116)	.007
Employer	−0.077		0.152	
	(0.102)	.451	(0.096)	.113
Job-training-year earnings (in thousand)	0.035		0.039	
	(0.002)	.000	(0.002)	.000
Constant	9.334		8.784	
	(0.184)	.000	(0.195)	.000
Controls	Yes		Yes	
R-squared	.497		.513	
Observations	838		1,101	

*Source:* Own calculations with the SILC.

*Note:* OLS estimation with log-earnings in *t + 1* as the outcome variable. Sample is restricted to those aged 19–64; based on survey versus administrative information on being unemployed in *t*, that is, received unemployment benefits (UI, UA, or ACLC) or reported they were unemployed for parts of the year; and with positive survey versus administrative earnings in *t + 1*. We further take for each individual only the last observed year being unemployed and follow-up-year earnings. Column (1) is based on survey and column (2) on administrative earnings, while the indicator for job training is always based on the survey. The controls include: education, age group (5-year bands), region, if a civil servant, country of birth, proxy interview, month of interview, survey wave, interview type, and if the same interviewer as last year. Observations with imputed administrative/survey earnings, UI, UA, or ACLC and missing/n/a job training are excluded. Cells with too few observations cannot be disclosed and are not shown, that is, job training from other institutions. Standard errors shown in parentheses. *p*-values shown next to standard errors and based on a two-tailed significance test.

Table 12 presents the results for earnings. The coefficient on labor market agency training is of particular interest as tied to government policy tackling unemployment. The coefficient in the survey data is a robust 14.3 percent gain from training provided by the labor market agency. However, in the administrative data, this rises to 22.2 percent. The loss of nearly a third of the potential sample is clearly an issue. Additionally, individuals who remain or become unemployed again in year *t *+* *1 may be reporting benefits as earnings. We also note substantial bias in the coefficients on self-funded training (–13.6 percent to –31.4 percent) and employer-funded training (–7.7 percent to 15.2 percent). Joint test for differences in the coefficients between the two datasets rejects the null hypothesis of equivalence (*p-*value of .043).

Table 13 estimates models of employment in period *t *+* *1. In the survey data, the coefficient on labor market agency training is not statistically significant and is 2.4 percent. In the administrative data, the coefficient rises to 9 percent and is significant at the 10 percent level. We note that in tables 4 and 5 those who receive job training are more likely to report UI and UA receipt. We also note that since they are employed in year *t *+* *1, they are less likely to be receiving UI or UA, and thus less likely to be biasing their earnings value. This is consistent with recall bias found in Mathiowetz and Duncan (1988). We jointly test the differences in the coefficients by dataset, rejecting the null hypothesis of equivalence (*p-*value of .043).

**Table 13.** Returns to job training: being an earner regression.

	Survey (1)		Admin (2)	
	*β*		*β*	
	*(se)*	*ρ*	*(se)*	*ρ*
Job training: Did not take	ref		ref	
Labor market agency	0.024		0.090	
	(0.033)	.474	(0.035)	.011
Mostly paid with own resources	−0.043		−0.029	
	(0.050)	.392	(0.044)	.517
Employer	−0.051		0.046	
	(0.050)	.310	(0.041)	.258
Job-training-year earnings (in thousands)	0.008		0.005	
	(0.001)	.000	(0.001)	.000
Constant	0.585		0.698	
	(0.081)	.000	(0.074)	.000
Controls	Yes		Yes	
R-squared	.392		.318	
Observations	1,239		1,451	

*Note:* OLS estimation with being an earner in *t + 1* (yes = 1 and no = 0) as the outcome variable. Sample is restricted to those aged 19–64; based on survey versus administrative information on being unemployed in *t*, that is, received unemployment benefits (UI, UA, or ACLC) or reported they were unemployed for parts of the year. We further take for each individual only the last observed year being unemployed and follow-up-year earner status. Column (1) is based on survey and column (2) on administrative earnings, while the indicator for job training is always based on the survey. The controls include: education, age group (5-year bands), region, if a civil servant, country of birth, proxy interview, month of interview, survey wave, interview type, and if the same interviewer as last year. Observations with missing/imputed administrative/survey earnings, UI, UA, or ACLC and missing/n/a job training are excluded. Cells with too few observations cannot be disclosed and are not shown, that is, job training from other institutions. Standard errors shown in parentheses. *p-*values shown next to standard errors and based on a two-tailed significance test.

Supplementary Material tables S7 and S8 repeat the analysis for earnings and being employed in *t + 1*, respectively, by restricting the sample to the same individuals in both the survey and administrative data. Most of the difference by dataset in the labor market agency coefficient goes away, confirming that it is the difference in the samples that leads to bias in the returns to training.

## Conclusions

Administrative links to survey data have been used to document measurement error in single variables. Few studies (Marquis and Moore 1990; Bollinger and David 2000; Lynn et al. 2012; Balarajan and Collins 2013; Celhay, Meyer, and Mittag 2021) have examined the possibility of program confusion. With exceptions such as Balarajan and Collins (2013), prior work has studied confusion of programs that are very different and hence likely to have low program confusion. We study three Austrian unemployment benefit programs that serve a similar purpose and are administered by the same agency. We both document program confusion across benefits and also demonstrate that benefits may be reported as earnings.

All three Austrian unemployment benefit programs (UI, UA, and ACLC) are underreported. UA and ACLC are the most underreported and are associated with false positives for UI. Even when participation is correctly reported, benefits are still misreported.

Earnings reports are higher compared to administrative data when any of the three programs are underreported, suggesting that respondents know how much income they received, and report much of it as labor market earnings. While we find that both men and women misreport, men seem more likely to misreport benefits as earnings and women more prone to report benefits in the wrong category.

The implications for misreporting in unemployment programs go beyond measurement of unemployment, as misreporting impacts measurement of earnings and construction of analysis samples. The correlation of unemployment and lower education leads to downward bias in estimated returns to education. Additionally, failure to report unemployment benefits can lead to substantial sample bias when selecting on these benefits, biasing job training estimates. The bias is further exacerbated since those with persistent unemployment may misreport continued unemployment as earnings after training occurs.

The implications for our study go well beyond the Austrian unemployment programs or SILC data. If survey respondents provide accurate reports of their total income, but by doing so misclassify benefits as earnings, this can bias estimates of program participation (Bollinger and David 1997), earnings regressions, and estimates of how programs such as UA reduce poverty. Our results suggest that strategies to correct the problem in surveys by replacing only one income component by an accurate measure (e.g., Meyer and Mittag 2019) or taking the maximum of reported and administrative measures appear particularly problematic. Efforts to correct more comprehensive income measures should attempt to correct the sum of these measures rather than each individual one. However, that crucially hinges on distinguishing cross-reporting benefits as earnings from missing earnings in the administrative data. Addressing these issues remains an important agenda for future research.

## Supplementary Material

nfad025_Supplementary_Data

## Data Availability

Replication data are not available because of the permission policy of the original data collector. The editors have waived *POQ*’s replication policy for this manuscript. Please contact the corresponding author for more information. Replication documentation, that is, Stata code, is available in the Supplementary Material.

## Footnotes

1 Match rates for other datasets tend to be lower, for example Bollinger et al. (2019), Meyer and Mittag (2019), Jenkins et al. (2008) and Jenkins and Rios-Avila (2021b).

2 SILC questionnaires are available at https://ec.europa.eu/eurostat/web/income-and-living-conditions/quality/questionnaires. SILC quality reports contain further information on the data, including sampling design and response errors: https://ec.europa.eu/eurostat/web/income-and-living-conditions/quality/eu-and-national-quality-reports.

3 The error in the survey annual benefit amounts can stem from misreporting the benefit duration and/or the average monthly amount. As we are interested in the total error in benefits, as well as in earnings, we do not attempt to disentangle the two error components. Future research will address this.
