# Supplementary material for: Income Source Confusion Using the SILC
Source: Public Opin Q. 2023 Aug 9;87(Suppl 1):542–74. doi: 10.1093/poq/nfad025 (PMC10496567; doi:10.1093/poq/nfad025)
Supplement: nfad025_Supplementary_Data [file nfad025_supplementary_data.pdf]

# Income Source Confusion using the SILC: Supplementary Material

Christopher Robert Bollinger[1] and Iva Valentinova Tasseva[2]

[1]Department of Economics, Gatton College of Business and Economics, University of Kentucky, 550 South Limestone, Lexington, KY 40506-0034, United States, email: crboll@uky.edu

[2]Department of Social Policy, London School of Economics and Political Science, Houghton Street, London, WC2A 2AE, United Kingdom, email: I.Tasseva@lse.ac.uk

# Contents

**Supplementary Tables** **3**

**Stata code** **15**

Description . . . . . 15

Build . . . . . 15

Analysis . . . . . 30

# Supplementary Tables

**Table S1:** Probability of reporting the unemployment insurance (UI) benefit in the survey, conditional on receiving it

| | (1) | | (2) | |
|---|---|---|---|---|
| | $\beta$ (*se*) | *p* | $\beta$ (*se*) | *p* |
| Constant | -.384 | | -.221 | |
| | (.071) | .000 | (.143) | .123 |
| Woman | | | -.034 | |
| | | | (.022) | .122 |
| Ln admin UI | .118 | | .136 | |
| | (.008) | .000 | (.013) | .000 |
| Admin earnings (in thousand) | -.008 | | -.008 | |
| | (.001) | .000 | (.001) | .000 |
| Survey–admin earnings (in thousand) | -.013 | | -.013 | |
| | (.001) | .000 | (.001) | .000 |
| Earnings: true – | ref | | ref | |
| false + | .161 | | .156 | |
| | (.069) | .019 | (.070) | .026 |
| false – | -.048 | | -.015 | |
| | (.045) | .287 | (.045) | .743 |
| true + | .270 | | .257 | |
| | (.036) | .000 | (.037) | .000 |
| UA: true – | ref | | ref | |
| false + | -.143 | | -.152 | |
| | (.080) | .076 | (.087) | .082 |
| false – | -.002 | | -.006 | |
| | (.031) | .940 | (.030) | .854 |
| true + | .064 | | -.017 | |
| | (.036) | .077 | (.038) | .661 |
| ACLC: true – | ref | | ref | |
| false + | .043 | | .006 | |
| | (.048) | .366 | (.048) | .893 |
| Admin benefit duration (in months) | | | -.019 | |
| | | | (.006) | .002 |
| Job training: did not take | | | ref | |
| Labour market agency | | | .112 | |
| | | | (.030) | .000 |
| Mostly paid with own resources | | | .071 | |
| | | | (.037) | .056 |
| Employer | | | .032 | |
| | | | (.031) | .310 |
| Other institutions | | | .041 | |
| | | | (.079) | .600 |
| No proxy | | | ref | |
| partner is proxy | | | -.099 | |
| | | | (.033) | .002 |
| someone else is proxy | | | -.101 | |
| | | | (.032) | .002 |
| Age: 35-39 | | | ref | |
| 16-19 | | | .014 | |
| | | | (.057) | .805 |
| 20-24 | | | -.081 | |
| | | | (.036) | .024 |
| 25-29 | | | -.023 | |
| | | | (.032) | .486 |
| 30-34 | | | -.084 | |
| | | | (.035) | .017 |
| 40-44 | | | -.041 | |
| | | | (.031) | .183 |
| 45-49 | | | -.074 | |
| | | | (.033) | .026 |
| 50-54 | | | -.089 | |
| | | | (.038) | .019 |
| 55-59 | | | -.164 | |
| | | | (.044) | .000 |
| 60-64 | | | -.154 | |

| | | |
|---|---|---|
| | (.074) | .037 |
| Education: low | ref | |
| middle | -.011 | |
| | (.022) | .616 |
| high | .042 | |
| | (.036) | .242 |
| Country of birth: Austria | ref | |
| EU15/EFTA | .040 | |
| | (.045) | .375 |
| new EU12 | .000 | |
| | (.055) | .996 |
| former Yugosl. | -.012 | |
| | (.031) | .710 |
| Turkey | .024 | |
| | (.046) | .603 |
| other | -.071 | |
| | (.045) | .115 |
| In a couple | .024 | |
| | (.027) | .376 |
| No children in the hh | ref | |
| 1 child | .040 | |
| | (.024) | .093 |
| 2 children | -.032 | |
| | (.029) | .270 |
| 3+ children | .047 | |
| | (.042) | .260 |
| 1 adult in the hh | ref | |
| 2 adults | -.085 | |
| | (.031) | .007 |
| 3+ adults | -.065 | |
| | (.032) | .040 |
| Region: Vienna | ref | |
| 100,000+ residents | .053 | |
| | (.036) | .138 |
| 10,000-100,000 residents | .009 | |
| | (.030) | .756 |
| less than 10,000 residents | -.014 | |
| | (.025) | .581 |
| Civil servant: no | ref | |
| missing | .050 | |
| | (.021) | .017 |
| Occupation: elementary | ref | |
| senior officials and managers | -.051 | |
| | (.063) | .412 |
| professionals | -.037 | |
| | (.051) | .475 |
| associate prof. and technical | -.006 | |
| | (.034) | .865 |
| clerks (admin and secretarial) | -.027 | |
| | (.037) | .477 |
| service and sales workers | .001 | |
| | (.032) | .984 |
| skilled agricultural | -.062 | |
| | (.071) | .382 |
| craft and trades workers | -.032 | |
| | (.031) | .312 |
| plant and machine operators | -.016 | |
| | (.036) | .660 |
| n/a | -.015 | |
| | (.046) | .741 |
| missing | .110 | |
| | (.065) | .091 |
| Industry: manufacturing | ref | |
| agriculture | .094 | |
| | (.073) | .195 |
| construction | -.026 | |
| | (.030) | .372 |
| trade | -.024 | |
| | (.031) | .436 |
| transportation | .002 | |
| | (.044) | .966 |
| accommodation and food | .012 | |
| | (.037) | .750 |

| | | |
|---|---|---|
| info and communication | -.078 | |
| | (.059) | .191 |
| real estate | -.054 | |
| | (.065) | .404 |
| other professional etc | -.116 | |
| | (.109) | .286 |
| admin and support services | -.008 | |
| | (.045) | .855 |
| public admin., defence etc | .013 | |
| | (.057) | .826 |
| education | -.009 | |
| | (.059) | .873 |
| health services | -.010 | |
| | (.051) | .847 |
| residential care and social work | -.011 | |
| | (.055) | .848 |
| arts, entertainment etc | .043 | |
| | (.062) | .488 |
| other services | -.077 | |
| | (.083) | .350 |
| n/a | -.142 | |
| | (.045) | .002 |
| Health: very bad | ref | |
| very good | -.056 | |
| | (.083) | .501 |
| good | -.045 | |
| | (.082) | .583 |
| fair | .001 | |
| | (.083) | .992 |
| bad | .016 | |
| | (.087) | .858 |
| Month of interview: Mar | ref | |
| Apr | -.069 | |
| | (.031) | .026 |
| May | -.089 | |
| | (.030) | .003 |
| Jun | -.081 | |
| | (.032) | .012 |
| Jul | -.064 | |
| | (.035) | .065 |
| Aug | -.138 | |
| | (.041) | .001 |
| Sep | -.128 | |
| | (.048) | .007 |
| Oct | -.132 | |
| | (.090) | .144 |
| Interview in person | ref | |
| interview by phone | .134 | |
| | (.046) | .004 |
| Same interviewer: yes | ref | |
| no | -.006 | |
| | (.044) | .898 |
| n/a | .005 | |
| | (.051) | .920 |
| Wave 1 | ref | |
| wave 2 | .019 | |
| | (.047) | .684 |
| wave 3 | -.002 | |
| | (.051) | .967 |
| wave 4 | .049 | |
| | (.053) | .359 |
| Wave 1 × year 2008 | ref | |
| wave=1 × year=2009 | -.001 | |
| | (.074) | .993 |
| wave=1 × year=2010 | .016 | |
| | (.073) | .822 |
| wave=1 × year=2011 | -.042 | |
| | (.075) | .575 |
| wave=2 × year=2009 | -.031 | |
| | (.061) | .614 |
| wave=2 × year=2010 | .017 | |
| | (.062) | .783 |
| wave=2 × year=2011 | -.047 | |

| | | | |
|---|---|---|---|
| | | (.064) | .467 |
| wave=3 × year=2009 | | -.001 | |
| | | (.069) | .993 |
| wave=3 × year=2010 | | .028 | |
| | | (.067) | .680 |
| wave=3 × year=2011 | | -.014 | |
| | | (.066) | .832 |
| wave=4 × year=2009 | | -.137 | |
| | | (.074) | .064 |
| wave=4 × year=2010 | | -.026 | |
| | | (.068) | .705 |
| wave=4 × year=2011 | | -.100 | |
| | | (.071) | .160 |
| R-squared | .137 | .183 | |
| Observations | 3155 | 3155 | |

*Notes:* This table shows an estimation of a linear probability model. The dependent variable equals 1 if the benefit amount is positive in both the survey and administrative data; and 0 if the survey amount is 0 while the administrative amount is positive. 'True +' implies positive income amounts in both the survey and administrative data; 'false +' means positive amount in the survey and zero in the administrative data; 'false –' means zero in the survey and positive amount in the administrative data; and 'true –' means zero amounts in both the survey and administrative data. Sample is restricted to those aged 16+. Observations with missing/imputed administrative/survey UI or earnings are excluded. Cells with too few observations cannot be disclosed and are not shown: UA with missing/imputed values; ACLC false -, true + and with missing/ imputed values; job training with missing or n/a values; age group 65-69; being a civil servant; industry equal to agriculture, mining and quarrying, electricity, gas, steam and air conditioning supply, water supply, sewage, waste management and remediation, finance, real estate, science, other professional, scientific and technical activities, activities of households as employers, undifferentiated goods- and services-producing activities of households for own use and with no answer; occupation equal to armed forces; health status, region and country of birth with missing values; missing or 'don't know' if the same interviewer; and month of interview equal to November. Standard errors clustered by individual and shown in parentheses. P-values shown next to standard errors and based on a two-tailed significance test. *Source:* Own calculations with the SILC.

**Table S2:** Probability of reporting the unemployment insurance (UI) benefit in the survey, conditional on receiving it: **marginal effects from a logit model**

| | (1) | | (2) | |
|---|---|---|---|---|
| | $\beta$ (*se*) | *p* | $\beta$ (*se*) | *p* |
| Woman | | | -.031 | |
| | | | (.022) | .163 |
| Ln admin UI | .119 | | .137 | |
| | (.008) | .000 | (.014) | .000 |
| Admin earnings (in thousand) | -.007 | | -.008 | |
| | (.001) | .000 | (.001) | .000 |
| Survey–admin earnings (in thousand) | -.015 | | -.014 | |
| | (.002) | .000 | (.001) | .000 |
| Earnings: true – | ref | | ref | |
| false + | .163 | | .153 | |
| | (.067) | .015 | (.068) | .025 |
| false – | -.055 | | -.022 | |
| | (.039) | .154 | (.039) | .567 |
| true + | .264 | | .252 | |
| | (.032) | .000 | (.034) | .000 |
| UA: true – | ref | | ref | |
| false + | -.146 | | -.151 | |
| | (.081) | .071 | (.088) | .085 |
| false – | .004 | | .001 | |
| | (.031) | .904 | (.030) | .961 |
| true + | .069 | | -.016 | |
| | (.038) | .068 | (.041) | .691 |
| ACLC: true – | ref | | ref | |
| false + | .053 | | .008 | |
| | (.052) | .310 | (.054) | .884 |
| Admin benefit duration (in months) | | | -.019 | |
| | | | (.007) | .006 |
| Job training: did not take | | | ref | |
| Labour market agency | | | .123 | |
| | | | (.032) | .000 |
| Mostly paid with own resources | | | .070 | |
| | | | (.039) | .074 |
| Employer | | | .040 | |
| | | | (.031) | .207 |
| Other institutions | | | .042 | |
| | | | (.084) | .615 |
| No proxy | | | ref | |
| partner is proxy | | | -.097 | |
| | | | (.032) | .003 |
| someone else is proxy | | | -.099 | |
| | | | (.031) | .001 |
| Controls | No | | Yes | |
| Observations | 3150 | | 3136 | |

*Notes:* The dependent variable equals 1 if the benefit amount is positive in both the survey and administrative data; and 0 if the survey amount is 0 while the administrative amount is positive. 'True +' implies positive income amounts in both the survey and administrative data; 'false +' means positive amount in the survey and zero in the administrative data; 'false –' means zero in the survey and positive amount in the administrative data; and 'true –' means zero amounts in both the survey and administrative data. Column (2) adds controls for: age group (in 5-year age bands), number of children in the household (0, 1, 2, 3+), number of adults in the household (1, 2, 3+), the highest achieved education level (low, middle, high), if in a couple, health status (6 categories), region (Vienna, borough with more than 100,000 residents, borough with 10,000-100,000 residents, borough with less than 10,000 residents), occupation (12 categories), industry (25 categories), being a civil servant, country of birth (7 categories), wave (interviewed for the 1st, 2nd, 3rd, 4th time), interaction between wave and year (2008 to 2011), interview type (in person or by phone), same interviewer as last year. Sample is restricted to those aged 16+. Observations with missing/imputed administrative/survey UI or earnings are excluded. Cells with too few observations cannot be disclosed and are not shown: UA with missing/imputed values; ACLC false -, true + and with missing/imputed values; and job training with missing or n/a values. Standard errors clustered by individual and shown in parentheses. P-values shown next to standard errors and based on a two-tailed significance test. *Source:* Own calculations with the SILC.

**Table S3:** Probability of reporting the unemployment assistance (UA) in the survey, conditional on receiving it

| | (1) | | (2) | |
|---|---|---|---|---|
| | $\beta$ (*se*) | *p* | $\beta$ (*se*) | *p* |
| Constant | -.109 | | .459 | |
| | (.093) | .242 | (.168) | .006 |
| Woman | | | -.023 | |
| | | | (.032) | .480 |
| Ln admin UA | .094 | | .035 | |
| | (.011) | .000 | (.015) | .022 |
| Admin earnings (in thousand) | -.012 | | -.010 | |
| | (.003) | .000 | (.003) | .004 |
| Survey–admin earnings (in thousand) | -.015 | | -.013 | |
| | (.002) | .000 | (.002) | .000 |
| Earnings: true – | ref | | ref | |
| false + | -.279 | | -.247 | |
| | (.057) | .000 | (.058) | .000 |
| false – | .023 | | .017 | |
| | (.042) | .584 | (.044) | .694 |
| true + | .011 | | -.007 | |
| | (.042) | .794 | (.046) | .880 |
| UI: true – | ref | | ref | |
| false + | -.312 | | -.298 | |
| | (.045) | .000 | (.046) | .000 |
| false – | -.107 | | -.052 | |
| | (.038) | .005 | (.039) | .176 |
| true + | -.058 | | -.049 | |
| | (.036) | .106 | (.039) | .215 |
| missing/imputed | -.039 | | -.092 | |
| | (.045) | .376 | (.043) | .033 |
| ACLC: true – | ref | | ref | |
| false + | .177 | | .034 | |
| | (.042) | .000 | (.048) | .477 |
| Admin benefit duration (in months) | | | .015 | |
| | | | (.006) | .013 |
| Job training: did not take | | | ref | |
| Labour market agency | | | .181 | |
| | | | (.037) | .000 |
| Mostly paid with own resources | | | .029 | |
| | | | (.071) | .682 |
| Employer | | | -.065 | |
| | | | (.067) | .335 |
| No proxy | | | ref | |
| partner is proxy | | | -.078 | |
| | | | (.065) | .227 |
| someone else is proxy | | | .027 | |
| | | | (.049) | .582 |
| Age: 35-39 | | | ref | |
| 16-19 | | | -.150 | |
| | | | (.102) | .144 |
| 20-24 | | | -.036 | |
| | | | (.059) | .542 |
| 25-29 | | | -.036 | |
| | | | (.053) | .499 |
| 30-34 | | | -.039 | |
| | | | (.051) | .446 |
| 40-44 | | | .050 | |
| | | | (.049) | .309 |
| 45-49 | | | -.023 | |
| | | | (.054) | .673 |
| 50-54 | | | -.014 | |
| | | | (.053) | .799 |
| 55-59 | | | -.106 | |
| | | | (.058) | .067 |
| 60-64 | | | -.089 | |
| | | | (.098) | .364 |
| Education: low | | | ref | |
| middle | | | -.046 | |
| | | | (.030) | .129 |
| high | | | -.015 | |

| | | |
|---|---|---|
| | (.052) | .776 |
| Country of birth: Austria | ref | |
| new EU12 | -.105 | |
| | (.076) | .169 |
| former Yugosl. | -.062 | |
| | (.052) | .233 |
| Turkey | -.055 | |
| | (.055) | .319 |
| other | .035 | |
| | (.053) | .518 |
| In a couple | -.094 | |
| | (.038) | .013 |
| No children in the hh | ref | |
| 1 child | .051 | |
| | (.038) | .178 |
| 2 children | .055 | |
| | (.045) | .219 |
| 3+ children | .144 | |
| | (.061) | .019 |
| 1 adult in the hh | ref | |
| 2 adults | .018 | |
| | (.043) | .673 |
| 3+ adults | -.097 | |
| | (.048) | .042 |
| Region: Vienna | ref | |
| 100,000+ residents | -.021 | |
| | (.051) | .678 |
| 10,000-100,000 residents | .015 | |
| | (.040) | .696 |
| less than 10,000 residents | -.044 | |
| | (.036) | .227 |
| Civil servant: no | ref | |
| missing | .072 | |
| | (.035) | .042 |
| Occupation: elementary | ref | |
| senior officials and managers | .041 | |
| | (.088) | .638 |
| professionals | .017 | |
| | (.073) | .820 |
| associate prof. and technical | -.040 | |
| | (.055) | .464 |
| clerks (admin and secretarial) | .013 | |
| | (.056) | .814 |
| service and sales workers | -.032 | |
| | (.041) | .431 |
| skilled agricultural | .123 | |
| | (.110) | .262 |
| craft and trades workers | .020 | |
| | (.046) | .671 |
| plant and machine operators | -.030 | |
| | (.055) | .579 |
| n/a | -.135 | |
| | (.074) | .067 |
| missing | .036 | |
| | (.072) | .617 |
| Industry: manufacturing | ref | |
| construction | .012 | |
| | (.048) | .800 |
| trade | -.058 | |
| | (.044) | .184 |
| transportation | -.073 | |
| | (.066) | .274 |
| accommodation and food | -.019 | |
| | (.056) | .733 |
| info and communication | -.008 | |
| | (.084) | .928 |
| admin and support services | -.089 | |
| | (.066) | .177 |
| public admin., defence etc | .005 | |
| | (.095) | .961 |
| education | -.156 | |
| | (.083) | .058 |
| health services | -.012 | |

| | | | |
|---|---|---|---|
| | | (.091) | .896 |
| residential care and social work | | .033 | |
| | | (.078) | .673 |
| arts, entertainment etc | | -.202 | |
| | | (.100) | .043 |
| other services | | -.048 | |
| | | (.075) | .519 |
| n/a | | -.147 | |
| | | (.074) | .046 |
| Health: very bad | | ref | |
| very good | | -.011 | |
| | | (.070) | .871 |
| good | | .045 | |
| | | (.067) | .504 |
| fair | | .065 | |
| | | (.064) | .309 |
| bad | | .010 | |
| | | (.063) | .874 |
| Month of interview: Mar | | ref | |
| Apr | | -.030 | |
| | | (.052) | .569 |
| May | | .017 | |
| | | (.046) | .713 |
| Jun | | -.091 | |
| | | (.049) | .065 |
| Jul | | -.027 | |
| | | (.054) | .624 |
| Aug | | -.094 | |
| | | (.058) | .104 |
| Sep | | .011 | |
| | | (.068) | .870 |
| Interview in person | | ref | |
| interview by phone | | .151 | |
| | | (.067) | .025 |
| Same interviewer: yes | | ref | |
| no | | -.121 | |
| | | (.058) | .036 |
| n/a | | -.121 | |
| | | (.080) | .133 |
| Wave 1 | | ref | |
| wave 2 | | -.068 | |
| | | (.067) | .312 |
| wave 3 | | -.101 | |
| | | (.075) | .178 |
| wave 4 | | -.014 | |
| | | (.078) | .859 |
| Wave 1 × year 2008 | | ref | |
| wave=1 × year=2009 | | -.074 | |
| | | (.105) | .485 |
| wave=1 × year=2010 | | -.049 | |
| | | (.106) | .643 |
| wave=1 × year=2011 | | .070 | |
| | | (.108) | .514 |
| wave=2 × year=2009 | | -.014 | |
| | | (.083) | .864 |
| wave=2 × year=2010 | | .052 | |
| | | (.083) | .531 |
| wave=2 × year=2011 | | -.025 | |
| | | (.089) | .779 |
| wave=3 × year=2009 | | .107 | |
| | | (.092) | .247 |
| wave=3 × year=2010 | | .101 | |
| | | (.091) | .267 |
| wave=3 × year=2011 | | .031 | |
| | | (.094) | .740 |
| wave=4 × year=2009 | | -.159 | |
| | | (.104) | .127 |
| wave=4 × year=2010 | | -.146 | |
| | | (.094) | .119 |
| wave=4 × year=2011 | | -.083 | |
| | | (.097) | .391 |
| R-squared | .234 | .298 | |
| Observations | 1262 | 1262 | |

*Notes and Source:* See Table S1. Observations with missing/imputed administrative/survey UA or earnings are excluded. Cells with too few observations cannot be disclosed and are not shown: ACLC false -, true + and with missing/imputed values; job training by other institutions and with n/a values; age group 65-69; country birth equal to EU15/EFTA; being a civil servant; industry equal to agriculture, mining and quarrying, electricity, gas, steam and air conditioning supply, water supply, sewage, waste management and remediation, information and communication, finance, real estate, other professional, scientific and technical activities, activities of households as employers, undifferentiated goods- and services-producing activities of households for own use and with no answer; occupation equal to armed forces; and month of interview equal to October and November.

**Table S4:** Probability of reporting the unemployment assistance (UA) in the survey, conditional on receiving it: **marginal effects from a logit model**

| | (1) | | (2) | |
|---|---|---|---|---|
| | $\beta$ (*se*) | *p* | $\beta$ (*se*) | *p* |
| Woman | | | -.019 | |
| | | | (.031) | .543 |
| Ln admin UA | .098 | | .035 | |
| | (.012) | .000 | (.016) | .031 |
| Admin earnings (in thousand) | -.016 | | -.012 | |
| | (.004) | .000 | (.004) | .001 |
| Survey–admin earnings (in thousand) | -.028 | | -.023 | |
| | (.004) | .000 | (.004) | .000 |
| Earnings: true – | ref | | ref | |
| false + | -.201 | | -.219 | |
| | (.068) | .003 | (.064) | .001 |
| false – | .009 | | -.006 | |
| | (.038) | .821 | (.039) | .869 |
| true + | .091 | | .048 | |
| | (.038) | .016 | (.041) | .237 |
| UI: true – | ref | | ref | |
| false + | -.297 | | -.296 | |
| | (.041) | .000 | (.040) | .000 |
| false – | -.107 | | -.073 | |
| | (.040) | .008 | (.039) | .059 |
| true + | -.063 | | -.074 | |
| | (.036) | .080 | (.036) | .040 |
| missing/imputed | -.040 | | -.094 | |
| | (.043) | .363 | (.043) | .027 |
| ACLC: true – | ref | | ref | |
| false + | .152 | | .031 | |
| | (.041) | .000 | (.045) | .496 |
| Admin benefit duration (in months) | | | .012 | |
| | | | (.006) | .027 |
| Job training: did not take | | | ref | |
| Labour market agency | | | .166 | |
| | | | (.036) | .000 |
| Mostly paid with own resources | | | .072 | |
| | | | (.069) | .299 |
| Employer | | | -.085 | |
| | | | (.088) | .334 |
| No proxy | | | ref | |
| partner is proxy | | | -.061 | |
| | | | (.060) | .313 |
| someone else is proxy | | | .041 | |
| | | | (.044) | .352 |
| Controls | No | | Yes | |
| Observations | 1261 | | 1254 | |

*Notes and Source:* See Table S2. Observations with missing/imputed administrative/survey UA or earnings are excluded. Cells with too few observations cannot be disclosed and are not shown: ACLC false -, true + and with missing/ imputed values; and job training by other institutions and with n/a values.

**Table S5:** Probability of reporting the unemployment insurance (UI) benefit in the survey, conditional on receiving it, by gender

| | Men | | | | Women | | | |
|---|---|---|---|---|---|---|---|---|
| | (1) | | (2) | | (3) | | (4) | |
| | $\beta$ (*se*) | *p* | $\beta$ (*se*) | *p* | $\beta$ (*se*) | *p* | $\beta$ (*se*) | *p* |
| Constant | -.535 | | -.337 | | -.231 | | -.123 | |
| | (.095) | .000 | (.191) | .078 | (.106) | .029 | (.218) | .573 |
| Ln admin UI | .123 | | .135 | | .111 | | .138 | |
| | (.011) | .000 | (.017) | .000 | (.012) | .000 | (.019) | .000 |
| Admin earnings (in thousand) | -.008 | | -.008 | | -.010 | | -.012 | |
| | (.001) | .000 | (.001) | .000 | (.002) | .000 | (.002) | .000 |
| Survey–admin earnings (in thousand) | -.012 | | -.012 | | -.015 | | -.016 | |
| | (.001) | .000 | (.001) | .000 | (.003) | .000 | (.003) | .000 |
| Earnings: true − | ref | | ref | | ref | | ref | |
| false + | .345 | | .310 | | -.058 | | -.016 | |
| | (.085) | .000 | (.091) | .001 | (.108) | .592 | (.115) | .891 |
| false − | .038 | | .056 | | -.123 | | -.071 | |
| | (.065) | .555 | (.064) | .387 | (.063) | .049 | (.063) | .266 |
| true + | .380 | | .354 | | .186 | | .185 | |
| | (.051) | .000 | (.057) | .000 | (.048) | .000 | (.051) | .000 |
| UA: true − | ref | | ref | | ref | | ref | |
| false − | .059 | | .059 | | -.082 | | -.079 | |
| | (.042) | .159 | (.041) | .148 | (.046) | .074 | (.047) | .094 |
| true + | .047 | | -.025 | | .083 | | -.009 | |
| | (.055) | .394 | (.058) | .663 | (.047) | .082 | (.052) | .859 |
| ACLC: true − | ref | | ref | | ref | | ref | |
| false + | .117 | | .052 | | -.007 | | -.021 | |
| | (.065) | .074 | (.069) | .446 | (.066) | .920 | (.068) | .761 |
| Admin benefit duration (in months) | | | -.015 | | | | -.026 | |
| | | | (.008) | .082 | | | (.010) | .006 |
| Job training: did not take | | | ref | | | | ref | |
| Labour market agency | | | .176 | | | | .071 | |
| | | | (.043) | .000 | | | (.045) | .112 |
| Mostly paid with own resources | | | .023 | | | | .117 | |
| | | | (.052) | .654 | | | (.056) | .038 |
| Employer | | | .016 | | | | .065 | |
| | | | (.039) | .681 | | | (.054) | .231 |
| No proxy | | | ref | | | | ref | |
| partner is proxy | | | -.084 | | | | -.160 | |
| | | | (.039) | .032 | | | (.066) | .015 |
| someone else is proxy | | | -.109 | | | | -.064 | |
| | | | (.041) | .008 | | | (.053) | .220 |
| Controls | No | | Yes | | No | | Yes | |
| R-squared | .146 | | .184 | | .127 | | .180 | |
| Observations | 1856 | | 1856 | | 1299 | | 1299 | |

*Notes and Source:* See Table S1. Cells with too few observations cannot be disclosed and are not shown: UA false + and with missing/imputed values; ACLC false -, true + and with missing/imputed values; and job training by other institutions and with missing or n/a values. Standard errors clustered by individual and shown in parentheses. P-values shown next to standard errors and based on a two-tailed significance test.

**Table S6:** Probability of reporting the unemployment assistance (UA) in the survey, conditional on receiving it, by gender

| | Men | | | | Women | | | |
|---|---|---|---|---|---|---|---|---|
| | (1) | | (2) | | (3) | | (4) | |
| | $\beta$ (*se*) | *p* | $\beta$ (*se*) | *p* | $\beta$ (*se*) | *p* | $\beta$ (*se*) | *p* |
| Constant | -.267 | | .112 | | .037 | | .952 | |
| | (.147) | .069 | (.252) | .657 | (.125) | .767 | (.244) | .000 |
| Ln admin UA | .108 | | .061 | | .082 | | -.006 | |
| | (.016) | .000 | (.022) | .006 | (.015) | .000 | (.024) | .798 |
| Admin earnings (in thousand) | -.013 | | -.008 | | -.011 | | -.010 | |
| | (.003) | .000 | (.004) | .052 | (.006) | .051 | (.007) | .168 |
| Survey–admin earnings (in thousand) | -.014 | | -.011 | | -.018 | | -.017 | |
| | (.003) | .000 | (.003) | .000 | (.004) | .000 | (.004) | .000 |
| Earnings: true – | ref | | ref | | ref | | ref | |
| false + | -.296 | | -.245 | | -.239 | | -.169 | |
| | (.065) | .000 | (.078) | .002 | (.102) | .019 | (.099) | .088 |
| false – | .122 | | .097 | | -.116 | | -.100 | |
| | (.054) | .024 | (.062) | .118 | (.069) | .095 | (.070) | .156 |
| true + | .043 | | .017 | | -.026 | | -.009 | |
| | (.061) | .488 | (.072) | .817 | (.059) | .659 | (.064) | .894 |
| UI: true – | ref | | ref | | ref | | ref | |
| false + | -.287 | | -.298 | | -.347 | | -.324 | |
| | (.059) | .000 | (.060) | .000 | (.070) | .000 | (.072) | .000 |
| false – | -.044 | | -.029 | | -.178 | | -.111 | |
| | (.051) | .391 | (.058) | .617 | (.056) | .001 | (.057) | .052 |
| true + | -.073 | | -.070 | | -.023 | | -.037 | |
| | (.049) | .141 | (.057) | .218 | (.055) | .677 | (.062) | .553 |
| missing/imputed | -.073 | | -.101 | | -.018 | | -.105 | |
| | (.076) | .339 | (.075) | .180 | (.057) | .746 | (.060) | .078 |
| ACLC: true – | ref | | ref | | ref | | ref | |
| false + | .227 | | .081 | | .108 | | -.014 | |
| | (.052) | .000 | (.063) | .199 | (.068) | .112 | (.076) | .848 |
| Admin benefit duration (in months) | | | .013 | | | | .021 | |
| | | | (.009) | .127 | | | (.010) | .036 |
| Job training: did not take | | | ref | | | | ref | |
| Labour market agency | | | .186 | | | | .214 | |
| | | | (.051) | .000 | | | (.058) | .000 |
| Mostly paid with own resources | | | -.013 | | | | .069 | |
| | | | (.105) | .903 | | | (.107) | .518 |
| No proxy | | | ref | | | | ref | |
| partner is proxy | | | -.098 | | | | -.163 | |
| | | | (.091) | .280 | | | (.109) | .136 |
| someone else is proxy | | | .073 | | | | -.065 | |
| | | | (.071) | .307 | | | (.072) | .368 |
| Controls | No | | Yes | | No | | Yes | |
| R-squared | .282 | | .343 | | .191 | | .266 | |
| Observations | 678 | | 678 | | 584 | | 584 | |

*Notes and Source:* See Table S5. Observations with missing/imputed administrative/survey UA or earnings are excluded. Cells with too few observations cannot be disclosed and are not shown: ACLC false -, true + and with missing/imputed values; and job training by employer, other institutions and with missing or n/a values.

**Table S7:** Returns to job training: log-earnings regression
*(restricted to the same sample in the survey and administrative data)*

| | **Survey** (1) | | **Admin** (2) | |
|---|---|---|---|---|
| | $\beta$ (*se*) | *p* | $\beta$ (*se*) | *p* |
| Job training: did not take | ref | | ref | |
| **Labour market agency** | .195 | | .216 | |
| | (.089) | .028 | (.091) | .018 |
| Mostly paid with own resources | -.125 | | -.185 | |
| | (.122) | .307 | (.125) | .139 |
| Employer | -.099 | | .099 | |
| | (.109) | .362 | (.109) | .362 |
| Job-training-year earnings (in thousand) | .035 | | .039 | |
| | (.003) | .000 | (.003) | .000 |
| Constant | 9.236 | | 9.235 | |
| | (.200) | .000 | (.205) | .000 |
| Controls | Yes | | Yes | |
| R-squared | .493 | | .539 | |
| Observations | 667 | | 667 | |

*Notes:* OLS estimation with log-earnings in *t+1* as the outcome variable. Sample is restricted to those aged 19-64; based on survey versus administrative information on being unemployed in *t*, i.e. received unemployment benefits (UI, UA or ACLC) or reported they were unemployed for parts of the year; and with both positive survey and administrative earnings in *t+1* and non-missing, non-imputed survey and administrative job-training-year earnings. We further take for each individual only the last observed year being unemployed and follow-up-year earnings. Column (1) is based on survey and column (2) on administrative earnings while the indicator for job training is always based on the survey. The controls include: education, age group (5-year bands), region, if a civil servant, country of birth, proxy interview, month of interview, survey wave, interview type, and if the same interviewer as last year. Observations with imputed administrative/survey earnings, UI, UA or ACLC and missing/n/a job training are excluded. Cells with too few observations cannot be disclosed and are not shown, i.e. job training from other institutions. Standard errors shown in parentheses. P-values shown next to standard errors and based on a two-tailed significance test. *Source:* Own calculations with the SILC.

**Table S8:** Returns to job training: being an earner regression
*(restricted to the same sample in the survey and administrative data)*

| | **Survey** (1) | | **Admin** (2) | |
|---|---|---|---|---|
| | $\beta$ (*se*) | *p* | $\beta$ (*se*) | *p* |
| Job training: did not take | ref | | ref | |
| **Labour market agency** | .074 | | .069 | |
| | (.039) | .060 | (.039) | .078 |
| Mostly paid with own resources | -.073 | | -.017 | |
| | (.054) | .173 | (.054) | .754 |
| Employer | -.037 | | .022 | |
| | (.055) | .503 | (.055) | .693 |
| Job-training-year earnings (in thousand) | .009 | | .004 | |
| | (.001) | .000 | (.001) | .000 |
| Constant | .575 | | .703 | |
| | (.088) | .000 | (.089) | .000 |
| Controls | Yes | | Yes | |
| R-squared | .390 | | .335 | |
| Observations | 1048 | | 1048 | |

*Notes:* OLS estimation with being an earner in *t+1* (yes=1 and no=0) as the outcome variable. Sample is restricted to those aged 19-64; based on survey versus administrative information on being unemployed in *t*, i.e. received unemployment benefits (UI, UA or ACLC) or reported they were unemployed for parts of the year; and with both non-missing/non-imputed survey and administrative outcome variable and job-training-year earnings. We further take for each individual only the last observed year being unemployed and follow-up-year earner status. Column (1) is based on survey and column (2) on administrative earnings while the indicator for job training is always based on the survey. The controls include: education, age group (5-year bands), region, if a civil servant, country of birth, proxy interview, month of interview, survey wave, interview type, and if the same interviewer as last year. Observations with missing/imputed administrative/survey earnings, UI, UA or ACLC and missing/n/a job training are excluded. Cells with too few observations cannot be disclosed and are not shown, i.e. job training from other institutions. Standard errors shown in parentheses. P-values shown next to standard errors and based on a two-tailed significance test.

# Stata code

## Description

```
PROJECT:
Income Source Confusion using the SILC

AUTHORS:
Christopher R. Bollinger and Iva V. Tasseva

DESCRIPTION:
- Build folder contains do-files that clean the data and build a data set for analysis.
    See master do-file master_build.
- Analysis folder contains do-files that carry out the analysis and produce all tables in
     the paper and online supplementary materials. See master do-file master_analysis.
```

## Build

### master_build.do

```
*****************************************************************************************
* PROJECT:                Income Source Confusion using the SILC
* AUTHORS:                             Christopher R. Bollinger and Iva V. Tasseva
* DESCRIPTION:                  master do-file to clean data and prepare data set for
    analysis
*****************************************************************************************
clear all
clear matrix
set more off
set mem 32g
set type double
macro drop _all
set matsize 11000

* define locations // TO DO
global main = ""                              // main Build folder
// subfolders
global do ""                                  // do-files
global log ""                                 // log-files
global input ""                               // raw SILC data
global output ""                              // cleaned data
global temp ""                                // temporary datasets
global analysis_input ""              // final data for Analysis (subfolder input of
    folder Analysis)

set more off
* combine the individual and household-level data sets from all years into two datasets:
    1) based on survey data only and 2) based on survey and admin data
do "${do}\combine_datasets.do"

* create dataset with annual survey and admin amounts of earnings and benefits
do "${do}\prepare_income_data.do"

* prepare data set that will be used for the analysis
do "${do}\prepare_data_analysis.do"
```

### combine_datasets.do

```
set more off
cap log close
log using "${log}\log_combine_datasets.log", replace
*****************************************************************************************
* combine the individual and household-level data sets from all years into two datasets
    based on:
*  1) survey data only and 2) survey and admin data
*************************************************************************
// Define globals for data location
global input_data2008 "${input}\2008\"                          // admin incomes
global input_data2008vr "${input}\2008vorRev\"          // survey incomes (before
    revision, 'vor Revision')
global input_data2009 "${input}\2009\"
global input_data2009vr "${input}\2009vorRev\"
```

```
global input_data2010 "${input}\2010\"
global input_data2010vr "${input}\2010vorRev\"
global input_data2011 "${input}\2011\"
global input_data2011vr "${input}\2011vorRev\"
global waves 2008 2009 2010 2011 // long names for waves
global sh_waves 08 09 10 11 // short names for waves

// Define globals for datasets names
foreach wave of global waves {
                                            // main SILC files
        * Hosehold Register (D-FILE)
        global d`wave'_file "d_silc`wave'_ext"

        * Personal Register (R-FILE), i.e. all people
        global r`wave'_file "r_silc`wave'_ext"

        * Household Data (H-FILE)
        global h`wave'_file "h_silc`wave'_ext"

        * Personal Data (P-FILE) for those aged 16 and above
        global p`wave'_file "p_silc`wave'_ext"

        * Child Data (K-FILE) for those aged below 16
        global k`wave'_file "k_silc`wave'_ext"
}

* Household key (Schluessel D)
                                            // longitudinal ids
global d_keyfile "id_schluessel_d_ext"
                                    // admin data
global d_keyfile2 "id_schl ssel_d_ext"
                                    // survey data with umlaut

* Person key (Schluessel R)
global r_keyfile "id_schluessel_r_ext"
global r_keyfile2 "id_schl ssel_r_ext"

* Hosehold (H) and Personal (P) additional data ('Zusatzdaten')
            // detailed data on benefits and earnings
foreach sh_wave of global sh_waves {
        * Household Data (H-FILE)
        global h20`sh_wave'_addfile "h_file`sh_wave'_zusatz"

        * Personal Data (P-FILE), i.e. people aged 16 and above
        global p20`sh_wave'_addfile "p_file`sh_wave'_zusatz"
}

// change data format - destring values
foreach type in d r h p k {
        foreach wave of global waves {
                insheet using "${input_data`wave'}\${`type'`wave'_file}.csv", clear
                            // admin data
                destring _all, replace
                format _all %15.0g
                save "${temp}\${`type'`wave'_file}.dta", replace

                insheet using "${input_data`wave'vr}\${`type'`wave'_file}.csv", clear
                    // survey data
                destring _all, replace
                format _all %15.0g
                save "${temp}\${`type'`wave'_file}vr.dta", replace
        }
}
foreach wave of global waves {
                                            // admin data
        insheet using "${input_data`wave'}\${d_keyfile}.csv", clear
        destring _all, replace
        format _all %15.0g
        save "${temp}\${d_keyfile}`wave'.dta", replace

        insheet using "${input_data`wave'}\${r_keyfile}.csv", clear

        destring _all, replace
```

```
        format _all %15.0g
        save "${temp}\${r_keyfile}`wave'.dta", replace
}
foreach wave of global waves {
                                                  // survey data
        insheet using "${input_data`wave'vr}\${d_keyfile2}.csv", clear

        destring _all, replace
        format _all %15.0g
        save "${temp}\${d_keyfile}`wave'vr.dta", replace

        insheet using "${input_data`wave'vr}\${r_keyfile2}.csv", clear
        destring _all, replace
        format _all %15.0g
        save "${temp}\${r_keyfile}`wave'vr.dta", replace
}

* additional data - Zusatzdaten
foreach type in h p {
        foreach wave of global waves {
                insheet using "${input_data`wave'}\${`type'`wave'_addfile}.csv", clear
                    // admin data
                destring _all, replace
                format _all %15.0g
                save "${temp}\${`type'`wave'_addfile}.dta", replace

                insheet using "${input_data`wave'vr}\${`type'`wave'_addfile}.csv", clear
                    // survey data
                destring _all, replace
                format _all %15.0g
                save "${temp}\${`type'`wave'_addfile}vr.dta", replace
        }
}

foreach wave in 2009 {
        insheet using "${input_data`wave'vr}\${p`wave'_addfile}.csv", clear
        destring _all, replace
        format _all %15.0g

        * wrong personal ids for 3 observations; corrected their values based on their
            incomes in the different datasets
        replace lnpkzqu=328504 if lnpkzqu==328501 & p042003>0
        replace lnpkzqu=623002 if lnpkzqu==623001 & p042003==-2
        replace lnpkzqu=543002 if lnpkzqu==543001 & p070024==-2
        save "${temp}\${p`wave'_addfile}vr.dta", replace
}

// Merge household and personal files into one file per wave

* admin data
foreach wave of global waves {
        * Household and Personal Register
        use "${temp}\${d`wave'_file}.dta", clear
        sort ln // household id
        * weights are missing in the d file - so drop them and take the value from the
            other datasets
        drop hgew
        merge m:m ln using "${temp}\${r`wave'_file}.dta"
        drop _merge

        * Household Data
        sort ln lnpkzqu // households and pesonal id
        merge m:m ln using "${temp}\${h`wave'_file}.dta"
        drop _merge
        sort ln lnpkzqu

        * Child Data
        sort ln lnpkzqu
        merge m:m ln lnpkzqu using "${temp}\${k`wave'_file}.dta"
        drop _merge
        sort ln lnpkzqu

        * Personal Data
```

```
        sort ln lnpkzqu
        merge m:m ln lnpkzqu using "${temp}\${p`wave'_file}.dta"
        tab _merge
        drop _merge
        sort ln lnpkzqu

        * Additional Household Data - with longitudinal hh identifier
        sort ln lnpkzqu
        merge m:m ln using "${temp}\${d_keyfile}`wave'.dta"
        drop if _merge==2
        drop _merge
        sort ln lnpkzqu

        * Additional Personal Data - with longitudinal hh and personal identifier
        sort ln lnpkzqu
        merge m:m ln lnpkzqu using "${temp}\${r_keyfile}`wave'.dta"
        drop if _merge==2
        drop _merge
        sort ln lnpkzqu

        * Additional Household Data - with additional data on incomes
        sort ln lnpkzqu
        merge m:m ln using "${temp}\${h`wave'_addfile}.dta"
        drop if _merge==2
        drop _merge
        sort ln lnpkzqu

        * Additional Personal Data - with additional data on incomes
        sort ln lnpkzqu
        merge m:m ln lnpkzqu using "${temp}\${p`wave'_addfile}.dta"
        drop if _merge==2
        drop _merge
        sort ln lnpkzqu

        gen year = `wave'

        save "${temp}\main_`wave'.dta", replace
}

* survey data
foreach wave of global waves {
        * Household & Personal Register
        use "${temp}\${d`wave'_file}vr.dta", clear
        sort ln
        merge m:m ln using "${temp}\${r`wave'_file}vr.dta"
        drop _merge

        * Household Data
        sort ln lnpkzqu
        merge m:m ln using "${temp}\${h`wave'_file}vr.dta"
        drop _merge
        sort ln lnpkzqu

        * Child Data
        sort ln lnpkzqu
        merge m:m ln lnpkzqu using "${temp}\${k`wave'_file}vr.dta"
        drop _merge
        sort ln lnpkzqu

        * Personal Data
        sort ln lnpkzqu
        merge m:m ln lnpkzqu using "${temp}\${p`wave'_file}vr.dta"
        tab _merge
        drop _merge
        sort ln lnpkzqu

        * Additional Household Data - with longitudinal hh identifier
        sort ln lnpkzqu
        merge m:m ln using "${temp}\${d_keyfile}`wave'vr.dta"
        drop if _merge==2
        drop _merge
        sort ln lnpkzqu
```

```
        * Additional Personal Data − with longitudinal hh and personal identifier
        sort ln lnpkzqu
        merge m:m ln lnpkzqu using ”${temp}\${r_keyfile}‘wave’vr.dta”
        drop if _merge==2
        drop _merge
        sort ln lnpkzqu

        * Additional Household Data − with additional data on incomes
        sort ln lnpkzqu
        merge m:m ln using ”${temp}\${h‘wave’_addfile}vr.dta”
        drop if _merge==2
        drop _merge
        sort ln lnpkzqu

        * Additional Personal Data − with additional data on incomes
        sort ln lnpkzqu
        merge m:m ln lnpkzqu using ”${temp}\${p‘wave’_addfile}vr.dta”
        drop if _merge==2
        drop _merge
        sort ln lnpkzqu

        gen year = ‘wave’

        save ”${temp}\main_‘wave’vr.dta”, replace
}

* append datasets with administrative data
global waves 2008
foreach wave of global waves {
        use ”${temp}\main_‘wave’.dta”, clear
}

global waves 2009 2010 2011
foreach wave of global waves {
        append using ”${temp}\main_‘wave’.dta”
}

save ”${temp}\allwaves.dta”, replace

* append datasets with survey data
global waves 2008
foreach wave of global waves {
        use ”${temp}\main_‘wave’vr.dta”, clear
}

global waves 2009 2010 2011
foreach wave of global waves {
        append using ”${temp}\main_‘wave’vr.dta”
}

save ”${temp}\allwavesvr.dta”, replace

log close
```

## prepare_income_data.do

```
set more off
cap log close
log using ”${log}\log_prepare_income_data.log”, replace
*******************************************************************************************

* identify missing and imputed income values and set them to missing;
* map admin and survey income variables and derive annual amounts. NB: survey and admin
    var names are different;
* create a dataset with annual survey and admin amounts of earnings and benefits
*******************************************************************************************

* keep relevant vars in admin and survey datasets
* admin
use ”${temp}\allwaves.dta”, clear
keep pid hid pkzqu lnpkzqu rb06* jahr int1 hsize* bundesld region alter sex folge modus
    proxy p0000* r004* r005* r006* r000* p114* r009* p014000* p0120* uek_* *alog_* nh_*
    dlu_* h* p*
```

```
foreach var of varlist rb06*  int1 p0000* r004* r005* r006* r000* p114* r009* p0120* {
        rename `var' `var'_a
}
save "${temp}\data_allwaves_a.dta", replace

*survey
use "${temp}\allwavesvr.dta", clear
keep pid hid pkzqu lnpkzqu rb06* jahr hsize int1 bundesld region age sex folge modus
    proxy p0000* r004* r005* r006*  r000* p114* r009* p014000* p001000* p117000* p042*
    p045* p070* p040140* p040150* p037010* p037050* p029000* p030000* r009000 p0220*
    p0070*

foreach var of varlist rb06* hsize* int1 p0000* r004* r005* r006*  r000* p114* r009*
    p014000* p040140* p040150* p037010* p037050* p029000* p030000* {
        rename `var' `var'_s
}
save "${temp}\data_allwaves_s.dta", replace

* merge two datasets
set more off
use "${temp}\data_allwaves_a.dta", clear
merge m:m hid pid jahr using "${temp}\data_allwaves_s.dta"
drop if _merge!=3

*****************************************************************************************

* identify missing and imputed values and set them to missing
* admin income variables (`var'_3 = gross; `var'_3f = flag variable)
* flag values for income vars:
*       -2 n/a
*       -1 no answer and not yet imputed
*       0 no income and not imputed (only in admin data)
*       1 data record, not imputed
*       2 imputation based on income bracket
*       3 gross-to-net/net-to-gross imputation
*       4 logically deduced imputation
*       5 imputation based on longitudinal data
*       6 imputation based on cross-sectional data
*       7 value was later corrected
*       8 imputation based on a monthly income (only for vars of annual income)
*****************************************************************************************

* recode missing to zeros if no income and not imputed; set imputed values to missing
foreach var in uek alog nh dlu {
        recode `var'_3 (.=0) if `var'_3f == -2 | `var'_3f == 0 // if flag variable is -2
            (n/a) or 0 (no income, not imputed)
        replace `var'_3=. if `var'_3f != 1 & `var'_3f != -2 & `var'_3f != 0 // if flag
            variable takes any other value to 1, -2 and 0, i.e. value is imputed
}

foreach var in uek alog nh dlu {
        count if `var'_3 <0 & `var'_3!=.
}
* survey income variables (those ending with 3 and 4 refer to the gross and net values,
    respectively)
foreach var in p042003 p070014 p070024 p070044 {
        count if `var' <0 & `var'!=.
        * several observations with values equal to -2 or -1; set them to 0
        replace `var' = 0 if `var' == -2 | `var' == -1
}
foreach var in p042003 p070014 p070024 p070044 {
        recode `var' (.=0) if `var'f == -2 | `var'f == 0 // if flag variable is 1 (value
            according to data), -2 (n/a) or 0 (no income, not imputed)
        replace `var' = 0 if `var' == -2 & `var'f == -2
        replace `var' = . if `var'f != 1 & `var'f != -2 & `var'f !=0 // if flag variable
            takes any other value to 1, -2 and 0, i.e. value is imputed
}
* survey variables on number of months in earnings/benefit receipt - need to recode
    negative values to missing as variables are used to derive annual income amounts
foreach var in uek_2 alog_2 nh_2 dlu_2 p045002 p070012 p070022 p070042 {
        recode `var' (-1=.) // -1 means 'no answer and not imputed' - recode to missing
        recode `var' (-2=0) // -2 means n/a - recode to 0
```

```
}

// admin data: [name]_3 = gross amount; [name]_2 = duration of income receipt in months
// survey data: p[numbers]3 = gross amount; p[numbers]2 = duration of benefit receipt in
    months

* earnings of private employees (Unselbststaendigeneinkommen)
gen earnspr_g_s = p042003 // includes extra payments - 13th and 14th salaries
gen earnspr_g_a = uek_3 // includes extra payments - 13th and 14th salaries
gen earnspr_months_s = p045002
gen earnspr_months_a = uek_2
* unemployment insurance benefit (UI, Arbeitslosengeld)
gen ui_g_s = p070014*p070012
gen ui_g_a = alog_3
gen ui_months_s = p070012 // 1 observation has a value of 14
gen ui_months_a = alog_2
* unemployment assistance (UA, Notstandshilfe)
gen ua_g_s = p070024*p070022
gen ua_g_a = nh_3
gen ua_months_s = p070022
gen ua_months_a = nh_2
* assistance for covering living costs (ACLC, Beihilfe zur Deckung des Lebensunterhalts)
gen aclc_g_s = p070044*p070042
gen aclc_g_a = dlu_3
gen aclc_months_s = p070042
gen aclc_months_a = dlu_2

* labels for income variables
foreach var  in earnspr {
        label variable `var'_g_s "survey gross earnings"
        label variable `var'_g_a "admin gross earnings"
        label variable `var'_months_s "survey months of earnings"
        label variable `var'_months_a "admin months of earnings"
}
foreach var  in ui {
        label variable `var'_g_s "survey gross unemployment insurance"
        label variable `var'_g_a "admin gross unemployment insurance"
        label variable `var'_months_s "survey months of unemployment insurance"
        label variable `var'_months_a "admin months of unemployment insurance"
}
foreach var  in ua {
        label variable `var'_g_s "survey gross unemployment assistance"
        label variable `var'_g_a "admin gross unemployment assistance"
        label variable `var'_months_s "survey months of unemployment assistance"
        label variable `var'_months_a "admin months of unemployment assistance"
}
foreach var  in aclc {
        label variable `var'_g_s "survey gross assistance for living costs"
        label variable `var'_g_a "admin gross assistance for living costs"
        label variable `var'_months_s "survey months of assistance for living costs"
        label variable `var'_months_a "admin months of assistance for living costs"
}

save "${output}\annual_income_data.dta", replace

shell erase "${temp}\*.dta"
log close
```

## prepare_data_analysis.do

```
cap log close
log using "${log}\log_prepare_data_analysis.log", replace
*************************************************************************************
* prepare data set that will be used for the analysis
*************************************************************************************
set more off
use "${output}\annual_income_data.dta", clear

// IDs AND SURVEY CHARACTERISTICS
label var pid "Individual's id"
lab var hid "Household id"
rename jahr year
label variable year "year"
```

```
label variable folge "\hspace{0.1cm} wave"

label define yes_no 0 "no" 1 "yes" -1 "n/a"
forvalues i=1(1)12 { // 12 is the max number of people in a hh in the data
        gen temp`i' = pid if pkzqu == `i'
        recode temp`i' (.=0)
        bys year hid: egen ttemp`i' = sum(temp`i') // hh-level vars which contain each persons full id in the hh
        drop temp`i'
}
foreach var in p000020_a r004000_a r005000_a r006000_a { // vars for: person id that gave proxy interview; father id; mother id; partner id
        gen `var'2 = .
        forvalues i=1(1)12 {
                replace `var'2 = ttemp`i' if `var' == `i' // give full persons id if respective person in the hh matches short persons id
        }
}
drop ttemp*
rename p000020_a2 proxy_person
rename r004000_a2 idfather
rename r005000_a2 idmother
rename r006000_a2 idpartner
recode proxy_person idfather idmother idpartner (.=0)

gen proxy_interview = 1 if p000010_a==2 | age<17 // yes (everyone aged below 17 gets a proxy interview)
recode proxy_interview (.=0) // no
label variable proxy_interview "Proxy interview"
label values proxy_interview yes_no

gen proxy_partner = 1 if proxy_person==idpartner & idpartner!=0 & proxy_interview == 1 // proxy interview by partner
replace proxy_partner = 2 if (proxy_person!=idpartner | idpartner==0) & proxy_interview == 1 // proxy interview by someone else
replace proxy_partner = 0 if proxy_interview == 0 // personal interview, i.e. no proxy
label variable proxy_partner "Proxy partner"
label define label1 0 "no proxy" 1 "proxy by partner" 2 "proxy by someone else"
label values proxy_partner label1

gen int_month = hmonat // month of household interview
label variable int_month "Month of interview"
label define label2 1 "Jan" 2 "Feb" 3 "Mar" 4 "Apr" 5 "May" 6 "Jun" 7 "Jul" 8 "Aug" 9 "Sep" 10 "Oct" 11 "Nov" 12 "Dec"
label values int_month label2

gen int_type = modus // interview type: 1 = in person, 2 = by telephone
label variable int_type "Type of interview"
label define label3 1 "in person" 2 "by telephone"
label values int_type label3

gen int_same = int1_a // the same interviewer as last year: -3 dont know, -2 n/a, -1 missing, 1 yes, 2 no
replace int_same = 3 if int_same == -1
replace int_same = 4 if int_same == -2
replace int_same = 5 if int_same == -3
label variable int_same "Same interviewer as last year"
label define label4 1 "yes" 2 "no" 3 "missing" 4 "n/a" 5 "don't know"
label values int_same label4

bys pid: egen waves = sum([year>0])
lab var waves "Number of waves individual took part in the survey"

// INCOME
foreach var in earnspr ui ua aclc {
        gen ln`var'_a = ln(`var'_g_a) // log of admin income
        gen ln`var'_s = ln(`var'_g_s) // log of survey income
}
foreach var  in earnspr {
        label variable ln`var'_a "ln admin gross earnings"
        label variable ln`var'_s "ln survey gross earnings"
}
foreach var  in ui {
```

```
        label variable ln`var'_a "ln admin gross unemployment insurance"
        label variable ln`var'_s "ln survey gross unemployment insurance"
}
foreach var  in ua {
        label variable ln`var'_a "ln admin gross unemployment assistance"
        label variable ln`var'_s "ln survey gross unemployment assistance"
}
foreach var  in aclc {
        label variable ln`var'_a "ln admin gross assistance for living costs"
        label variable ln`var'_s "ln survey gross assistance for living costs"
}

foreach type in s a {
        gen uben_g_`type' = ui_g_`type' + ua_g_`type' + aclc_g_`type'
}
foreach var in uben {
        label variable `var'_g_s "survey gross unemployment benefits"
        label variable `var'_g_a "admin gross unemployment benefits"
}

gen dearnspr = earnspr_g_s - earnspr_g_a
lab var dearns "Gross survey minus gross admin earnings"

gen tearnspr_g_a = earnspr_g_a/1000 // divide earnings by 1000
gen tdearnspr = dearnspr/1000 // divide earnings difference by 1000

// SOCIO-ECONOMIC AND DEMOGRAPHIC CHARACTERISTICS
cap drop age
rename alter age
lab var age "Age"
label define reg 1 "Vienna" 2 "100,000+ residents" 3 "10,000-100,000 residents" 4 "less
    than 10,000 residents" 5 "missing"
replace region = 5 if region == .
label values region reg
lab var region "Region"
label variable sex "Sex"
replace sex = 0 if sex==1
replace sex = 1 if sex==2
label define sex 0 "male" 1 "female"
label values sex sex

gen incouple = ([idpartner>0]) // with a partner
label variable incouple "In a couple"
label values incouple yes_no

gen civil_serv = 1 if p014000_s >= 41 & p014000_s <= 47
replace civil_serv = 0 if civil_serv == .
replace civil_serv = 2 if p014000_s <0 & p014000_s != .
label variable civil_serv "Civil servant"
label define label5 1 "yes" 0 "no" 2 "n/a"
label values civil_serv label5

gen health = p102000 // health status: -1 missing, 1 very good, 2 good, 3 fair, 4 bad, 5
    very bad
replace health = 6 if health == -1 | health == .
label variable health "Self-reported health status"
label define label6 1 "very good" 2 "good" 3 "fair" 4 "bad" 5 "very bad" 6 "missing"
label values health label6

gen country_birth = p110000nu // country of origin: -1 missing, 1 Austria, 2 EU15/EFTA, 3
     new EU12, 4 former Yugoslavian countries (without Slovenia), 5 Turkey, 6 other
replace country_birth = p110000 if year == 2008 | year == 2009
replace country_birth = 7 if country_birth == -1 | country_birth == .
sort pid year
bys pid: gen temp_cb = country_birth[_N]
replace country_birth = temp_cb if country_birth == 7
drop temp_cb
label variable country_birth "Country of birth"
label define label7 7 "missing" 1 "Austria" 2 "EU15/EFTA" 3 "new EU12" 4 "former
    Yugoslavian countries (without Slovenia)" 5 "Turkey" 6 "other"
label values country_birth label7

gen paid_jobcourse = p040150_s
```

```
replace paid_jobcourse = 1 if p040150_s==1 | p040150_s==2
replace paid_jobcourse = 2 if p040150_s==3
replace paid_jobcourse = 3 if p040150_s==4
replace paid_jobcourse = 4 if p040150_s==5
replace paid_jobcourse = 5 if p040150_s==-1
replace paid_jobcourse = 6 if p040150_s==-2
replace paid_jobcourse = 7 if p040150_s==-3
replace paid_jobcourse = 0 if p040140_s==3 /*took_jobcourse == 0*/
label define p_jobcourse 0 "did not take" 1 "Mostly paid with own resources" 2 "Employer"
     3 "Labour market agency" 4 "Other institutions" 5 "missing" 6 "n/a" 7 "don't know"
lab values paid_jobcourse p_jobcourse

*********************************************************************
* highest_edu                    Highest Education achieved (following on  International
    Standard Classification of Education (ISCED) levels)
*
* 0: Not completed primary education
* 1: Primary (aged 6-9)
* 2: Lower secondary (Pflichtschule)
* 3: Upper Secondary: Lehre (Berufschule); Andere berufsbildende mittlere Schule; AHS (
    Allgeimeinbildende hoehere Schule) Oberstufe; berufsbildende mittlere Schule
* 4: Post Secondary: Krankenpflegeschule, Berufsbildende h here Schule - Normalform
* 5: Tertiary: Meister-, Werkmeisterausbildung; Berufsbildende h here Schule - Kolleg,
    Abiturientenlehrgang; Universit t, Akademie, Fachhochschule: Erstabschluss;
    Universit t: Doktoratsstudium als Zweitabschluss

* p118000 - highest education level; p117000 - current education level
*********************************************************************
gen highest_edu = p118000
recode highest_edu (-1=.) (0=0) (1=2) (2=3) (3=5) (4=4) (5=3) (6=3) (7=4) (8=5) (9=5)
    (10=5)
* replace missing values with info on current education
replace highest_edu = 0 if ((age<6 & p117000==.) | (p117000==1 | p117000==.)) & age<10 &
    highest_edu == . // not completed primary
replace highest_edu = 1 if (p117000==1 | (p117000==. & age<=15)) & age>=10 & highest_edu
    == . // current lower-secondary --> highest primary
replace highest_edu = 2 if (p117000==2 | p117000==5 | p117000==6) & highest_edu == . //
    current upper secondary --> highest lower secondary
replace highest_edu = 3 if (p117000==4 | p117000==7) & highest_edu == . // current post
    secondary --> highest upper secondary
replace highest_edu = 4 if (p117000==3 | p117000==8 | p117000==9 | p117000==10) &
    highest_edu == . // current tertiary --> highest post secondary

quietly count if !(highest_edu >= 0 & highest_edu <= 5)
display in y "No of observations with missing or invalid highest education achieved (
    highest_edu): " r(N)

assert (highest_edu >= 0 & highest_edu <= 5)
tab highest_edu, m
label variable highest_edu "Highest level of education"
label define label9 0 "Not completed primary education" 1 "Primary" 2 "Lower Secondary"
    3"Upper Secondary" 4"Post Secondary" 5"Tertiary"
label values highest_edu label9

gen edu = highest_edu
replace edu = 1 if highest_edu==0 | highest_edu==2 // low = not completed primary,
    primary or lower secondary
replace edu = 2 if highest_edu==3 | highest_edu==4 // middle = upper and post secondary
replace edu = 3 if highest_edu==5 // high = tertiary
label variable edu "Highest level of education"
label define high_edu 1 "\hspace{0.1cm} low" 2 "\hspace{0.1cm} middle" 3 "\hspace{0.1cm}
    high"
label values edu high_edu

*********************************************************************
* occup          Occupation
*  1: armed forces
*  2: senior officials and managers
*  3: professionals
*  4: technicians and associate professionals
*  5: clerks
*  6: service and saecon_state workers
*  7: skilled agricultural
```

```
*  8: craft and trades workers
*  9: plant and machine operators
*  10: elementary occups
*  11: not applicable (incl. children)
*  12: no answer
*********************************************************************
* current occupation, if main reported activity is working
gen occup_current  = 1 if p013000 == 1
replace occup_current = 2 if p013000 == 11 | p013000 == 12 | p013000 == 13
replace occup_current = 3 if p013000 == 21 | p013000 == 22 | p013000 == 23 | p013000 ==
    24
replace occup_current = 4 if p013000 == 31 | p013000 == 32 | p013000 == 33 | p013000 ==
    34
replace occup_current = 5 if p013000 == 41 | p013000 == 42
replace occup_current = 6 if p013000 == 51 | p013000 == 52
replace occup_current = 7 if p013000 == 61
replace occup_current = 8 if p013000 == 71 | p013000 == 72 | p013000 == 73 | p013000 ==
    74
replace occup_current = 9 if p013000 == 81 | p013000 == 82 | p013000 == 83
replace occup_current = 10 if p013000 == 91 | p013000 == 92 | p013000 == 93
replace occup_current = 11 if p013000 == -2 & occup_current == .
replace occup_current = 11 if age<=15
replace occup_current = 11 if lnpkzqu == . & occup_current  == .
replace occup_current = 12 if p013000 == -1 | occup_current == . // missing

qui count if !(occup_current >= 0 & occup_current <= 10)
if (r(N) > 0) noi display in y "No of observations with missing or invalid occup (occup)
    :" r(N)

tab occup_current, m
label variable occup_current "current occupation"
label define label10 1"armed forces" 2"senior officials and managers" 3"professionals"
    ///
        4"technicians and associate professionals" 5"clerks" 6"service and sales workers"
            7"skilled agricultural" ///
        8"craft and trades workers" 9"plant and machine operators" 10"elementary
           occupations" ///
        11"n/a" 12 "no answer"
label values occup_current label10

* last occupation, if main reported activity is not working
gen occup_last  = 1 if p007000 == 1
replace occup_last = 2 if p007000 == 11 | p007000 == 12 | p007000 == 13
replace occup_last = 3 if p007000 == 21 | p007000 == 22 | p007000 == 23 | p007000 == 24
replace occup_last = 4 if p007000 == 31 | p007000 == 32 | p007000 == 33 | p007000 == 34
replace occup_last = 5 if p007000 == 41 | p007000 == 42
replace occup_last = 6 if p007000 == 51 | p007000 == 52
replace occup_last = 7 if p007000 == 61
replace occup_last = 8 if p007000 == 71 | p007000 == 72 | p007000 == 73 | p007000 == 74
replace occup_last = 9 if p007000 == 81 | p007000 == 82 | p007000 == 83
replace occup_last = 10 if p007000 == 91 | p007000 == 92 | p007000 == 93
replace occup_last = 11 if p007000 == -2 & occup_last == .
replace occup_last = 11 if age<=15
replace occup_last = 11 if lnpkzqu == . & occup_last  == .
replace occup_last = 12 if occup_last == . // missing

qui count if !(occup_last >= 0 & occup_last <= 10)
if (r(N) > 0) noi display in y "No of observations with missing or invalid occup (occup)
    :" r(N)

tab occup_last, m
label variable occup_last "last occupation"
label define label10 1"armed forces" 2"senior officials and managers" 3"professionals"
    ///
        4"technicians and associate professionals" 5"clerks" 6"service and sales workers"
            7"skilled agricultural" ///
        8"craft and trades workers" 9"plant and machine operators" 10"elementary
           occupations" ///
        11"n/a" 12 "no answer", replace
label values occup_last label10

tab occup_last occup_current
gen occup = occup_current
```

```
replace occup = occup_last if occup_current ==. | occup_current ==11 | occup_current ==12
replace occup = 12 if occup == .
label variable occup "occupation"
label values occup label10

**********************************************************************
* industry       Industry
*  1 "agriculture"
*  2 "mining and quarrying"
*  3 "manufacturing"
*  4  "electricity , gas etc"
*  5 "water supply , sewage etc"
*  6 "construction"
*  7 "trade"
*  8 "transportation"
*  9 "accommodation and food"
*  10 "info and communication"
*  11 "finance"
*  12 "real estate"
*  13 "science"
*  14 "other professional etc"
*  15  "admin and support services"
*  16 "public admin., defence etc"
*  17 "education"
*  18 "health services"
*  19 "residential care and social work"
*  20 "arts , entertainment etc"
*  21 "other services"
*  22 "activities of hhs as employers"
*  22 "activities of hhs as employers"
*  23 "activities of extra-terr. organis."
*  24 "no answer"
*  25 "n/a"
**********************************************************************
* current industry , if main reported activity is working
gen industry_current = .
replace industry_current = 1 if p022000>=1 & p022000<5 & p022000!=. // Agriculture ,
    forestry and fishing
replace industry_current = 2 if p022000>=5 & p022000<10 & p022000!=. // Mining and
    quarrying
replace industry_current = 3 if p022000>=10 & p022000<35 & p022000!=. // Manufacturing
replace industry_current = 4 if p022000==35 // Electricity , Gas, Steam and Air
    Conditioning Supply
replace industry_current = 5 if p022000>=36 & p022000<40 & p022000!=. // Water supply ,
    sewerage , waste management and remediation
replace industry_current = 6 if p022000>=41 & p022000<45 & p022000!=. // Construction
replace industry_current = 7 if p022000>=45 & p022000<49 & p022000!=. // Whoecon_stateale
     and retail trade , repair of motor vehicecon_state and motorcycecon_state
replace industry_current = 8 if p022000>=49 & p022000<55 & p022000!=. // Transportation
    and storage
replace industry_current = 9 if p022000>=55 & p022000<58 & p022000!=. // Accommodation
    and food service activities
replace industry_current = 10 if p022000>=58 & p022000<68 & p022000!=. // Information and
     Communication
replace industry_current = 11 if p022000==68 & p022000!=. // Financial and insurance
    activities
replace industry_current = 12 if p022000>=69 & p022000<72 & p022000!=. // Real estate
    activities
replace industry_current = 13 if p022000==72 // Scientific research and development
replace industry_current = 14 if p022000>=73 & p022000<77 & p022000!=. // Other
    professional , scientific and technical activities
replace industry_current = 15 if p022000>=77 & p022000<84 & p022000!=. // Administrative
    and support service activities
replace industry_current = 16 if p022000==84 // Public administration and defence ,
    compulsory social security
replace industry_current = 17 if p022000==85 // Education
replace industry_current = 18 if p022000==86 // Human health services
replace industry_current = 19 if p022000>=87 & p022000<90 & p022000!=. // Residential
    care and social work activities
replace industry_current = 20 if p022000>=90 & p022000<94 & p022000!=. // Arts ,
    entertainment and recreation
replace industry_current = 21 if p022000>=94 & p022000<97 & p022000!=. // Other services
replace industry_current = 22 if p022000>=97 & p022000<99 & p022000!=. // Activities of
```

```
    households as employers; undifferentiated goods- and services-producing activities of
     households for own use
replace industry_current = 23 if p022000==99 // Activities of extra-territorial
    organisations and bodies
replace industry_current = 24 if p022000==-1 // didn't reply
replace industry_current = 25 if p022000==-2 // n/a as hasn't worked
label variable industry_current "current industry"
label define label11 1 "agriculture" 2 "mining and quarrying" 3 "manufacturing" ///
        4  "electricity, gas etc" 5 "water supply, sewage etc" 6 "construction" 7 "trade"
             8 "transportation" ///
        9 "accommodation and food" 10 "info and communication" 11 "finance"  ///
        12 "real estate" 13 "science" 14 "other professional etc" 15  "admin and support
            services" 16 "public admin., defence etc" ///
        17 "education" 18 "health services" 19 "residential care and social work" ///
        20 "arts, entertainment etc" 21 "other services" 22 "activities of hhs as
            employers" ///
        22 "activities of hhs as employers"  23 "activities of extra-terr. organis."  ///
        24 "no answer" 25 "n/a"
label values industry_current label11

* last industry, if main reported activity is not working
gen industry_last = .
replace industry_last = 1 if p012010_a>=1 & p012010_a<5 & p012010_a!=. // Agriculture,
    forestry and fishing
replace industry_last = 2 if p012010_a>=5 & p012010_a<10 & p012010_a!=. // Mining and
    quarrying
replace industry_last = 3 if p012010_a>=10 & p012010_a<35 & p012010_a!=. // Manufacturing
replace industry_last = 4 if p012010_a==35 // Electricity, Gas, Steam and Air
    Conditioning Supply
replace industry_last = 5 if p012010_a>=36 & p012010_a<40 & p012010_a!=. // Water supply,
     sewerage, waste management and remediation
replace industry_last = 6 if p012010_a>=41 & p012010_a<45 & p012010_a!=. // Construction
replace industry_last = 7 if p012010_a>=45 & p012010_a<49 & p012010_a!=. //
    Whoecon_stateale and retail trade, repair of motor vehicecon_state and
    motorcycecon_state
replace industry_last = 8 if p012010_a>=49 & p012010_a<55 & p012010_a!=. //
    Transportation and storage
replace industry_last = 9 if p012010_a>=55 & p012010_a<58 & p012010_a!=. // Accommodation
     and food service activities
replace industry_last = 10 if p012010_a>=58 & p012010_a<68 & p012010_a!=. // Information
    and Communication
replace industry_last = 11 if p012010_a==68 & p012010_a!=. // Financial and insurance
    activities
replace industry_last = 12 if p012010_a>=69 & p012010_a<72 & p012010_a!=. // Real estate
    activities
replace industry_last = 13 if p012010_a==72 // Scientific research and development
replace industry_last = 14 if p012010_a>=73 & p012010_a<77 & p012010_a!=. // Other
    professional, scientific and technical activities
replace industry_last = 15 if p012010_a>=77 & p012010_a<84 & p012010_a!=. //
    Administrative and support service activities
replace industry_last = 16 if p012010_a==84 // Public administration and defence,
    compulsory social security
replace industry_last = 17 if p012010_a==85 // Education
replace industry_last = 18 if p012010_a==86 // Human health services
replace industry_last = 19 if p012010_a>=87 & p012010_a<90 & p012010_a!=. // Residential
    care and social work activities
replace industry_last = 20 if p012010_a>=90 & p012010_a<94 & p012010_a!=. // Arts,
    entertainment and recreation
replace industry_last = 21 if p012010_a>=94 & p012010_a<97 & p012010_a!=. // Other
    services
replace industry_last = 22 if p012010_a>=97 & p012010_a<99 & p012010_a!=. // Activities
    of households as employers; undifferentiated goods- and services-producing activities
     of households for own use
replace industry_last = 23 if p012010_a==99 // Activities of extra-territorial
    organisations and bodies
replace industry_last = 24 if p012010_a==-1 // didn't reply
replace industry_last = 25 if p012010_a==-2 // n/a as hasn't worked
label variable industry_last "last industry"
label define label11 1 "agriculture" 2 "mining and quarrying" 3 "manufacturing" ///
        4  "electricity, gas etc" 5 "water supply, sewage etc" 6 "construction" 7 "trade"
             8 "transportation" ///
        9 "accommodation and food" 10 "info and communication" 11 "finance"  ///
        12 "real estate" 13 "science" 14 "other professional etc" 15  "admin and support
```

```
            services" 16 "public admin., defence etc" ///
        17 "education" 18 "health services" 19 "residential care and social work" ///
        20 "arts, entertainment etc" 21 "other services" 22 "activities of hhs as
            employers" ///
        22 "activities of hhs as employers"  23 "activities of extra-terr. organis."  ///
        24 "no answer" 25 "n/a", replace
label values industry_last label11

tab industry_last industry_current
gen industry = industry_current
replace industry = industry_last if industry_current ==. | industry_current ==24 |
    industry_current ==25
replace industry = 24 if industry == .
label variable industry "industry"
label values industry label11

* n adults and children in the hh
gen member=1
sort year hid
gen child=1 if age<16
gen adult=1 if age>=16
recode child adult (.=0)
bys year hid: egen nchild=sum(child)
bys year hid: egen nadult=sum(adult)
gen nch = nchild
replace nch = 3 if nchild>=3
label variable nch "Number of children in the household (aged <16)"

gen nad = nadult
replace nad = 3 if nadult>=3
label variable nad "Number of adults in the household (aged 16+)"

*age groups
gen age_gr = .
replace age_gr = 1 if age>=0 & age<5
replace age_gr = 2 if age>=5 & age<10
replace age_gr = 3 if age>=10 & age<15
replace age_gr = 4 if age>=15 & age<20
replace age_gr = 5 if age>=20 & age<25
replace age_gr = 6 if age>=25 & age<30
replace age_gr = 7 if age>=30 & age<35
replace age_gr = 8 if age>=35 & age<40
replace age_gr = 9 if age>=40 & age<45
replace age_gr = 10 if age>=45 & age<50
replace age_gr = 11 if age>=50 & age<55
replace age_gr = 12 if age>=55 & age<60
replace age_gr = 13 if age>=60 & age<65
replace age_gr = 14 if age>=65 & age<70
replace age_gr = 15 if age>=70 & age<75
replace age_gr = 16 if age>=75
label variable age_gr "Age group (in 5-years bands)"
label define label12 1 "0-4" 2 "5-9" 3 "10-14" 4 "16-19" 5 "20-24" 6 "25-29" 7 "30-34" 8
    "35-39" 9 "40-44" 10 "45-49" ///
11 "50-54" 12 "55-59" 13 "60-64" 14 "65-69" 15 "70-74" 16 "75+"
label values age_gr label12

**********************************************************************
* counting main activities in calendar year
* 1 = full-time employed
* 2 = part-time employed
* 3 = full-time employer/self-employed
* 4 = part-time employer/self-employed
* 5 = unemployed
* 6 = retired
* 7 = student
* 8 = doing house-work or having caring responsibilities
* 9 = civil service (e.g. military)
* 10 = disabled
* 11 = not active due to other reasons
**********************************************************************
*gen ftime_emp = 0
forvalues x = 1 /11 { // calculating number of months spent by activity
    egen act`x' = anycount(p040010 p040020 p040030 p040040 p040050 p040060 p040070
```

```
        p040080 p040090 p040100 p040110 p040120), values(`x')
}

gen act_ftime_emp=(act1==12) // full-year full-time employed
gen act_ptime_emp=(act2==12) // full-year part-time employed
gen act_unemp=(act5==12) // full-year unemployed

gen act_ftime_emp_months = act1 // n months full-time employed
gen act_unemp_months = act5 // n months unemployed

foreach type in s a {
        gen earner_`type' = 1 if earnspr_g_`type'!=. & earnspr_g_`type'>0
        replace earner_`type' = 0 if earnspr_g_`type' == 0
        label values earner_`type' yes_no
}
label variable earner_a "Earner (admin)"
label variable earner_s "Earner (survey)"

// DEFINING INDICATORS FOR RECEIPT AND REPORTING TYPE (FALSE +/- & TRUE +/-)
label define receipt 0 "no" 1 "yes" -1 "missing/imputed"
* yes/no indicators for admin/survey receipt
foreach var in earnspr uben ui ua aclc { // for each income type
        gen `var'_reca = 1 if `var'_g_a!=0 & `var'_g_a!=. // income recipient according
            to the admin data
        replace `var'_reca = 0 if `var'_g_a==0 // non-recipient according to the admin
            data
        label values `var'_reca receipt
        label variable `var'_reca "Admin receipt"

        gen `var'_recs = 1 if `var'_g_s!=0 & `var'_g_s!=. // income recipient according
            to the survey data
        replace `var'_recs = 0 if `var'_g_s==0 // non-recipient according to the survey
            data
        label values `var'_recs receipt
        label variable `var'_recs "Survey receipt"
}

foreach var of varlist *_reca *_recs {
        recode `var' (.=-1) // recode missing/imputed values to -1
}

* reporting type
label define false 1 "false +" 2 "false -" 3 "true -" 4 "true +" 5 "missing/imputed"
foreach var in earnspr uben ui ua aclc {
        gen `var'_false = 1 if `var'_recs == 1 & `var'_reca==0 // false +, i.e. non-zero
            in survey and zero in admin
        replace `var'_false = 2 if `var'_recs == 0 & `var'_reca==1 // false -, i.e. zero
            in survey and non-zero in admin
        replace `var'_false = 3 if `var'_recs==0 & `var'_reca==0 // true -, i.e. zero in
            both admin and survey
        replace `var'_false = 4 if `var'_recs==1 & `var'_reca==1 // true +, i.e. non-zero
             in both admin and survey
        replace `var'_false = 5 if `var'_false==. // admin and/or survey recipt is
            missing/imputed
        label values `var'_false false
        label variable `var'_false "Reporting categories: false+/-, true+/-"
}

* indicator for receiving income according to both survey and admin (true + = 1) vs only
    in the survey (false + = 0)
label define not_rep 1 "true +" 0 "false -" -1 "false +/true -"
foreach var in earnspr uben ui ua aclc {
        gen `var'_reportrec = ([`var'_false!=2]) // not false -
        replace `var'_reportrec = -1 if `var'_false==1 | `var'_false==3 | `var'_false==5
            // 1=false +, 3=0 survey and admin amounts, 5=admin or survey value imputed
        label variable `var'_reportrec "true +"
        label values `var'_reportrec not_rep
}

sort pid folge
keep hid pid folge year *waves* sex *earn* *ub* *ui* *ua* *aclc* proxy_partner id* int_*
    incouple civil_serv health country_birth *edu* occup industry nch nad age* *emp* act*
     region *jobc*
```

```
save "${analysis_input}\data_analysis.dta", replace
log close
```

# Analysis

## master_analysis.do

```
*************************************************************************************
* PROJECT:              Income Source Confusion using the SILC
* AUTHORS:                              Christopher R. Bollinger and Iva V. Tasseva
* DESCRIPTION:          master do-file for analysis
*************************************************************************************
clear all
clear matrix
set more off
set mem 32g
set type double
macro drop _all
set matsize 11000

* define locations // TO DO
global main = ""    // main Analysis folder
// subfolders
global input ""     // data for analysis

global do ""        // do-files
global log ""       // log-files
global tabls ""     // tables

set more off

* Sample characteristics
do "${do}\sample_characteristics.do" // Table 1

* Misreporting of unemployment benefits and earnings
do "${do}\misreport_benefits_earnings.do" // Table 2

* Combinations of income receipt, given admin receipt
do "${do}\combinations_receipt.do" // Table 3

* OLS regressions for the probability of reporting the income in the survey, conditional
    on receiving it in the admin
do "${do}\reg_report_receipt.do" // Tables 4, 5, S1, S3, S5 and S6

* Mean survey and admin amounts by benefits true +, false - and false +
do "${do}\mean_amounts.do" // Table 6

* The mean of the error in earnings (survey-admin earnings) for those who overreport
    earnings and receive benefits in t while not receiving benefits in s
do "${do}\error_earnings.do" // Table 7

* OLS regression for the error in earnings (survey-admin earnings) on the reverse error
    in benefits (admin-survey sum of benefits)
do "${do}\reg_error_earnings_on_ben.do" // Table 8

* Benefit recipient status by education
do "${do}\recipient_status_by_education.do" // Table 9

* Bias in the returns to education
do "${do}\application_bias_returns_education.do" // Tables 10 and 11

* Bias in the returns to job training
do "${do}\application_bias_returns_training.do" // Tables 12, 13, S7 and S8

* Logit regressions for the probability of reporting the income in the survey,
    conditional on receiving it in the admin
do "${do}\logit_report_receipt.do" // Tables S2 and S4

* tables_labels.do called by:
*       - reg_report_receipt.do
*       - reg_error_earnings_on_ben.do
*       - application_bias_returns_education
*       - application_bias_returns_training
```

```
*       - logit_report_receipt.do
```

## sample_characteristics.do

```
set more off
cap log close
est clear
log using "${log}\log_sample.log", replace
************************************************************************************
* Table 1: Sample benacteristics: SILC 2008-2011
*************************************************************************************
set more off
use "${input}\data_analysis.dta", clear

keep if age>=16
gen one = 1

* individuals' characteristics based on survey information
gen fyear_ftime_emp = ([earnspr_months_s==12 & act_ftime_emp==1 & earnspr_g_s!=0 &
    earnspr_g_s!=. ]) // full-time full-year employed

foreach var in ui ua aclc { // for each unemployment benefit
        bys pid: egen `var'_multipley = sum([`var'_recs == 1]) // received benefit for 1
            wave
        replace `var'_multipley = 2 if `var'_multipley > 1 // received benefit over
            multiple waves
}

* table columns - all, men, women
global all one == 1
global man sex == 0
global woman sex == 1

* make table
mat tab_sample = J(38,3,.z) // table 38 x 3
local row = 1
local col = 1
foreach var in all man woman { // for each column
        local row = 1

        global cond ${`var'} // for all or conditional on being a man or a woman

        * mean age
        tabstat age if ${cond}, stat(mean) save
        matrix tots=r(StatTotal)
        mat tab_sample[`row', `col'] = tots[1,1]
        local row=`row'+1

        * % by education (3 levels)
        tabstat one if ${cond}, stat(n) by(edu) save
        matrix tots=r(StatTotal)
        forvalues r=1(1)3 {
                matrix tot`r'=r(Stat`r')
                mat tab_sample[`row', `col'] = tot`r'[1,1] / tots[1,1] * 100
                local row=`row'+1
        }

        * % with partner
        tabstat one if ${cond}, stat(n) by(incouple) save
        matrix tots=r(StatTotal)
        matrix totc=r(Stat2)
        mat tab_sample[`row', `col'] = totc[1,1] / tots[1,1] * 100
        local row=`row'+1

        * % by country of birth (3 values)
        tabstat one if ${cond}, stat(n) by(country_birth) save
        matrix tots=r(StatTotal)
        forvalues r=1(1)7 {
                matrix tot`r'=r(Stat`r')
        }
        foreach r in 1 {
                mat tab_sample[`row', `col'] = tot`r'[1,1] / tots[1,1] * 100 // Austria
                local row=`row'+1
```

```
}
mat tab_sample[`row', `col'] = (tot2[1,1] + tot3[1,1]) / tots[1,1] * 100 // EU-27
local row=`row'+1
mat tab_sample[`row', `col'] = (tot4[1,1] + tot5[1,1] + tot6[1,1] + tot7[1,1]) /
    tots[1,1] * 100 // Other + missing
local row=`row'+1

* % full-time full-year employed, % recipient of earnings
foreach ben in fyear_ftime_emp earnspr_recs {
        tabstat one if ${cond} & `ben'!=-1, stat(n) by(`ben') save
        matrix tots=r(StatTotal)
        matrix totc=r(Stat2)
        mat tab_sample[`row', `col'] = totc[1,1] / tots[1,1] * 100
        local row=`row'+1
}

* % by proxy (3 values)
tabstat one if ${cond}, stat(n) by(proxy_partner) save
matrix tots=r(StatTotal)
forvalues r=1(1)3 {
        matrix tot`r'=r(Stat`r')
        mat tab_sample[`row', `col'] = tot`r'[1,1] / tots[1,1] * 100
        local row=`row'+1
}

* % by job course (6 values)
tabstat one if ${cond}, stat(n) by(paid_jobcourse) save
matrix tots=r(StatTotal)
forvalues r=1(1)7 {
        matrix tot`r'=r(Stat`r')
}
forvalues r=1(1)5 {
        mat tab_sample[`row', `col'] = tot`r'[1,1] / tots[1,1] * 100
        local row=`row'+1
}
mat tab_sample[`row', `col'] = (tot6[1,1] + tot7[1,1]) / tots[1,1] * 100 //
    missing + n/a
local row=`row'+1

* % recipient of UI (x5)
foreach ben in ui {
        tabstat one if ${cond}, stat(n) by(`ben'_recs) save
        matrix tots=r(StatTotal)
        forvalues r=1(1)3 {
                matrix tot`r'=r(Stat`r')
                mat tab_sample[`row', `col'] = tot`r'[1,1] / tots[1,1] * 100
                local row=`row'+1
        }

        sort pid
        tabstat one if ${cond} & pid[_n]!=pid[_n+1], stat(n) by(`ben'_multipley)
            save
        forvalues r=2(1)3 {
                matrix tot`r'=r(Stat`r')
        }
        mat tab_sample[`row', `col'] = tot2[1,1] / (tot2[1,1] + tot3[1,1]) * 100
            // recipient in 1 year
        local row=`row'+1
        mat tab_sample[`row', `col'] = tot3[1,1] / (tot2[1,1] + tot3[1,1]) * 100
            // recipient over multiple years
        local row=`row'+1
}

* % recipient of UA (x4)
foreach ben in ua {
        tabstat one if ${cond} & `ben'_recs!=-1, stat(n) by(`ben'_recs) save //
            exclude missing/ imputed as too few cases to disclose
        matrix tots=r(StatTotal)
        forvalues r=1(1)2 {
                matrix tot`r'=r(Stat`r')
                mat tab_sample[`row', `col'] = tot`r'[1,1] / tots[1,1] * 100
                local row=`row'+1
        }
```

```
                sort pid
                tabstat one if ${cond} & pid[_n]!=pid[_n+1], stat(n) by(`ben'_multipley)
                    save
                forvalues r=2(1)3 {
                        matrix tot`r'=r(Stat`r')
                }
                mat tab_sample[`row', `col'] = tot2[1,1] / (tot2[1,1] + tot3[1,1]) * 100
                    // recipient in 1 year
                local row=`row'+1
                mat tab_sample[`row', `col'] = tot3[1,1] / (tot2[1,1] + tot3[1,1]) * 100
                    // recipient over multiple years
                local row=`row'+1
        }

        * % recipient of ACLC (x4)
        foreach ben in aclc {
                tabstat one if ${cond}, stat(n) by(`ben'_recs) save
                matrix tots=r(StatTotal)
                forvalues r=1(1)3 {
                        matrix tot`r'=r(Stat`r')
                }
                forvalues r=1(1)3 {
                        mat tab_sample[`row', `col'] = tot`r'[1,1] / tots[1,1] * 100
                        if (tot1[1,1] <20 | tot2[1,1] <20 | tot3[1,1] <20) mat tab_sample
                            [`row', `col'] = . // set to missing if too few closes to
                            disclose
                        local row=`row'+1
                }

                sort pid
                tabstat one if ${cond} & pid[_n]!=pid[_n+1], stat(n) by(`ben'_multipley)
                    save
                forvalues r=2(1)3 {
                        matrix tot`r'=r(Stat`r')
                }
                mat tab_sample[`row', `col'] = tot2[1,1] / (tot2[1,1] + tot3[1,1]) * 100
                if (tot2[1,1] <20 | tot3[1,1] <20) mat tab_sample[`row', `col'] = . //
                    set to missing if too few closes to disclose
                local row=`row'+1
                mat tab_sample[`row', `col'] = tot3[1,1] / (tot2[1,1] + tot3[1,1]) * 100
                if (tot2[1,1] <20 | tot3[1,1] <20) mat tab_sample[`row', `col'] = . //
                    set to missing if too few closes to disclose
                local row=`row'+1
        }

        * by n wave: 1, 2, 3 or 4 waves
        tabstat one if ${cond}, stat(n) by(waves) save
        forvalues r=1(1)4 {
                matrix tot`r'=r(Stat`r')
                mat tab_sample[`row', `col'] = tot`r'[1,1]
                local row=`row'+1
        }

        * all observations
        tabstat one if ${cond}, stat(n) save
        matrix tots=r(StatTotal)
        mat tab_sample[`row', `col'] = tots[1,1]
        local row=`row'+1

        local col=`col'+1 // move on to the next column
}

estadd matrix tab_sample = tab_sample, replace
matrix list tab_sample, nodotz

global lab r1 "Age" ///
        r2 "Education: \\ \hspace{0.2cm} Low" ///
        r3 "\hspace{0.2cm} Middle" ///
        r4 "\hspace{0.2cm} High" ///
        r5 "With partner" ///
        r6 "Country of birth: \\ \hspace{0.2cm} Austria" ///
        r7 "\hspace{0.2cm} EU-27" ///
```

```
        r8 "\hspace{0.2cm} Other" ///
        r9 "Full-time, full-year employed" ///
        r10 "With survey earnings" ///
        r11 "Proxy interview: \\ \hspace{0.2cm} No" ///
        r12 "\hspace{0.2cm} Partner" ///
        r13 "\hspace{0.2cm} Someone else" ///
        r14 "Job training: \\ \hspace{0.2cm} Did not take" ///
        r15 "\hspace{0.2cm} Mostly paid with own resources" ///
        r16 "\hspace{0.2cm} Employer" ///
        r17 "\hspace{0.2cm} Labour market agency" ///
        r18 "\hspace{0.2cm} Other institutions" ///
        r19 "\hspace{0.2cm} Missing/n/a" ///
        r20 "UI in the survey: \\ \hspace{0.2cm} Missing/imputed" ///
        r21 "\hspace{0.2cm} No receipt" ///
        r22 "\hspace{0.2cm} With receipt:" ///
        r23 "\hspace{0.5cm} \textit{1 year only}" ///
        r24 "\hspace{0.5cm} \textit{Multiple years}" ///
        r25 "UA in the survey: \\ \hspace{0.2cm} No receipt" ///
        r26 "\hspace{0.2cm} With receipt:" ///
        r27 "\hspace{0.5cm} \textit{1 year only}" ///
        r28 "\hspace{0.5cm} \textit{Multiple years}" ///
        r29 "ACLC in the survey: \\ \hspace{0.2cm} Missing/imputed" ///
        r30 "\hspace{0.2cm} No receipt" ///
        r31 "\hspace{0.2cm} With receipt:" ///
        r32 "\hspace{0.5cm} \textit{1 year only}" ///
        r33 "\hspace{0.5cm} \textit{Multiple years}" ///
        r34 "Observations: \\ \hspace{0.2cm} 1 wave only" ///
        r35 "\hspace{0.2cm} 2 waves" ///
        r36 "\hspace{0.2cm} 3 waves" ///
        r37 "\hspace{0.2cm} 4 waves" ///
        r38 "All observations"

estout e(tab_sample, fmt("%15.1fc %15.1fc %15.1fc %15.1fc %15.1fc %15.1fc %15.1fc %15.1fc
     %15.1fc %15.1fc %15.1fc %15.1fc %15.1fc %15.1fc %15.1fc %15.1fc %15.1fc %15.1fc
    %15.1fc %15.1fc %15.1fc %15.1fc %15.1fc %15.1fc %15.1fc %15.1fc %15.1fc %15.1fc %15.1
    fc %15.1fc %15.1fc %15.1fc %15.1fc %15.0fc" ) ) ///
        using "${tabls}\tabl_sample.tex", ///
        style(tex) abbrev wrap mgroups(none) mlabels(none) ///
        label nolz varwidth(110) modelwidth(10) collabels(none) ///
        varlabels(${lab}) ///
        noomitted replace
```

## misreport_benefits_earnings.do

```
set more off
cap log close
est clear
log using "${log}\log_misreport_benefits_earnings.log", replace
****************************************************************************************************
* Table 2: Misreporting of unemployment benefits and earnings
****************************************************************************************************

set more off
use "${input}\data_analysis.dta", clear
keep if age>=16

*** false negative and a) false positive - number of cases and in %
foreach var in ui ua aclc uben earnspr {
        egen n`var'_tr = sum([`var'_reca == 1 & `var'_recs != -1]) // n true (admin)
            recipients
        egen n`var'_tnr = sum([`var'_reca == 0 & `var'_recs != -1]) // n true (admin) non
            -recipients
        egen n`var'_fra = sum([`var'_false == 1]) // n false +
        egen n`var'_fnr = sum([`var'_false == 2]) // n false -

        * false negatives in %
        gen fn_`var' = n`var'_fnr/n`var'_tr*100

        * a) false positives in %
        gen fpa_`var' = n`var'_fra/n`var'_tnr*100
}
```

```
*** b) false positive
* identify those who are on at least one of two programmes according to the admin data
foreach var1 in ui {
        foreach var2 in ua aclc {
                gen `var1'`var2'_reca = 1 if (`var1'_reca==1 & `var1'_recs != -1) | (`
                    var2'_reca==1 & `var2'_recs != -1)
        }
}
foreach var1 in ua {
        foreach var2 in aclc {
                gen `var1'`var2'_reca = 1 if (`var1'_reca==1 & `var1'_recs != -1) | (`
                    var2'_reca==1 & `var2'_recs != -1)
        }
}

* calculate b) false positive - number of cases and in %
* - ACLC
foreach var1 in uiua {
        foreach var2 in aclc {
                egen n`var1'_tr = sum(`var1'_reca) if `var1'_reca==1 & `var2'_reca==0 & `
                    var2'_recs!=-1 // admin recipient of benefit 1 or 2 and not admin
                    recipient of benefit 3
                egen n`var2'_frb = sum(`var2'_recs) if `var1'_reca==1 & `var2'_reca==0 &
                    `var2'_recs==1 // admin recipient of benefit 1 or 2 and falsely
                    reporting receipt of benefit 3
                gen fpb_`var2' = n`var2'_frb / n`var1'_tr*100
        }
}
* - UA
foreach var1 in uiaclc {
        foreach var2 in ua {
                egen n`var1'_tr = sum(`var1'_reca) if `var1'_reca==1 & `var2'_reca==0 & `
                    var2'_recs!=-1 // admin recipient of benefit 1 or 2 and not admin
                    recipient of benefit 3
                egen n`var2'_frb = sum(`var2'_recs) if `var1'_reca==1 & `var2'_reca==0 &
                    `var2'_recs==1 // admin recipient of benefit 1 or 2 and falsely
                    reporting receipt of benefit 3
                gen fpb_`var2' = n`var2'_frb / n`var1'_tr*100
        }
}
* - UI
foreach var1 in uaaclc {
        foreach var2 in ui {
                egen n`var1'_tr = sum(`var1'_reca) if `var1'_reca==1 & `var2'_reca==0 & `
                    var2'_recs!=-1 // admin recipient of benefit 1 or 2 and not admin
                    recipient of benefit 3
                egen n`var2'_frb = sum(`var2'_recs) if `var1'_reca==1 & `var2'_reca==0 &
                    `var2'_recs==1 // admin recipient of benefit 1 or 2 and falsely
                    reporting receipt of benefit 3
                gen fpb_`var2' = n`var2'_frb / n`var1'_tr*100
        }
}

* calculate proportion of respondents making an error (survey - admin amount)
foreach sample in truep all {
        foreach var in ui ua aclc uben earnspr {
                global truep `var'_false == 4 // conditional on sample of true positive
                global all (`var'_false == 1 | `var'_false == 2 | `var'_false == 4) //
                    conditional on sample of all (true +/false +/false -)

                gen error_`var'_`sample' = `var'_g_s - `var'_g_a if ${`sample'} // error
                    (survey - admin amount)
                sum error_`var'_`sample'
                gen sderror_`var'_`sample' = r(sd) // sd of the error
                gen nerror = r(N) // number observations

                * error of >-10%/<10% of the admin amount
                gen temp_error = 1 if abs(error_`var'_`sample'/`var'_g_a) >= 0 & abs(
                    error_`var'_`sample'/`var'_g_a) < 0.1 & abs(error_`var'_`sample'/`var
                    '_g_a)!=. & error_`var'_`sample'!=. & ${`sample'}
                sum temp_error
                gen ntemp_error = r(N)
                gen serror10_`var'_`sample' = ntemp_error / nerror * 100 // proportion
```

```
                with an error
            gen nerror10_`var'_`sample' = ntemp_error // n with an error
            cap drop *temp_error*

            * error of 10-50%
            gen temp_error = 1 if (error_`var'_`sample'/`var'_g_a) >= 0.1 & (error_`
                var'_`sample'/`var'_g_a) < 0.5 & (error_`var'_`sample'/`var'_g_a)!=.
                & error_`var'_`sample'!=. & ${`sample'}
            sum temp_error
            gen ntemp_error = r(N)
            gen serror1050_`var'_`sample' = ntemp_error / nerror * 100 // proportion
                with an error
            gen nerror1050_`var'_`sample' = ntemp_error
            cap drop *temp_error*

            * error of 50%+ (or undefined if false +)
            gen temp_error = 1 if (((error_`var'_`sample'/`var'_g_a) >= 0.5 & (error_
                `var'_`sample'/`var'_g_a)!=.) | (`var'_g_s!=0 & `var'_g_a==0)) &
                error_`var'_`sample'!=. & ${`sample'}
            sum temp_error
            gen ntemp_error = r(N)
            gen serror50_`var'_`sample' = ntemp_error / nerror * 100 // proportion
                with an error
            gen nerror50_`var'_`sample' = ntemp_error
            cap drop *temp_error*

            * error of -10% to-50%
            gen temp_error = 1 if (error_`var'_`sample'/`var'_g_a) <= -0.1 & (error_`
                var'_`sample'/`var'_g_a) > -0.5 & (error_`var'_`sample'/`var'_g_a)!=.
                 & error_`var'_`sample'!=. & ${`sample'}
            sum temp_error
            gen ntemp_error = r(N)
            gen serrorm1050_`var'_`sample' = ntemp_error / nerror * 100 // proportion
                 with an error
            gen nerrorm1050_`var'_`sample' = ntemp_error
            cap drop *temp_error*

            * error of <=-50%
            gen temp_error = 1 if (error_`var'_`sample'/`var'_g_a) <= -0.5 & (error_`
                var'_`sample'/`var'_g_a)!=. & error_`var'_`sample'!=. & ${`sample'}
            sum temp_error
            gen ntemp_error = r(N)
            gen serrorm50_`var'_`sample' = ntemp_error / nerror * 100 // proportion
                with an error
            gen nerrorm50_`var'_`sample' = ntemp_error
            cap drop nerror *temp_error*

            foreach val in 10 1050 50 m1050 m50 {
                    replace serror`val'_`var'_`sample' = . if nerror`val'_`var'_`
                        sample' < 20 // do not disclose cells with too few
                        observations
            }
    }

    * correlation of admin and survey amounts
    foreach var in ui ua aclc uben earnspr {
            global truep `var'_false == 4
            global all (`var'_false == 1 | `var'_false == 2 | `var'_false == 4)
            corr `var'_g_a `var'_g_s if ${`sample'}
            gen corr_`var'_`sample' = r(rho)
    }
}

*** make table
gen fpb_uben = . // for the total --> no b) false + by definition
gen nuben_frb = .
gen fpb_earnspr = . // for earnings --> no b) false + by definition
gen nearnspr_frb = .

mat misreport = J(26,5,.z) // table
local row = 1
local col = 1
foreach var in ui ua aclc uben earnspr { // each column corresponds to an income source
```

```
        local row = 1 // go back to first row

        * false - (%), false - (n), a) false + (%), a) false + (n), b) false + (%), b)
            false + (n)
        tabstat fn_`var' n`var'_fnr fpa_`var' n`var'_fra fpb_`var' n`var'_frb, stat(mean)
             form(%15.2fc %15.0fc %15.2fc %15.0fc %15.2fc %15.0fc) save
        matrix tots=r(StatTotal)
        local row=`row'
        forvalues r=1(1)6 { // rows 1-6
                mat misreport[`row', `col'] = tots[1,`r']
                local row=`row'+1 // move on to the next row
        }

        * error in the amounts conditional on sample of true + or all (true +/false +/
            false -)
        foreach sample in truep all {
                global truep `var'_false == 4
                global all (`var'_false == 1 | `var'_false == 2 | `var'_false == 4)

                global cond ${`sample'}
                * ...mean admin amount, mean survey amount, % with an error of <=-50%,
                    -50% to -10%, -10% to 10%, 10% to 50%, >=50%, std deviation of the
                    error, correlation admin and survey amount, number observations
                tabstat `var'_g_a `var'_g_s serrorm50_`var'_`sample' serrorm1050_`var'_`
                    sample' serror10_`var'_`sample' serror1050_`var'_`sample' serror50_`
                    var'_`sample' sderror_`var'_`sample' corr_`var'_`sample' if $cond,
                    stat(mean n) save
                matrix tots=r(StatTotal)
                forvalues r=1(1)9 {
                        mat misreport[`row', `col'] = tots[1,`r']
                        local row=`row'+1 // move on to the next row
                }
                mat misreport[`row', `col'] = tots[2,1]
                local row=`row'+1 // move on to the next row
        } // end of sample loop
        local col=`col'+1 // move to the next column, i.e. next income source
} // end of column loop

estadd matrix misreport = misreport, replace
matrix list misreport, nodotz

global lab r1 "\multirow{2}{*}{False —} & \%" ///
        r2 "& Obs." ///
        r3 "\multirow{2}{*}{a) False +} & \%" ///
        r4 "& Obs." ///
        r5 "\multirow{2}{*}{b) False +} & \%" ///
        r6 "& Obs." ///
        r7 "\hline \textbf{\textit{True +}} \\ \hspace{0.1cm} Mean administrative amount
            &" ///
        r8 "\hspace{0.1cm} Mean survey amount &" ///
        r9 "\hspace{0.1cm} Error in \% of administrative amount: \\ \hspace{0.3cm} $\leq$
            —50\% & " ///
        r10 "\hspace{0.3cm} $>$—50\% \& $\leq$—10\% & " ///
        r11 "\hspace{0.3cm} $>$—10\% \& $<$10\% & " ///
        r12 "\hspace{0.3cm} $\geq$10\% \& $<$50\% & " ///
        r13 "\hspace{0.3cm} $\geq$50\% & " ///
        r14 "\hspace{0.1cm} SD of error &" ///
        r15 "\hspace{0.1cm} Correlation administrative and survey amounts &" ///
        r16 "\hspace{0.1cm} Observations &" ///
        r17 "\hline \textbf{\textit{All (true +/false +/false —)}} \\ \hspace{0.1cm}
            Mean administrative amount &" ///
        r18 "\hspace{0.1cm} Mean survey amount &" ///
        r19 "\hspace{0.1cm} Error in \% of administrative amount: \\ \hspace{0.3cm} $\
            leq$—50\% & " ///
        r20 "\hspace{0.3cm} $>$—50\% \& $\leq$—10\% & " ///
        r21 "\hspace{0.3cm} $>$—10\% \& $<$10\% & " ///
        r22 "\hspace{0.3cm} $\geq$10\% \& $<$50\% & " ///
        r23 "\hspace{0.3cm} $\geq$50\% (or undefined if false +) & " ///
        r24 "\hspace{0.1cm} SD of error &" ///
        r25 "\hspace{0.1cm} Correlation administrative and survey amounts &" ///
        r26 "\hspace{0.1cm} Observations &"

estout e(misreport, fmt("%15.1fc %15.0fc %15.1fc %15.0fc %15.1fc %15.0fc %15.0fc %15.0fc
```

```
    %15.1fc %15.1fc %15.1fc %15.1fc %15.1fc %15.0fc %15.2fc %15.0fc %15.0fc %15.0fc %15.1
    fc %15.1fc %15.1fc %15.1fc %15.1fc %15.0fc %15.2fc %15.0fc" ) )  ///
        using "${tabls}\tabl_misreport.tex", ///
        style(tex) abbrev wrap  mgroups(none) mlabels(none) ///
        label nolz varwidth(110) modelwidth(10) collabels(none) ///
        varlabels(${lab}) ///
        noomitted replace
```

## combinations_receipt.do

```
set more off
est clear
cap log close
log using "${log}\log_combinations_receipt.log", replace
*******************************************************************************************
    Table 3: Combinations of income receipt, given administrative receipt
*******************************************************************************************
set more off
use "${input}\data_analysis.dta", clear
keep if age>=16

* in receipt of unemployment benefits and earnings
foreach type in a s {
        global ui_`type' ui_rec`type'==1 & ua_rec`type'==0 & aclc_rec`type'==0 &
            earnspr_rec`type'==0 // UI only
        global ua_`type' ui_rec`type'==0 & ua_rec`type'==1 & aclc_rec`type'==0 &
            earnspr_rec`type'==0 // UA only
        global e_`type' ui_rec`type'==0 & ua_rec`type'==0 & aclc_rec`type'==0 &
            earnspr_rec`type'==1 // earnings only
        global ui_e_`type' ui_rec`type'==1 & ua_rec`type'==0 & aclc_rec`type'==0 &
            earnspr_rec`type'==1 // UI + earnings
        global ua_e_`type' ui_rec`type'==0 & ua_rec`type'==1 & aclc_rec`type'==0 &
            earnspr_rec`type'==1 // UA + earnings
        global ui_ua_`type' ui_rec`type'==1 & ua_rec`type'==1 & aclc_rec`type'==0 &
            earnspr_rec`type'==0 // UI + UA
        global ui_ua_e_`type' ui_rec`type'==1 & ua_rec`type'==1 & aclc_rec`type'==0 &
            earnspr_rec`type'==1 // UI + UA + earnings
        global none_`type' ui_rec`type'==0 & ua_rec`type'==0 & aclc_rec`type'==0 &
            earnspr_rec`type'==0 // no receipt
        global aclc_`type' aclc_rec`type'==1 // ACLC (alone or in combination of other
            incomes)
}

foreach inc1 in ui ua e aclc ui_e ua_e ui_ua ui_ua_e none {
        foreach inc2 in ui ua e aclc ui_e ua_e ui_ua ui_ua_e none {
                sum pid if ${`inc1'_a} & ${`inc2'_s}
                gen `inc1'a_`inc2's = r(N)      // number of observations with
                    combinations of survey and admin receipt
                replace `inc1'a_`inc2's = . if `inc1'a_`inc2's < 20      // do not
                    disclose cells with too few observations
        }
}
foreach type in a s {
        foreach inc in ui ua aclc e ui_e ua_e ui_ua ui_ua_e none {
                sum pid if ${`inc'_`type'}
                gen `inc'`type' = r(N)  // number of observations with survey or admin
                    receipt
                replace `inc'`type' = . if `inc'`type' < 20 // do not disclose cells with
                     too few observations

        }
}

foreach inc1 in ui ua aclc e ui_e ua_e ui_ua ui_ua_e none {
        gen p`inc1'a = 100
        foreach inc2 in ui ua aclc e ui_e ua_e ui_ua ui_ua_e none {
                gen p`inc1'a_`inc2's = `inc1'a_`inc2's/`inc1'a*100 // proportion of those
                     in admin category reporting income receipt in the survey
                gen pv`inc1'a_`inc2's = p`inc1'a_`inc2's/100 // variable used to
                    highlight cells depending on value
        }
}
```

```
* store values
foreach incl in ui ua aclc e ui_e ua_e ui_ua ui_ua_e none {
        estpost tabstat p`incl'a* pv`incl'a* `incl'a, stat(mean)
        est store ep`incl'a
}

estpost tabstat uis uas aclcs es ui_es ua_es ui_uas ui_ua_es nones, stat(mean)
est store ealls

* make table
local starsign "starlevels(\cellcolor{red!35} 1 \cellcolor{red!10} 0.4 \cellcolor{white}
    0.1)"
local diag "\cellcolor{gray!25}"
noi {
        foreach var in uia {
                estout ep`var' ///
                        using "${tabls}\tabl_combinations_receipt.tex" , cells("p`var'
                            _uis(par(`diag') fmt(%9.1fc)) p`var'_uas(star fmt(%9.1fc)
                            pvalue(pv`var'_uas)) p`var'_es(star fmt(%9.1fc) pvalue(pv`var
                            '_es)) p`var'_ui_es(star fmt(%9.1fc) pvalue(pv`var'_ui_es)) p
                            `var'_ua_es(star fmt(%9.1fc) pvalue(pv`var'_ua_es)) p`var'
                            _ui_uas(star fmt(%9.1fc) pvalue(pv`var'_ui_uas)) p`var'
                            _ui_ua_es(star fmt(%9.1fc) pvalue(pv`var'_ui_ua_es)) p`var'
                            _aclcs(star fmt(%9.1fc) pvalue(pv`var'_aclcs))  p`var'_nones(
                            star fmt(%9.1fc) pvalue(pv`var'_nones)) `var'(fmt(%9.0fc))")
                            ///
                        style(tex) abbrev wrap  mgroups(none) mlabels(none) `starsign'
                            ///
                        label nolz varwidth(38) modelwidth(5) collabels(none) ///
                        posthead("\textbf{\textit{admin data}} \\") ///
                        varlabels(mean "UI only") ///
                        noomitted replace
        }

        foreach var in uaa {
                estout ep`var' ///
                        using "${tabls}\tabl_combinations_receipt.tex" , cells("p`var'
                            _uis(star fmt(%9.1fc) pvalue(pv`var'_uis)) p`var'_uas(par(`
                            diag') fmt(%9.1fc)) p`var'_es(star fmt(%9.1fc) pvalue(pv`var'
                            _es)) p`var'_ui_es(star fmt(%9.1fc) pvalue(pv`var'_ui_es)) p`
                            var'_ua_es(star fmt(%9.1fc) pvalue(pv`var'_ua_es)) p`var'
                            _ui_uas(star fmt(%9.1fc) pvalue(pv`var'_ui_uas)) p`var'
                            _ui_ua_es(star fmt(%9.1fc) pvalue(pv`var'_ui_ua_es)) p`var'
                            _aclcs(star fmt(%9.1fc) pvalue(pv`var'_aclcs))  p`var'_nones(
                            star fmt(%9.1fc) pvalue(pv`var'_nones)) `var'(fmt(%9.0fc))")
                            ///
                        style(tex) abbrev wrap  mgroups(none) mlabels(none) `starsign'
                            ///
                        label nolz varwidth(38) modelwidth(5) collabels(none) ///
                        varlabels(mean "\hline UA only") ///
                        noomitted append
        }

        foreach var in ea {
                estout ep`var' ///
                        using "${tabls}\tabl_combinations_receipt.tex" , cells("p`var'
                            _uis(star fmt(%9.1fc) pvalue(pv`var'_uis)) p`var'_uas(star
                            fmt(%9.1fc) pvalue(pv`var'_uas)) p`var'_es(par(`diag') fmt
                            (%9.1fc)) p`var'_ui_es(star fmt(%9.1fc) pvalue(pv`var'_ui_es)
                            ) p`var'_ua_es(star fmt(%9.1fc) pvalue(pv`var'_ua_es)) p`var'
                            _ui_uas(star fmt(%9.1fc) pvalue(pv`var'_ui_uas)) p`var'
                            _ui_ua_es(star fmt(%9.1fc) pvalue(pv`var'_ui_ua_es)) p`var'
                            _aclcs(star fmt(%9.1fc) pvalue(pv`var'_aclcs))  p`var'_nones(
                            star fmt(%9.1fc) pvalue(pv`var'_nones)) `var'(fmt(%9.0fc))")
                            ///
                        style(tex) abbrev wrap  mgroups(none) mlabels(none) `starsign'
                            ///
                        label nolz varwidth(38) modelwidth(5) collabels(none) ///
                        varlabels(mean "\hline Earn. only") ///
                        noomitted append
        }

        foreach var in ui_ea {
```

```
        estout ep`var' ///
                using "${tabls}\tabl_combinations_receipt.tex" , cells("p`var'
                    _uis(star fmt(%9.1fc) pvalue(pv`var'_uis)) p`var'_uas(star
                    fmt(%9.1fc) pvalue(pv`var'_uas)) p`var'_es(star fmt(%9.1fc)
                    pvalue(pv`var'_es)) p`var'_ui_es(par(`diag') fmt(%9.1fc)) p`
                    var'_ua_es(star fmt(%9.1fc) pvalue(pv`var'_ua_es)) p`var'
                    _ui_uas(star fmt(%9.1fc) pvalue(pv`var'_ui_uas)) p`var'
                    _ui_ua_es(star fmt(%9.1fc) pvalue(pv`var'_ui_ua_es)) p`var'
                    _aclcs(star fmt(%9.1fc) pvalue(pv`var'_aclcs))  p`var'_nones(
                    star fmt(%9.1fc) pvalue(pv`var'_nones)) `var'(fmt(%9.0fc))")
                    ///
                style(tex) abbrev wrap  mgroups(none) mlabels(none) `starsign'
                    ///
                label nolz varwidth(38) modelwidth(5) collabels(none) ///
                varlabels(mean "\hline UI + earn.") ///
                noomitted append
}

foreach var in ua_ea {
        estout ep`var' ///
                using "${tabls}\tabl_combinations_receipt.tex" , cells("p`var'
                    _uis(star fmt(%9.1fc) pvalue(pv`var'_uis)) p`var'_uas(star
                    fmt(%9.1fc) pvalue(pv`var'_uas)) p`var'_es(star fmt(%9.1fc)
                    pvalue(pv`var'_es)) p`var'_ui_es(star fmt(%9.1fc) pvalue(pv`
                    var'_ui_es)) p`var'_ua_es(par(`diag') fmt(%9.1fc)) p`var'
                    _ui_uas(star fmt(%9.1fc) pvalue(pv`var'_ui_uas)) p`var'
                    _ui_ua_es(star fmt(%9.1fc) pvalue(pv`var'_ui_ua_es)) p`var'
                    _aclcs(star fmt(%9.1fc) pvalue(pv`var'_aclcs))  p`var'_nones(
                    star fmt(%9.1fc) pvalue(pv`var'_nones)) `var'(fmt(%9.0fc))")
                    ///
                style(tex) abbrev wrap  mgroups(none) mlabels(none) `starsign'
                    ///
                label nolz varwidth(38) modelwidth(5) collabels(none) ///
                varlabels(mean "\hline UA + earn.") ///
                noomitted append
}

foreach var in ui_uaa {
        estout ep`var' ///
                using "${tabls}\tabl_combinations_receipt.tex" , cells("p`var'
                    _uis(star fmt(%9.1fc) pvalue(pv`var'_uis)) p`var'_uas(star
                    fmt(%9.1fc) pvalue(pv`var'_uas)) p`var'_es(star fmt(%9.1fc)
                    pvalue(pv`var'_es)) p`var'_ui_es(star fmt(%9.1fc) pvalue(pv`
                    var'_ui_es)) p`var'_ua_es(star fmt(%9.1fc) pvalue(pv`var'
                    _ua_es)) p`var'_ui_uas(par(`diag') fmt(%9.1fc)) p`var'
                    _ui_ua_es(star fmt(%9.1fc) pvalue(pv`var'_ui_ua_es)) p`var'
                    _aclcs(star fmt(%9.1fc) pvalue(pv`var'_aclcs))  p`var'_nones(
                    star fmt(%9.1fc) pvalue(pv`var'_nones)) `var'(fmt(%9.0fc))")
                    ///
                style(tex) abbrev wrap  mgroups(none) mlabels(none) `starsign'
                    ///
                label nolz varwidth(38) modelwidth(5) collabels(none) ///
                varlabels(mean "\hline UI + UA") ///
                noomitted append
}

foreach var in ui_ua_ea {
        estout ep`var' ///
                using "${tabls}\tabl_combinations_receipt.tex" , cells("p`var'
                    _uis(star fmt(%9.1fc) pvalue(pv`var'_uis)) p`var'_uas(star
                    fmt(%9.1fc) pvalue(pv`var'_uas)) p`var'_es(star fmt(%9.1fc)
                    pvalue(pv`var'_es)) p`var'_ui_es(star fmt(%9.1fc) pvalue(pv`
                    var'_ui_es)) p`var'_ua_es(star fmt(%9.1fc) pvalue(pv`var'
                    _ua_es)) p`var'_ui_uas(star fmt(%9.1fc) pvalue(pv`var'_ui_uas
                    )) p`var'_ui_ua_es(par(`diag') fmt(%9.1fc)) p`var'_aclcs(star
                     fmt(%9.1fc) pvalue(pv`var'_aclcs))  p`var'_nones(star fmt
                    (%9.1fc) pvalue(pv`var'_nones)) `var'(fmt(%9.0fc))") ///
                style(tex) abbrev wrap  mgroups(none) mlabels(none) `starsign'
                    ///
                label nolz varwidth(38) modelwidth(5) collabels(none) ///
                varlabels(mean "\hline UI + UA + earn.") ///
                noomitted append
}
```

```
        foreach var in aclca {
                estout ep`var' ///
                        using "${tabls}\tabl_combinations_receipt.tex" , cells("p`var'
                            _uis(star fmt(%9.1fc) pvalue(pv`var'_uis)) p`var'_uas(star
                            fmt(%9.1fc) pvalue(pv`var'_uas)) p`var'_es(star fmt(%9.1fc)
                            pvalue(pv`var'_es)) p`var'_ui_es(star fmt(%9.1fc) pvalue(pv`
                            var'_ui_es)) p`var'_ua_es(star fmt(%9.1fc) pvalue(pv`var'
                            _ua_es)) p`var'_ui_uas(star fmt(%9.1fc) pvalue(pv`var'_ui_uas
                            )) p`var'_ui_ua_es(star fmt(%9.1fc) pvalue(pv`var'_ui_ua_es))
                             p`var'_aclcs(par(`diag') fmt(%9.1fc)) p`var'_nones(star fmt
                            (%9.1fc) pvalue(pv`var'_nones)) `var'(fmt(%9.0fc))") ///
                        style(tex) abbrev wrap  mgroups(none) mlabels(none) `starsign'
                            ///
                        label nolz varwidth(38) modelwidth(5) collabels(none) ///
                        varlabels(mean "\hline ACLC + others") ///
                        noomitted append
        }

        foreach var in nonea {
                estout ep`var' ///
                        using "${tabls}\tabl_combinations_receipt.tex" , cells("p`var'
                            _uis(star fmt(%9.1fc) pvalue(pv`var'_uis)) p`var'_uas(star
                            fmt(%9.1fc) pvalue(pv`var'_uas)) p`var'_es(star fmt(%9.1fc)
                            pvalue(pv`var'_es)) p`var'_ui_es(star fmt(%9.1fc) pvalue(pv`
                            var'_ui_es)) p`var'_ua_es(star fmt(%9.1fc) pvalue(pv`var'
                            _ua_es)) p`var'_ui_uas(star fmt(%9.1fc) pvalue(pv`var'_ui_uas
                            )) p`var'_ui_ua_es(star fmt(%9.1fc) pvalue(pv`var'_ui_ua_es))
                             p`var'_aclcs(star fmt(%9.1fc) pvalue(pv`var'_aclcs)) p`var'
                            _nones(par(`diag') fmt(%9.1fc)) `var'(fmt(%9.0fc))") ///
                        style(tex) abbrev wrap  mgroups(none) mlabels(none) `starsign'
                            ///
                        label nolz varwidth(38) modelwidth(5) collabels(none) ///
                        varlabels(mean "\hline none") ///
                        noomitted append
        }

        estout ealls ///
                using "${tabls}\tabl_combinations_receipt.tex" , cells("uis(fmt(%9.0fc))
                    uas(fmt(%9.0fc)) es(fmt(%9.0fc)) ui_es(fmt(%9.0fc)) ua_es(fmt(%9.0fc)
                    ) ui_uas(fmt(%9.0fc)) ui_ua_es(fmt(%9.0fc)) aclcs(fmt(%9.0fc)) nones(
                    fmt(%9.0fc))") ///
                style(tex) abbrev wrap  mgroups(none) mlabels(none) ///
                label nolz varwidth(38) modelwidth(5) collabels(none) ///
                varlabels(mean "\hline \textit{Observations}") ///
                noomitted append
}
log close
```

### reg_report_receipt.do

```
set more off
est clear
cap log close
log using "${log}\log_reg_report_receipt.log", replace
*******************************************************************************************************

* Table 4: Probability of reporting the unemployment insurance (UI) benefit in the survey
    , conditional on receiving it
* Table 5: Probability of reporting the unemployment assistance (UA) in the survey,
    conditional on receiving it
* Table A.1: Probability of reporting the unemployment insurance (UI) benefit in the
    survey, conditional on receiving it
* Table A.3: Probability of reporting the unemployment assistance (UA) in the survey,
    conditional on receiving it
* Table A.5: Probability of reporting the unemployment insurance (UI) benefit in the
    survey, conditional on receiving it, by gender
* Table A.6: Probability of reporting the unemployment assistance (UA) in the survey,
    conditional on receiving it, by gender
*******************************************************************************************************

use "${input}\data_analysis.dta", clear
keep if age>=16
```

```
* unemployment insurance (UI)
foreach var in ui {
        foreach var2 in ua {
                foreach var3 in aclc {
                        reg `var'_reportrec ln`var'_a tearnspr_g_a tdearnspr ib3.
                            earnspr_false ib3.`var2'_false ib3.`var3'_false if `var'
                            _reportrec>-1, vce(cluster pid)
                        estimates store `var'nc_reportrec

                        local vars_reg sex ib8.age_gr ib1.nad ib0.nch ib1.edu ib1.region
                            ib10.occup ib3.industry ib0.proxy_partner incouple ib1.folge
                            ib1.folge#ib2008.year ib0.civil_serv ib3.int_month `var'
                            _months_a ib1.int_type ib1.int_same ib1.country_birth ib5.
                            health ib0.paid_jobcourse
                        reg `var'_reportrec ln`var'_a tearnspr_g_a tdearnspr ib3.
                            earnspr_false ib3.`var2'_false ib3.`var3'_false `vars_reg' if
                             `var'_reportrec>-1, vce(cluster pid)
                        estimates store `var'_reportrec
                }
        }
}

* unemployment assistance (UA)
foreach var in ua {
        foreach var2 in ui {
                foreach var3 in aclc {
                        reg `var'_reportrec ln`var'_a tearnspr_g_a tdearnspr ib3.
                            earnspr_false ib3.`var2'_false ib3.`var3'_false if `var'
                            _reportrec>-1, vce(cluster pid)
                        estimates store `var'nc_reportrec

                        local vars_reg sex ib8.age_gr ib1.nad ib0.nch ib1.edu ib1.region
                            ib10.occup ib3.industry ib0.proxy_partner incouple ib1.folge
                            ib1.folge#ib2008.year ib0.civil_serv ib3.int_month `var'
                            _months_a ib1.int_type ib1.int_same ib1.country_birth ib5.
                            health ib0.paid_jobcourse
                        reg `var'_reportrec ln`var'_a tearnspr_g_a tdearnspr ib3.
                            earnspr_false ib3.`var2'_false ib3.`var3'_false `vars_reg' if
                             `var'_reportrec>-1, vce(cluster pid)
                        estimates store `var'_reportrec
                }
        }
}

***** by gender
foreach s in 0 1 {
        * UI
        foreach var in ui {
                foreach var2 in ua {
                        foreach var3 in aclc {
                                reg `var'_reportrec ln`var'_a tearnspr_g_a tdearnspr ib3.
                                    earnspr_false ib3.`var2'_false ib3.`var3'_false if `
                                    var'_reportrec>-1 & sex==`s', vce(cluster pid)
                                estimates store `var'nc_reportrec`s'

                                local vars_reg ib8.age_gr ib1.nad ib0.nch ib1.edu ib1.
                                    region ib10.occup ib3.industry ib0.proxy_partner
                                    incouple ib1.folge ib1.folge#ib2008.year ib0.
                                    civil_serv ib3.int_month `var'_months_a ib1.int_type
                                    ib1.int_same ib1.country_birth ib5.health ib0.
                                    paid_jobcourse
                                reg `var'_reportrec ln`var'_a tearnspr_g_a tdearnspr ib3.
                                    earnspr_false ib3.`var2'_false ib3.`var3'_false `
                                    vars_reg' if `var'_reportrec>-1 & sex==`s', vce(
                                    cluster pid)
                                estimates store `var'_reportrec`s'
                        }
                }
        }

        * UA
        foreach var in ua {
```

```
                foreach var2 in ui {
                        foreach var3 in aclc {
                                reg ‘var’_reportrec ln‘var’_a tearnspr_g_a tdearnspr ib3.
                                    earnspr_false ib3.‘var2’_false ib3.‘var3’_false if ‘
                                    var’_reportrec>-1 & sex==‘s’, vce(cluster pid)
                                estimates store ‘var’nc_reportrec‘s’

                                local vars_reg ib8.age_gr ib1.nad ib0.nch ib1.edu ib1.
                                    region ib10.occup ib3.industry ib0.proxy_partner
                                    incouple ib1.folge ib1.folge#ib2008.year ib0.
                                    civil_serv ib3.int_month ‘var’_months_a ib1.int_type
                                    ib1.int_same ib1.country_birth ib5.health ib0.
                                    paid_jobcourse
                                reg ‘var’_reportrec ln‘var’_a tearnspr_g_a tdearnspr ib3.
                                    earnspr_false ib3.‘var2’_false ib3.‘var3’_false ‘
                                    vars_reg’ if ‘var’_reportrec>-1 & sex==‘s’, vce(
                                    cluster pid)
                                estimates store ‘var’_reportrec‘s’
                        }
                }
        }
}
*************************************************************************
* output reg tables
*************************************************************************
qui do "${do}\tables_labels.do"

* tables for paper
foreach var in ui ua {
        estout ‘var’nc_reportrec ‘var’_reportrec ///
                using "${tabls}\reg_‘var’_receipt.tex" , cells(b(star fmt(%9.3f)) (se(par
                     fmt(%9.3f)) p(fmt(%9.3f)))) stats(r2_a N, fmt(%9.3f %9.0g) labels(R-
                    squared Observations)) stardrop(*) style(tex) abbrev wrap mlabels(
                    none) label nolz varwidth(60) modelwidth(16) collabels(none)
                    noomitted drop(${labels_shdrop‘var’}) varlabels(${labels_‘var’},)
                    refcat(${labels_ref‘var’}, label(ref)) order(${labels_order‘var’})
                    indicate(\hline Controls=${labels_controls‘var’}) replace
}

* tables for online supplementary materials
foreach var in ui ua {
        estout ‘var’nc_reportrec ‘var’_reportrec ///
                using "${tabls}\reg_complete_‘var’_receipt.tex" , cells(b(star fmt(%9.3f)
                    ) (se(par fmt(%9.3f)) p(fmt(%9.3f)))) stats(r2_a N, fmt(%9.3f %9.0g)
                    labels(R-squared Observations)) stardrop(*) style(tex) abbrev wrap
                    mlabels(none) label nolz varwidth(60) modelwidth(16) collabels(none)
                    noomitted drop(${labels_drop‘var’}) varlabels(${labels_‘var’},)
                    refcat(${labels_ref‘var’}, label(ref)) order(${labels_order‘var’})
                    replace

        estout ‘var’nc_reportrec0 ‘var’_reportrec0 ‘var’nc_reportrec1 ‘var’_reportrec1
            ///
                using "${tabls}\reg_‘var’_receiptbygen.tex" , cells(b(star fmt(%9.3f)) (
                    se(par fmt(%9.3f)) p(fmt(%9.3f)))) stats(r2_a N, fmt(%9.3f %9.0g)
                    labels(R-squared Observations)) stardrop(*) style(tex) abbrev wrap
                    mlabels(none) label nolz varwidth(60) modelwidth(16) collabels(none)
                    noomitted drop(${labels_shdrop‘var’s}) varlabels(${labels_‘var’},)
                    refcat(${labels_ref‘var’s}, label(ref)) order(${labels_order‘var’s})
                    indicate(\hline Controls=${labels_controls‘var’}) replace
}

log close
```

## mean_amounts.do

```
set more off
est clear
cap scalar drop _all
cap matrix drop _all
cap log close
log using "${log}\log_mean_amounts.log", replace
**************************************************************************************
* Table 6: Mean survey and administrative amounts by benefits true positive , false
```

```
    negative
*******************************************************************************************
use "${input}\data_analysis.dta", clear

keep if age>=16

*************************************************************
* do people lump benefits and earnings together when they report them
*************************************************************
cap drop *ub*_*
gen ube_g_s = ui_g_s + ua_g_s + aclc_g_s + earnspr_g_s
gen ube_g_a = ui_g_a + ua_g_a + aclc_g_a + earnspr_g_a
foreach var in ui ua aclc earnspr ube {
        gen error_`var'_g = `var'_g_s - `var'_g_a // error = survey - admin amount
}

* compare the mean for each benefit and earnings
set more off
mat meanamt = J(48,6,.z) // in J() first n should be as many rows as variables in local
    varlist
local row = 1
local ui_false4 1
local ui_false2 7
local ui_false1 13
local ua_false4 19
local ua_false2 25
local ua_false1 31
local aclc_false2 37
local aclc_false1 43
set trace off
* UI UA
foreach var in ui ua {
        foreach f in 4 2 1 { // 4=true +, 2 = false -, 1 = false +
                foreach var1 in ui_g {
                        foreach var2 in ua_g {
                                foreach var3 in aclc_g {
                                        * conditional on admin receipt
                                        global cond1 (`var'_false==`f')  // conditional
                                            on admin receipt
                                        global cond2 `var1'_a!=. & `var2'_a!=. & `var3'_a
                                            !=. & `var1'_s!=. & `var2'_s!=. & `var3'_s!=.
                                             & earnspr_g_s!=. & earnspr_g_a!=.
                                        tabstat `var1'_s `var2'_s `var3'_s earnspr_g_s
                                            ube_g_s if $cond1 & $cond2, stat(mean) save
                                        matrix tots=r(StatTotal)
                                        local row=``var'_false`f''
                                        forvalues r=1(1)5 { // column 1=survey amounts
                                                mat meanamt[`row', 1] = tots[1,`r']
                                                local row=`row'+1
                                        }
                                        tabstat `var1'_a `var2'_a `var3'_a earnspr_g_a
                                            ube_g_a if $cond1 & $cond2, stat(mean) save
                                        matrix tota=r(StatTotal)
                                        local row=``var'_false`f''
                                        forvalues r=1(1)5 { // column 2=admin amounts
                                                mat meanamt[`row', 2] = tota[1,`r']
                                                local row=`row'+1
                                        }
                                        local row=``var'_false`f''
                                        forvalues r=1(1)5 { // column 3= error (admin-
                                            survey amounts)
                                                mat meanamt[`row', 3] = tots[1,`r'] -
                                                    tota[1,`r']
                                                local row=`row'+1
                                        }
                                        local row=``var'_false`f''
                                        forvalues r=1(1)5 { // column 4=error in % of
                                            mean admin income (all benefits + earnings)
                                                mat meanamt[`row', 4] = (tots[1,`r'] -
                                                    tota[1,`r']) / tota[1,5] * 100
                                                local row=`row'+1
                                        }
```

```
                                        tabstat error_`var1' error_`var2' error_`var3'
                                            error_earnspr_g error_ube_g if $cond1 &
                                            $cond2, stat(sd) save
                                        matrix error=r(StatTotal)
                                        local row=``var'_false`f''
                                        forvalues r=1(1)5 { // column 5=sd of error
                                                mat meanamt[`row', 5] = error[1,`r']
                                                local row=`row'+1
                                        }

                                        cap drop corr*
                                        foreach v in `var1' `var2' `var3' earnspr_g ube_g
                                             {
                                                corr `v'_a `v'_s if $cond1 & $cond2
                                                gen corr_`v' = r(rho)
                                        }
                                        tabstat corr_`var1' corr_`var2' corr_`var3'
                                            corr_earnspr_g corr_ube_g if $cond1 & $cond2,
                                             stat(mean) save
                                        matrix corr=r(StatTotal)
                                        local row=``var'_false`f''
                                        forvalues r=1(1)5 { // column 6=correlation bw
                                            admin and survey amounts
                                                mat meanamt[`row', 6] = corr[1,`r']
                                                local row=`row'+1
                                        }
                                        tabstat `var1'_a `var2'_a `var3'_a earnspr_g_a
                                            ube_g_a if $cond1 & $cond2, stat(n) save
                                        matrix n=r(StatTotal)
                                        forvalues r=1(1)1 { // n observations
                                                mat meanamt[`row', `r'] = n[1,`r']
                                        }
                                }
                        }
                }
        }
}


* aclc
* too few cases of ACLC true +, so cannot disclose in table
foreach var in aclc {
        foreach f in 2 1 { // 2 = false -, 1 = false +
                foreach var1 in ui_g {
                        foreach var2 in ua_g {
                                foreach var3 in aclc_g {
                                        * conditional on admin receipt
                                        global cond1 (`var'_false==`f')  // conditional
                                            on admin receipt
                                        global cond2 `var1'_a!=. & `var2'_a!=. & `var3'_a
                                            !=. & `var1'_s!=. & `var2'_s!=. & `var3'_s!=.
                                             & earnspr_g_s!=. & earnspr_g_a!=.
                                        tabstat `var1'_s `var2'_s `var3'_s earnspr_g_s
                                            ube_g_s if $cond1 & $cond2, stat(mean) save
                                        matrix tots=r(StatTotal)
                                        local row=``var'_false`f''
                                        forvalues r=1(1)5 { // column 1=survey amounts
                                                mat meanamt[`row', 1] = tots[1,`r']
                                                local row=`row'+1
                                        }
                                        tabstat `var1'_a `var2'_a `var3'_a earnspr_g_a
                                            ube_g_a if $cond1 & $cond2, stat(mean) save
                                        matrix tota=r(StatTotal)
                                        local row=``var'_false`f''
                                        forvalues r=1(1)5 { // column 2=admin amounts
                                                mat meanamt[`row', 2] = tota[1,`r']
                                                local row=`row'+1
                                        }
                                        local row=``var'_false`f''
                                        forvalues r=1(1)5 { // column 3= admin-survey
                                            amounts
                                                mat meanamt[`row', 3] = tots[1,`r'] -
                                                    tota[1,`r']
```

```
                                        local row=`row'+1
                                }
                                local row=``var'_false`f''
                                forvalues r=1(1)5 { // column 5=error in % of
                                    mean admin income (all benefits + earnings)
                                        mat meanamt[`row', 4] = (tots[1,`r'] -
                                            tota[1,`r']) / tota[1,5] * 100
                                        local row=`row'+1
                                }

                                tabstat error_`var1' error_`var2' error_`var3'
                                    error_earnspr_g error_ube_g if $cond1 &
                                    $cond2, stat(sd) save
                                matrix error=r(StatTotal)
                                local row=``var'_false`f''
                                forvalues r=1(1)5 { // column 4=error
                                        mat meanamt[`row', 5] = error[1,`r']
                                        local row=`row'+1
                                }

                                cap drop corr*
                                foreach v in `var1' `var2' `var3' earnspr_g ube_g
                                     {
                                        corr `v'_a `v'_s if $cond1 & $cond2
                                        gen corr_`v' = r(rho)
                                }
                                tabstat corr_`var1' corr_`var2' corr_`var3'
                                    corr_earnspr_g corr_ube_g if $cond1 & $cond2,
                                     stat(mean) save
                                matrix corr=r(StatTotal)
                                local row=``var'_false`f''
                                forvalues r=1(1)5 { // column 5=correlation bw
                                    admin and survey amounts
                                        mat meanamt[`row', 6] = corr[1,`r']
                                        local row=`row'+1
                                }
                                tabstat `var1'_a `var2'_a `var3'_a earnspr_g_a
                                    ube_g_a if $cond1 & $cond2, stat(n) save
                                matrix n=r(StatTotal)
                                forvalues r=1(1)1 { // n observations
                                        mat meanamt[`row', `r'] = n[1,`r']
                                }
                        }
                }
        }
    }
}

estadd matrix meanamt = meanamt, replace
matrix list meanamt, nodotz

global lab r1 "\textbf{Conditional on UI true +} \\ \hspace{0.2cm} UI" ///
        r2 "\hspace{0.2cm} UA" ///
        r3 "\hspace{0.2cm} ACLC" ///
        r4 "\hspace{0.2cm} Earnings" ///
        r5 "\hspace{0.2cm} Income" ///
        r6 "\hspace{0.2cm} Observations" ///
        r7 "\textbf{Conditional on UI false --} \\ \hspace{0.2cm} UI" ///
        r8 "\hspace{0.2cm} UA" ///
        r9 "\hspace{0.2cm} ACLC" ///
        r10 "\hspace{0.2cm} Earnings" ///
        r11 "\hspace{0.2cm} Income" ///
        r12 "\hspace{0.2cm} Observations" ///
        r13 "\textbf{Conditional on UI false +} \\ \hspace{0.2cm} UI" ///
        r14 "\hspace{0.2cm} UA" ///
        r15 "\hspace{0.2cm} ACLC" ///
        r16 "\hspace{0.2cm} Earnings" ///
        r17 "\hspace{0.2cm} Income" ///
        r18 "\hspace{0.2cm} Observations" ///
        r19 "\hline \textbf{Conditional on UA true +} \\ \hspace{0.2cm} UI" ///
        r20 "\hspace{0.2cm} UA" ///
        r21 "\hspace{0.2cm} ACLC" ///
        r22 "\hspace{0.2cm} Earnings" ///
```

```
        r23 "\hspace{0.2cm} Income" ///
        r24 "\hspace{0.2cm} Observations" ///
        r25 "\textbf{Conditional on UA false --} \\ \hspace{0.2cm} UI" ///
        r26 "\hspace{0.2cm} UA" ///
        r27 "\hspace{0.2cm} ACLC" ///
        r28 "\hspace{0.2cm} Earnings" ///
        r29 "\hspace{0.2cm} Income" ///
        r30 "\hspace{0.2cm} Observations" ///
        r31 "\textbf{Conditional on UA false +} \\ \hspace{0.2cm} UI" ///
        r32 "\hspace{0.2cm} UA" ///
        r33 "\hspace{0.2cm} ACLC" ///
        r34 "\hspace{0.2cm} Earnings" ///
        r35 "\hspace{0.2cm} Income" ///
        r36 "\hspace{0.2cm} Observations" ///
        r37 "\hline \textbf{Conditional on ACLC false --} \\ \hspace{0.2cm} UI" ///
        r38 "\hspace{0.2cm} UA" ///
        r39 "\hspace{0.2cm} ACLC" ///
        r40 "\hspace{0.2cm} Earnings" ///
        r41 "\hspace{0.2cm} Income" ///
        r42 "\hspace{0.2cm} Observations" ///
        r43 "\textbf{Conditional on ACLC false +} \\ \hspace{0.2cm} UI" ///
        r44 "\hspace{0.2cm} UA" ///
        r45 "\hspace{0.2cm} ACLC" ///
        r46 "\hspace{0.2cm} Earnings" ///
        r47 "\hspace{0.2cm} Income" ///
        r48 "\hspace{0.2cm} Observations"

estout e(meanamt, fmt(%15.0fc %15.0fc %15.0fc %15.2fc %15.0fc %15.2fc)) ///
        using "${tabls}\tabl_meanamt.tex", ///
        style(tex) abbrev wrap  mgroups(none) mlabels(none) ///
        label nolz varwidth(75) modelwidth(15) collabels(none) ///
        varlabels(${lab}) ///
        noomitted replace

cap log close
```

## error_earnings.do

```
set more off
est clear
cap log close
log using "${log}\log_error_earnings.log", replace
*****************************************************************************************************
* Table 7: The mean of survey-admin earnings, conditional on overreporting earnings and
    receiving benefits in period t while not receiving benefits in period s
*****************************************************************************************************

set more off
use "${input}\data_analysis.dta", clear
keep if age>=16

* average annual earnings in 2007 = 27,458; 2008 = 28,255; 2009 = 28,537; 2010 = 28,715;
    note that calendar year=survey year - 1; Source: Statistik Austria, http://www.
    statistik.at/web_de/statistiken/preise/verbraucherpreisindex_vpi_hvpi/index.html
foreach var in dearnspr { // adjust 2008-2010 earnings to 2011 earnings
        replace `var' = `var'*104.5779/100 if year==2008
        replace `var' = `var'*101.628/100 if year==2009
        replace `var' = `var'*100.6238/100 if year==2010
}

*** overreports earnings in the survey relative to admin and...
**...with true positive for the sum of unemployment benefits
* correctly reports with amounts deviating by less than 10%
gen sample_truep_corr10 = [(earnspr_g_s > earnspr_g_a & earnspr_g_s!=. & earnspr_g_a!=.)
    & uben_false==4 & abs(1 - uben_g_s/uben_g_a)<0.1]

* misreports with amounts deviating by 10% or more
gen sample_truep_misr10 = [(earnspr_g_s > earnspr_g_a & earnspr_g_s!=. & earnspr_g_a!=.)
    & uben_false==4 & abs(1 - uben_g_s/uben_g_a)>=0.1]

**...with false negative for the sum of unemployment benefits
gen sample_benfalsen = [(earnspr_g_s > earnspr_g_a & earnspr_g_s!=. & earnspr_g_a!=.) &
```

```
    uben_false==2]

xtset pid folge // tell stata these are panel data

* in sample in period t but with true negative for the sum of unemployment benefits in t
    -/+ 1, 2, 3
foreach s in l f { // lag or lead
        foreach type in benfalsen truep_corr10 truep_misr10 {
                forvalues i=1(1)3 { // period t -/+ 1, 2, 3
                        gen `s'`i'_dearnspr_`type' = `s'`i'.dearnspr if sample_`type' ==
                            1 & `s'`i'.earnspr_reca!=-1 & `s'`i'.earnspr_recs!=-1 & `s'`i
                            '.uben_false==3
                }
        }
}

* mean earnings over-reports within individuals
foreach type in benfalsen truep_corr10 truep_misr10 {
        * in period t
        gen temp = sample_`type' if sample_`type' == 1
        bys pid: egen m_dearnspr_`type'_t = mean(dearnspr*temp)
        drop temp

        * in period s
        foreach var in l1_dearnspr l2_dearnspr l3_dearnspr f1_dearnspr f2_dearnspr
            f3_dearnspr {
                bys pid: egen m_`var'_`type' = mean(`var'_`type')
        }

        egen m_dearnspr_`type'_s = rowmean(m_l1_dearnspr_`type' m_l2_dearnspr_`type'
            m_l3_dearnspr_`type' m_f1_dearnspr_`type' m_f2_dearnspr_`type' m_f3_dearnspr_
            `type') // the mean across all lag and lead periods
}
sort pid folge
keep if pid[_n]!=pid[_n+1] // for each individual, keep last wave's observation

*** make table
mat overreport = J(3,3,.z)
local row = 1
local col = 1
foreach type in benfalsen truep_corr10 truep_misr10 {
        tabstat m_dearnspr_`type'_t m_dearnspr_`type'_s if m_dearnspr_`type'_t!=. &
            m_dearnspr_`type'_s!=., stat(mean n) save
        matrix tots=r(StatTotal) // if few cases, do not disclose
        if tots[2,1] >=20 mat overreport[`row', 1] = tots[2,1]
        if tots[2,1] >=20 mat overreport[`row', 2] = tots[1,1]
        if tots[2,1] >=20 mat overreport[`row', 3] = tots[1,2]

        local row = `row' + 1
}
estadd matrix overreport = overreport, replace
matrix list overreport, nodotz

global lab r1 "\textit{Benefits in t} \\ \hspace{0.1cm} false --" ///
        r2 "\hspace{0.1cm} true + \& abs error in sum of benefits \\ \hspace{0.3cm} $<$
            10\%" ///
        r3 "\hspace{0.3cm} $\geq$ 10\%"

estout e(overreport, fmt(%15.0fc %15.0fc)) ///
        using "${tabls}\tabl_earns_overreport.tex", ///
        style(tex) abbrev wrap mgroups(none) mlabels(none) ///
        label nolz varwidth(110) modelwidth(10) collabels(none) ///
        varlabels(${lab}) ///
        noomitted replace

cap log close
```

## reg_error_earnings_on_ben.do

```
set more off
est clear
cap log close
log using "${log}\log_reg_error_earnings_on_ben.log", replace
```

```
*****************************************************************************************************
* Table 8: OLS model of the error in earnings (surveyadmin earnings) on the reverse
    error in sum of benefits (adminsurvey benefits)
*****************************************************************************************************

use "${input}\data_analysis.dta", clear

keep if age>=16

foreach var in uben {
        gen error_`var' = `var'_g_a - `var'_g_s // benefits error = admin - survey amount
}
foreach var in earnspr {
        gen error_`var' = `var'_g_s - `var'_g_a // earnings error = survey - admin amount
}

replace uben_false = 6 if uben_false == 5 // missing/ imputed
replace uben_false = 5 if uben_false == 4 & error_uben <= 0 // true + & survey overreport
     (error<=0)
*uben_false = 4 remains true + & survey underreport (error>0)

label define false2 1 "false +" 2 "false -" 3 "true -" 4 "true + & survey underreport" 5
    "true + & survey overreports" 6 "missing/imputed"
label values uben_false false2

* reg error in earnings on reverse error in sum of benefits
foreach s in 0 1 {
        reg error_earnspr error_uben if sex==`s', vce(cluster pid)
        estimates store error_nc`s'

        local vars_reg ib8.age_gr ib1.nad ib0.nch ib1.edu ib1.region ib10.occup ib3.
            industry ib0.proxy_partner incouple ib1.folge ib1.folge#ib2008.year ib0.
            civil_serv ib3.int_month ib1.earnspr_months_a ib1.int_type ib1.int_same ib1.
            country_birth ib5.health
        reg error_earnspr error_uben `vars_reg' if sex==`s', vce(cluster pid)
        estimates store error_`s'
}

* reg error in earnings on revese error in sum of benefits interacted with misreporting
    type
foreach s in 0 1 {
        reg error_earnspr ib3.uben_false#c.error_uben if sex==`s', vce(cluster pid)
        estimates store error_uben_nc`s'

        local vars_reg ib8.age_gr ib1.nad ib0.nch ib1.edu ib1.region ib10.occup ib3.
            industry ib0.proxy_partner incouple ib1.folge ib1.folge#ib2008.year ib0.
            civil_serv ib3.int_month ib1.earnspr_months_a ib1.int_type ib1.int_same ib1.
            country_birth ib5.health
        reg error_earnspr ib3.uben_false#c.error_uben `vars_reg' if sex==`s', vce(cluster
             pid)
        estimates store error_uben_`s'
}
*************************************************************************
* output reg tables
*************************************************************************
qui do "${do}\tables_labels.do"

foreach var in error_earnspr {
        estout error_0 error_uben_0 error_1 error_uben_1 ///
                using "${tabls}\reg_errorearns_errorben.tex", cells(b(star fmt(%9.3f)) (
                    se(par fmt(%9.3f)) p(fmt(%9.3f)))) stats(r2_a N, fmt(%9.3f %9.0g)
                    labels(R-squared Observations)) stardrop(*) style(tex) abbrev
                    mlabels(none) label nolz varwidth(60) modelwidth(16) collabels(none)
                    noomitted /*drop(${labels_shdrop`var'})*/ varlabels(${labels_`var'},)
                     refcat(${labels_ref`var'}, label(ref)) order(${labels_order`var'})
                    indicate(\hline Controls=${labels_controls`var'}) replace
}

cap log close
```

## recipient_status_by_education.do

```
set more off
est clear
cap log close
log using "${log}\log_recipient_status_by_education.log", replace
*******************************************************
* Table 9: Education shares (%) by unemployment benefits status
*******************************************************
set more off
use "${input}\data_analysis.dta", clear
keep if age>18 & age<65

gen one = 1
mat eduub = J(4,8,0)
local row = 1
local col = 1
// separate columns (4x4) for non-recipients and recipients
* non-recipients
foreach s in 0 1 { // men vs women
	*** survey data
	local row = 1
	tabstat one if ((ui_reca!=-1 & ui_recs == 0) & (ua_reca!=-1 & ua_recs == 0) & (
	    aclc_reca!=-1 & aclc_recs == 0)) & sex == `s', by(edu) stat(n) save
	matrix tots=r(StatTotal)
	mat edu1 = r(Stat1)
	mat edu2 = r(Stat2)
	mat edu3 = r(Stat3)
	local row=`row'
	forvalues r=1(1)3 {
		mat eduub[`row', `col'] = edu`r'[1,1] / tots[1,1] * 100
		local row=`row'+1
	}
	mat eduub[`row', `col'] = tots[1,1]
	local col = `col' + 1
	*** admin data
	local row = 1
	tabstat one if ((ui_recs!=-1 & ui_reca == 0) & (ua_recs!=-1 & ua_reca == 0) & (
	    aclc_recs!=-1 & aclc_reca == 0)) & sex == `s', by(edu) stat(n) save
	matrix tots=r(StatTotal)
	mat edu1 = r(Stat1)
	mat edu2 = r(Stat2)
	mat edu3 = r(Stat3)
	local row=`row'
	forvalues r=1(1)3 {
		mat eduub[`row', `col'] = edu`r'[1,1] / tots[1,1] * 100
		local row=`row'+1
	}
	mat eduub[`row', `col'] = tots[1,1]
	local col = `col' + 1
} // end of gender loop

* recipients
foreach s in 0 1 { // men vs women
	*** survey data
	local row = 1
	tabstat one if ((ui_reca!=-1 & ui_recs == 1) | (ua_reca!=-1 & ua_recs == 1) | (
	    aclc_reca!=-1 & aclc_recs == 1)) & sex == `s', by(edu) stat(n) save
	matrix tots=r(StatTotal)
	mat edu1 = r(Stat1)
	mat edu2 = r(Stat2)
	mat edu3 = r(Stat3)
	local row=`row'
	forvalues r=1(1)3 {
		mat eduub[`row', `col'] = edu`r'[1,1] / tots[1,1] * 100
		local row=`row'+1
	}
	mat eduub[`row', `col'] = tots[1,1]
	local col = `col' + 1

	*** admin data
	local row = 1
	tabstat one if ((ui_recs!=-1 & ui_reca == 1) | (ua_recs!=-1 & ua_reca == 1) | (
	    aclc_recs!=-1 & aclc_reca == 1)) & sex == `s', by(edu) stat(n) save
	matrix tots=r(StatTotal)
```

```
        mat edu1 = r(Stat1)
        mat edu2 = r(Stat2)
        mat edu3 = r(Stat3)
        local row=`row'
        forvalues r=1(1)3 {
                mat eduub[`row', `col'] = edu`r'[1,1] / tots[1,1] * 100
                local row=`row'+1
        }
        mat eduub[`row', `col'] = tots[1,1]
        local col = `col' + 1
} // end of gender loop
estadd matrix eduub = eduub, replace
matrix list eduub

global lab r1 "low" ///
        r2 "middle" ///
        r3 "high" ///
        r4 "\hline Observations" ///

estout e(eduub, fmt("%15.1fc %15.1fc %15.1fc %15.0fc" ) )  ///
        using "${tabls}\eduub.tex", ///
        style(tex) abbrev wrap  mgroups(none) mlabels(none) ///
        label nolz varwidth(110) modelwidth(10) collabels(none) ///
        varlabels(${lab}) ///
        noomitted replace

log close
```

## application_bias_returns_education.do

```
set more off
est clear
cap log close
log using "${log}\log_application_bias_returns_education.log", replace
************************************************************************************************
* Table 10: Returns to education: log-earnings regression
* Table 11: Returns to education: log-earnings regression (sample with both positive
    administrative and survey earnings)
************************************************************************************************

set more off
use "${input}\data_analysis.dta", clear
keep if age>18 & age<65

** create a dummy for full-year full-time employed
* - use info on earnings, months receiving earnings and self-reported main economic
    status
foreach type in s a {
        gen fyear_ftime_emp_`type' = ([earnspr_months_`type'==12 & act_ftime_emp==1 &
            earnspr_g_`type'!=0 & earnspr_g_`type'!=. ])
}
label var fyear_ftime_emp_a "Admin: full-year full-time employed"
label var fyear_ftime_emp_s "Survey: full-year full-time employed"

* - replace the 0s with 1s for those who have been unemployed, but could have worked full
    -year
foreach type in s a {
        * full-time employed and unemployed throughout the year
        replace fyear_ftime_emp_`type' = 1 if (act_ftime_emp_months + act_unemp_months)
            == 12

        * receiving unemployment benefits for the whole year
        global cond_unemp fyear_ftime_emp_`type'==0
        replace fyear_ftime_emp_`type' = 1 if ui_months_`type'>=12 & ui_months_`type'!=.
            & ${cond_unemp}
        replace fyear_ftime_emp_`type' = 1 if ua_months_`type'>=12 & ua_months_`type'!=.
            & ${cond_unemp}
        replace fyear_ftime_emp_`type' = 1 if aclc_months_`type'>=12 & aclc_months_`type
            '!=. & ${cond_unemp}
        replace fyear_ftime_emp_`type' = 1 if (ui_months_`type' + ua_months_`type')>=12 &
             ///
                ((ui_months_`type'>0 & ui_months_`type'!=.) & (ua_months_`type'>0 &
```

```
                        ua_months_`type'!=.)) & ${cond_unemp}

        * with unemployment benefits spells
        gen unemp_`type' = .
        replace unemp_`type' = 1 if (ui_rec`type'==1 | ua_rec`type'==1 | aclc_rec`type
            '==1)
}

* define regression sample
foreach type in s a {
        gen sample_emp_`type' = [fyear_ftime_emp_`type'==1 & ui_rec`type' != -1 & ua_rec`
            type' != -1 & aclc_rec`type' != -1] // sample restriction
}

* intermediate earnings measure
gen lnearnspr_interm_s = lnearnspr_s // survey measure
gen lnearnspr_interm_a = lnearnspr_s // survey measure
replace lnearnspr_interm_a = lnearnspr_a if unemp_a == 1 // replace with admin measure if
     in receipt of admin benefits for any spell

* reg earnings on education
foreach var1 in lnearnspr { // outcome variable is log of earnings
        foreach s in 0 1 { // by sex
                foreach type in s a { // survey vs admin

                        local vars_reg ib8.age_gr ib1.region ib0.civil_serv ib1.
                            country_birth ib0.proxy_partner ib1.folge ib1.folge#ib2008.
                            year ib3.int_month ib1.int_type ib1.int_same

                        * different survey and admin samples
                        reg `var1'_`type' ib1.edu `vars_reg' if sample_emp_`type'==1 &
                            sex==`s', vce(cluster pid)
                        est store `var1'_unr_edu_`type'`s'

                        * restrict to the same survey and admin sample
                        reg `var1'_`type' ib1.edu `vars_reg' if `var1'_a!=. & `var1'_s!=.
                             & sample_emp_a==1 & sample_emp_s==1 & sex==`s', vce(cluster
                            pid)
                        est store `var1'_r_edu_`type'`s'

                        reg `var1'_interm_`type' ib1.edu `vars_reg' if `var1'_a!=. & `
                            var1'_s!=. & sample_emp_a==1 & sample_emp_s==1 & sex==`s',
                            vce(cluster pid)
                        est store `var1'_rinterm_edu_`type'`s'

                }
        }
}

*** Wald test if returns to education based on two datasets are equivalent
foreach var1 in lnearnspr { // outcome variable is log of earnings
        foreach s in 0 1 { // by sex
                foreach type in s a { // survey vs admin

                        local vars_reg ib8.age_gr ib1.region ib0.civil_serv ib1.
                            country_birth ib0.proxy_partner ib1.folge ib1.folge#ib2008.
                            year ib3.int_month ib1.int_type ib1.int_same

                        * different survey and admin samples
                        reg `var1'_`type' ib1.edu `vars_reg' if sample_emp_`type'==1 &
                            sex==`s'
                        est store `var1'_unrt_`type'`s'

                        * the same survey and admin sample
                        reg `var1'_`type' ib1.edu `vars_reg' if `var1'_a!=. & `var1'_s!=.
                             & sample_emp_a==1 & sample_emp_s==1 & sex==`s'
                        est store `var1'_rt_`type'`s'

                        reg `var1'_interm_`type' ib1.edu `vars_reg' if `var1'_a!=. & `
                            var1'_s!=. & sample_emp_a==1 & sample_emp_s==1 & sex==`s'
                        est store `var1'_rit_`type'`s'

                }
```

```
        }
}
* different survey and admin samples
suest lnearnspr_unrt_s0 lnearnspr_unrt_a0, vce(cluster pid) // men
test ([lnearnspr_unrt_s0_mean]2.edu = [lnearnspr_unrt_a0_mean]2.edu) ///
        ([lnearnspr_unrt_s0_mean]3.edu = [lnearnspr_unrt_a0_mean]3.edu)

suest lnearnspr_unrt_s1 lnearnspr_unrt_a1, vce(cluster pid) // women
test ([lnearnspr_unrt_s1_mean]2.edu = [lnearnspr_unrt_a1_mean]2.edu ) ///
        ([lnearnspr_unrt_s1_mean]3.edu = [lnearnspr_unrt_a1_mean]3.edu)

* the same survey and admin sample
suest lnearnspr_rt_s0 lnearnspr_rt_a0, vce(cluster pid) // men
test ([lnearnspr_rt_s0_mean]2.edu = [lnearnspr_rt_a0_mean]2.edu) ///
        ([lnearnspr_rt_s0_mean]3.edu = [lnearnspr_rt_a0_mean]3.edu)

suest lnearnspr_rt_s1 lnearnspr_rt_a1, vce(cluster pid) // women
test ([lnearnspr_rt_s1_mean]2.edu = [lnearnspr_rt_a1_mean]2.edu) ///
        ([lnearnspr_rt_s1_mean]3.edu = [lnearnspr_rt_a1_mean]3.edu)

* - compare survey with intermediate measure
suest lnearnspr_rt_s0 lnearnspr_rit_a0, vce(cluster pid) // men
test ([lnearnspr_rt_s0_mean]2.edu = [lnearnspr_rit_a0_mean]2.edu) ///
        ([lnearnspr_rt_s0_mean]3.edu = [lnearnspr_rit_a0_mean]3.edu)

suest lnearnspr_rt_s1 lnearnspr_rit_a1, vce(cluster pid) // women
test ([lnearnspr_rt_s1_mean]2.edu = [lnearnspr_rit_a1_mean]2.edu) ///
        ([lnearnspr_rt_s1_mean]3.edu = [lnearnspr_rit_a1_mean]3.edu)

* - compare intermediate with admin measure
suest lnearnspr_rit_a0 lnearnspr_rt_a0, vce(cluster pid) // men
test ([lnearnspr_rit_a0_mean]2.edu = [lnearnspr_rt_a0_mean]2.edu) ///
        ([lnearnspr_rit_a0_mean]3.edu = [lnearnspr_rt_a0_mean]3.edu)

suest lnearnspr_rit_a1 lnearnspr_rt_a1, vce(cluster pid) // women
test ([lnearnspr_rit_a1_mean]2.edu = [lnearnspr_rt_a1_mean]2.edu) ///
        ([lnearnspr_rit_a1_mean]3.edu = [lnearnspr_rt_a1_mean]3.edu)

**************************************************************************
* output reg tables
**************************************************************************
qui do "${do}\tables_labels.do"

foreach sample in unr {
        foreach var in lnearnspr {
                estout `var'_`sample'_edu_s0 `var'_`sample'_edu_a0 `var'_`sample'_edu_s1
                    `var'_`sample'_edu_a1 ///
                        using "${tabls}\reg_`sample'_`var'_edu.tex" , cells(b(star fmt
                            (%9.3f)) (se(par fmt(%9.3f)) p(fmt(%9.3f)))) stats(r2_a N,
                            fmt(%9.3f %9.0g) labels(R-squared Observations)) stardrop(*)
                            style(tex) abbrev mlabels(none) label nolz varwidth(60)
                            modelwidth(16) collabels(none) noomitted drop(${labels_drop`
                            var'_edu}) varlabels(${labels_`var'},) refcat(${labels_ref`
                            var'}, label(ref)) order(${labels_order`var'_edu}) indicate(\
                            hline Controls=${labels_controls`var'_edu}) replace
        }
}

foreach sample in r {
        foreach var in lnearnspr {
                estout `var'_`sample'_edu_s0 `var'_`sample'interm_edu_a0 `var'_`sample'
                    _edu_a0 `var'_`sample'_edu_s1 `var'_`sample'interm_edu_a1 `var'_`
                    sample'_edu_a1 ///
                        using "${tabls}\reg_`sample'_`var'_edu.tex" , cells(b(star fmt
                            (%9.3f)) (se(par fmt(%9.3f)) p(fmt(%9.3f)))) stats(r2_a N,
                            fmt(%9.3f %9.0g) labels(R-squared Observations)) stardrop(*)
                            style(tex) abbrev mlabels(none) label nolz varwidth(60)
                            modelwidth(16) collabels(none) noomitted drop(${labels_drop`
                            var'_edu}) varlabels(${labels_`var'},) refcat(${labels_ref`
                            var'}, label(ref)) order(${labels_order`var'_edu}) indicate(\
                            hline Controls=${labels_controls`var'_edu}) replace
        }
}
```

```
log close
```

## application_bias_returns_training.do

```
set more off
est clear
cap log close
log using "${log}\log_application_bias_returns_training.log", replace
*****************************************************
* Table 12: Returns to job training: log-earnings regression
* Table 13: Returns to job training: being an earner regression
* Table A.7: Returns to job training: log-earnings regression (restricted to the same
    sample in the survey and administrative data)
* Table A.8: Returns to job training: being an earner regression (restricted to the same
    sample in the survey and administrative data)
*****************************************************
set more off
use "${input}\data_analysis.dta", clear
keep if waves >= 2
keep if age>18 & age<65

* identify who is unemployed
foreach type in s a {
        gen unempl_`type' =  0

        * on unemployment of any kind (including the job training one, but also just
            missed a year)
        replace unempl_`type' = 1 if act_unemp_months>0 // unemployed for some period
        replace unempl_`type' = 1 if ui_rec`type' == 1 // receiving UI for any duration
        replace unempl_`type' = 1 if ua_rec`type' == 1 // receiving UA
        replace unempl_`type' = 1 if aclc_rec`type' == 1 // receiving ACLC

        replace unempl_`type' = 0 if act6 >0 | act7 >0 | act8 >0 | act9 > 0 | act10 > 0 |
             act11 > 0 // exclude if inactive
}

* average annual earnings in 2007 = 27,458; 2008 = 28,255; 2009 = 28,537; 2010 = 28,715;
    note that calendar year=survey year - 1; Source: Statistik Austria, http://www.
    statistik.at/web_de/statistiken/preise/verbraucherpreisindex_vpi_hvpi/index.html
foreach var in earnspr_g_s earnspr_g_a  { // adjust 2008-2010 earnings to 2011 earnings
    by growth in average earnings
        replace `var' = `var'*104.5779/100 if year==2008
        replace `var' = `var'*101.628/100 if year==2009
        replace `var' = `var'*100.6238/100 if year==2010
}
cap drop lnearnspr*

xtset pid folge // tell stata these are panel data
foreach type in s a {
        gen lnearnspr_`type' = ln(earnspr_g_`type') // log of earnings in t
        gen f1_earnspr_`type' = f1.earnspr_g_`type' // earnings in t+1
        gen f1_lnearnspr_`type' = ln(f1_earnspr_`type') // log of earnings in t+1
        gen f1_earner_`type' = f1.earner_`type' // earner in t+1
}

* define sample for regression
foreach type in s a { // survey vs admin
        gen sample_unemp_`type' = [unempl_`type'==1 & ui_rec`type' != -1 & ua_rec`type'
            != -1 & aclc_rec`type' != -1] // unemployed and with non-missing/non-imputed
            benefits receipt in t
}

* identify for each individual last observed year of being in sample for regression, i.e.
     being unemployed with lead positive earnings or non-mising earner status
foreach type in s a {
        by pid: egen lasttime_lnearnspr_`type' = max(cond([f1_lnearnspr_`type' != . &
            sample_unemp_`type' == 1], folge, .))
        replace lasttime_lnearnspr_`type' = . if lasttime_lnearnspr_`type'!=folge

        by pid: egen lasttime_earner_`type' = max(cond([f1_earner_`type' != . &
            sample_unemp_`type' == 1], folge, .))
        replace lasttime_earner_`type' = . if lasttime_earner_`type'!=folge
}
```

```
* set to missing lead earnings/ earner status if not last observed year of being in
    sample
foreach type in s a {
        replace f1_lnearnspr_`type' = . if lasttime_lnearnspr_`type'==.
        replace f1_earner_`type' = . if lasttime_earner_`type'==.
}

* reg earnings/earner in t+1 on training and earnings in t
set more off
foreach type in s a { // survey vs admin
        foreach var1 in lnearnspr earner { // log of earnings
                cap drop temp_*
                gen temp_earnspr = earnspr_g_`type' / 1000 // job-training-year earnings
                    in thousand

                local vars_reg temp_earnspr ib1.edu ib8.age_gr ib1.region ib0.civil_serv
                    ib1.country_birth ib0.proxy_partner ib1.folge ib3.int_month ib1.
                    int_type ib1.int_same

                * different survey and admin samples
                reg f1_`var1'_`type' ib0.paid_jobcourse `vars_reg' if sample_unemp_`type
                    '==1 & paid_jobcourse!=5 & paid_jobcourse!=6
                est store f1`var1'_unr_`type'

                * restrict to the same survey and admin sample
                reg f1_`var1'_`type' ib0.paid_jobcourse `vars_reg' if sample_unemp_a==1 &
                     sample_unemp_s == 1 & f1_`var1'_a!=. & f1_`var1'_s!=. & earnspr_g_a
                    !=. & earnspr_g_s!=. & paid_jobcourse!=5 & paid_jobcourse!=6
                est store f1`var1'_r_`type'
        }
}

*** Wald test if returns to training based on two datasets are equivalent
gen temp_earnspr_s = earnspr_g_s / 1000 // survey job-training-year earnings in thousand
gen temp_earnspr_a = earnspr_g_a / 1000 // admin job-training-year earnings in thousand
qui foreach type in s a { // survey vs admin
        foreach var1 in lnearnspr earner { // log of earnings
                local vars_reg temp_earnspr_`type' ib1.edu ib8.age_gr ib1.region ib0.
                    civil_serv ib1.country_birth ib0.proxy_partner ib1.folge ib3.
                    int_month ib1.int_type ib1.int_same

                * different survey and admin samples
                reg f1_`var1'_`type' ib0.paid_jobcourse `vars_reg' if sample_unemp_`type
                    '==1 & paid_jobcourse!=5 & paid_jobcourse!=6
                est store f1`var1'_unrt_`type'

                * restrict to the same survey and admin sample
                reg f1_`var1'_`type' ib0.paid_jobcourse `vars_reg' if sample_unemp_a==1 &
                     sample_unemp_s == 1 & f1_`var1'_a!=. & f1_`var1'_s!=. & earnspr_g_a
                    !=. & earnspr_g_s!=. & paid_jobcourse!=5 & paid_jobcourse!=6
                est store f1`var1'_rt_`type'
        }
}

** test jointly for equivalence in the returns to job training
* different survey and admin samples
suest f1lnearnspr_unrt_s f1lnearnspr_unrt_a
test ([f1lnearnspr_unrt_s_mean]1.paid_jobcourse = [f1lnearnspr_unrt_a_mean]1.
    paid_jobcourse) ///
        ([f1lnearnspr_unrt_s_mean]2.paid_jobcourse = [f1lnearnspr_unrt_a_mean]2.
            paid_jobcourse) ///
        ([f1lnearnspr_unrt_s_mean]3.paid_jobcourse = [f1lnearnspr_unrt_a_mean]3.
            paid_jobcourse) ///
        ([f1lnearnspr_unrt_s_mean]4.paid_jobcourse = [f1lnearnspr_unrt_a_mean]4.
            paid_jobcourse)

suest f1earner_unrt_s f1earner_unrt_a
test ([f1earner_unrt_s_mean]1.paid_jobcourse = [f1earner_unrt_a_mean]1.paid_jobcourse)
    ///
        ([f1earner_unrt_s_mean]2.paid_jobcourse = [f1earner_unrt_a_mean]2.paid_jobcourse)
            ///
        ([f1earner_unrt_s_mean]3.paid_jobcourse = [f1earner_unrt_a_mean]3.paid_jobcourse)
```

```
            ///
        ([f1earner_unrt_s_mean]4.paid_jobcourse = [f1earner_unrt_a_mean]4.paid_jobcourse)

* the same survey and admin sample
suest f1lnearnspr_rt_s f1lnearnspr_rt_a
test ([f1lnearnspr_rt_s_mean]1.paid_jobcourse = [f1lnearnspr_rt_a_mean]1.paid_jobcourse)
    ///
        ([f1lnearnspr_rt_s_mean]2.paid_jobcourse = [f1lnearnspr_rt_a_mean]2.
            paid_jobcourse) ///
        ([f1lnearnspr_rt_s_mean]3.paid_jobcourse = [f1lnearnspr_rt_a_mean]3.
            paid_jobcourse) ///
        ([f1lnearnspr_rt_s_mean]4.paid_jobcourse = [f1lnearnspr_rt_a_mean]4.
            paid_jobcourse)

suest f1earner_rt_s f1earner_rt_a
test ([f1earner_rt_s_mean]1.paid_jobcourse = [f1earner_rt_a_mean]1.paid_jobcourse) ///
        ([f1earner_rt_s_mean]2.paid_jobcourse = [f1earner_rt_a_mean]2.paid_jobcourse) ///
        ([f1earner_rt_s_mean]3.paid_jobcourse = [f1earner_rt_a_mean]3.paid_jobcourse) ///
        ([f1earner_rt_s_mean]4.paid_jobcourse = [f1earner_rt_a_mean]4.paid_jobcourse)

**********************************************************************
* output reg tables
**********************************************************************
qui do "${do}\tables_labels.do"

foreach var in lnearnspr earner {
        estout f1`var'_unr_s f1`var'_unr_a ///
                using "${tabls}\reg_unr_`var'_jobtrain.tex" , cells(b(star fmt(%9.3f)) (
                    se(par fmt(%9.3f)) p(fmt(%9.3f)))) stats(r2_a N, fmt(%9.3f %9.0g)
                    labels(R-squared Observations)) stardrop(*) style(tex) abbrev mlabels
                    (none) label nolz varwidth(60) modelwidth(16) collabels(none)
                    noomitted drop(${labels_drop`var'_jtr})             varlabels(${
                    labels_`var'},) refcat(${labels_ref`var'}, label(ref)) order(${
                    labels_order`var'_jtr}) indicate(\hline Controls=${labels_controls`
                    var'_jtr}) replace

        estout f1`var'_r_s f1`var'_r_a ///
                using "${tabls}\reg_r_`var'_jobtrain.tex", cells(b(star fmt(%9.3f)) (se(
                    par fmt(%9.3f)) p(fmt(%9.3f)))) stats(r2_a N, fmt(%9.3f %9.0g) labels
                    (R-squared Observations)) stardrop(*) style(tex) abbrev mlabels(none)
                     label nolz varwidth(60) modelwidth(16) collabels(none) noomitted
                    drop(${labels_drop`var'_jtr}) varlabels(${labels_`var'},) refcat(${
                    labels_ref`var'}, label(ref)) order(${labels_order`var'_jtr})
                    indicate(\hline Controls=${labels_controls`var'_jtr}) replace
}
log close
```

### logit_report_receipt.do

```
set more off
est clear
cap log close
log using "${log}\log_logit_report_receipt.log", replace
******************************************************************************************

* Table A.2: Probability of reporting the unemployment insurance (UI) benefit in the
    survey, conditional on receiving it: marginal effects from a logit model
* Table A.4: Probability of reporting the unemployment assistance (UA) in the survey,
    conditional on receiving it: marginal effects from a logit model
******************************************************************************************

use "${input}\data_analysis.dta", clear
keep if age>=16

* UI - unemployment insurance
foreach var in ui {
        foreach var2 in ua {
                foreach var3 in aclc {
                        logit `var'_reportrec ln`var'_a tearnspr_g_a tdearnspr ib3.
                            earnspr_false ib3.`var2'_false ib3.`var3'_false if `var'
                            _reportrec>-1, vce(cluster pid)
                        margins, dydx(*) post
                        estimates store `var'nc_reportrec
```

```
                        local vars_reg sex ib8.age_gr ib1.nad ib0.nch ib1.edu ib1.region
                            ib10.occup ib3.industry ib0.proxy_partner incouple ib1.folge
                            ib1.folge#ib2008.year ib0.civil_serv ib3.int_month `var'
                            _months_a ib1.int_type ib1.int_same ib1.country_birth ib5.
                            health ib0.paid_jobcourse
                        logit `var'_reportrec ln`var'_a tearnspr_g_a tdearnspr ib3.
                            earnspr_false ib3.`var2'_false ib3.`var3'_false `vars_reg' if
                             `var'_reportrec>-1, vce(cluster pid)
                        margins, dydx(*) post
                        estimates store `var'_reportrec
                }
        }
}
* UA - unemployment assistance
foreach var in ua {
        foreach var2 in ui {
                foreach var3 in aclc {
                        logit `var'_reportrec ln`var'_a tearnspr_g_a tdearnspr ib3.
                            earnspr_false ib3.`var2'_false ib3.`var3'_false if `var'
                            _reportrec>-1, vce(cluster pid)
                        margins, dydx(*) post
                        estimates store `var'nc_reportrec

                        local vars_reg sex ib8.age_gr ib1.nad ib0.nch ib1.edu ib1.region
                            ib10.occup ib3.industry ib0.proxy_partner incouple ib1.folge
                            ib1.folge#ib2008.year ib0.civil_serv ib3.int_month `var'
                            _months_a ib1.int_type ib1.int_same ib1.country_birth ib5.
                            health ib0.paid_jobcourse
                        logit `var'_reportrec ln`var'_a tearnspr_g_a tdearnspr ib3.
                            earnspr_false ib3.`var2'_false ib3.`var3'_false `vars_reg' if
                             `var'_reportrec>-1, vce(cluster pid)
                        margins, dydx(*) post
                        estimates store `var'_reportrec
                }
        }
}
*************************************************************************
* output reg tables
*************************************************************************
qui do "${do}\tables_labels.do"
*************************************************************************
* tables for online supplementary materials
*************************************************************************
foreach var in ui ua {
        estout `var'nc_reportrec `var'_reportrec using "${tabls}\logit_`var'_receipt.tex
            ", cells(b(star fmt(%9.3f)) (se(par fmt(%9.3f)) p(fmt(%9.3f)))) stats(N, fmt
            (%9.0g) labels(Observations)) stardrop(*) style(tex) abbrev wrap mlabels(none
            ) label nolz varwidth(60) modelwidth(16) collabels(none) noomitted drop(${
            labels_log_shdrop`var'}) varlabels(${labels_`var'},) refcat(${labels_ref`var
            '}, label(ref)) order(${labels_log_order`var'}) indicate(\hline Controls=${
            labels_log_controls`var'}) replace
}

log close
```

## tables_labels.do

```
/*****************************************
* This do-file defines locals and globals that are used for labels in the regression
    tables:
* labels of variables, reference variable values, order of coefficients, what
    coefficients to be dropped, indicator (yes/no) for inclusion of control variables.
***************************************/
* variable labels
local cons _cons Constant
local dearnspr dearnspr "Survey--admin earnings"
local tdearnspr tdearnspr "Survey--admin earnings (in thousand)"
local tearnspr_g_a tearnspr_g_a "Admin earnings (in thousand)"
local error_uben error_uben "Admin--survey benefits"
local lnui_a lnui_a "Ln admin UI"
local lnua_a lnua_a "Ln admin UA"
local lnaclc_a lnaclc_a "Ln admin ACLC"
```

```
local lnui_s lnui_s "Ln survey UI"
local lnua_s lnua_s "Ln survey UA"
local lnaclc_s lnaclc_s "Ln survey ACLC"
local earnspr_cat 1.earnspr_false "\hspace{0.1cm} false +" 2.earnspr_false "\hspace{0.1cm
    } false —" 3.earnspr_false "\hspace{0.1cm} true —" 4.earnspr_false "\hspace{0.1cm}
    true +"
local uben_cat 1.uben_false#c.error_uben "\hspace{0.1cm} false +" 2.uben_false#c.
    error_uben "\hspace{0.1cm} false —" 3.uben_false#c.error_uben "\hspace{0.1cm} true
    —" 4.uben_false#c.error_uben "\hspace{0.1cm} true + \& survey underreport" 5.
    uben_false#c.error_uben "\hspace{0.1cm} true + \& survey overreport" 6.uben_false#c.
    error_uben "\hspace{0.1cm} missing/imputed"
local ui_cat 1.ui_false "\hspace{0.1cm} false +" 2.ui_false "\hspace{0.1cm} false —" 3.
    ui_false "\hspace{0.1cm} true —" 4.ui_false "\hspace{0.1cm} true +" 5.ui_false "\
    hspace{0.1cm} missing/imputed"
local ua_cat 1.ua_false "\hspace{0.1cm} false +" 2.ua_false "\hspace{0.1cm} false —" 3.
    ua_false "\hspace{0.1cm} true —" 4.ua_false "\hspace{0.1cm} true +" 5.ua_false "\
    hspace{0.1cm} missing/imputed"
local aclc_cat 1.aclc_false "\hspace{0.1cm} false +" 2.aclc_false "\hspace{0.1cm} false
    —" 3.aclc_false "\hspace{0.1cm} true —" 4.aclc_false "\hspace{0.1cm} true +" 5.
    aclc_false "\hspace{0.1cm} missing/imputed"
local sex sex "Woman"
local incouple incouple "In a couple"
local age 1.age_gr "\hspace{0.1cm} 0-4" 2.age_gr "\hspace{0.1cm} 5-9" 3.age_gr "\hspace
    {0.1cm} 10-14" 4.age_gr "\hspace{0.1cm} 16-19" 5.age_gr "\hspace{0.1cm} 20-24" 6.
    age_gr "\hspace{0.1cm} 25-29"  7.age_gr "\hspace{0.1cm} 30-34" 8.age_gr "\hspace{0.1
    cm} 35-39" 9.age_gr "\hspace{0.1cm} 40-44" 10.age_gr "\hspace{0.1cm} 45-49" 11.age_gr
     "\hspace{0.1cm} 50-54" 12.age_gr "\hspace{0.1cm} 55-59" 13.age_gr "\hspace{0.1cm}
    60-64" 14.age_gr "\hspace{0.1cm} 65-69" 15.age_gr "\hspace{0.1cm} 70-74" 16.age_gr "\
    hspace{0.1cm} 75+"
local nch 1.nch "\hspace{0.1cm} 1 child" 2.nch "\hspace{0.1cm} 2 children" 3.nch "\hspace
    {0.1cm} 3+ children"
local nad 1.nad "\hspace{0.1cm} 1 adult" 2.nad "\hspace{0.1cm} 2 adults" 3.nad "\hspace
    {0.1cm} 3+ adults"
local edu 1.edu "\hspace{0.1cm} Lower middle or less" 2.edu "\hspace{0.1cm} middle" 3.edu
     "\hspace{0.1cm} high"
local wave 2.folge "\hspace{0.1cm} wave 2" 3.folge "\hspace{0.1cm} wave 3" 4.folge "\
    hspace{0.1cm} wave 4"
local occup 1.occup "\hspace{0.1cm} armed forces" 2.occup "\hspace{0.1cm} senior
    officials and managers" 3.occup "\hspace{0.1cm} professionals" 4.occup  "\hspace{0.1
    cm} associate prof. and technical" 5.occup "\hspace{0.1cm} clerks (admin and
    secretarial)" 6.occup "\hspace{0.1cm} service and sales workers" 7.occup "\hspace{0.1
    cm} skilled agricultural" 8.occup "\hspace{0.1cm} craft and trades workers" 9.occup
    "\hspace{0.1cm} plant and machine operators" 10.occup "\hspace{0.1cm} elementary
    occupations" 11.occup "\hspace{0.1cm} n/a"  12.occup "\hspace{0.1cm} missing"
local industry 1.industry "\hspace{0.1cm} agriculture" 2.industry "\hspace{0.1cm} mining
    and quarrying" 3.industry "\hspace{0.1cm} manufacturing" 4.industry  "\hspace{0.1cm}
    electricity, gas etc" 5.industry "\hspace{0.1cm} water supply, sewage etc" 6.industry
     "\hspace{0.1cm} construction" 7.industry "\hspace{0.1cm} trade" 8.industry "\hspace
    {0.1cm} transportation" 9.industry "\hspace{0.1cm} accommodation and food" 10.
    industry "\hspace{0.1cm} info and communication" 11.industry "\hspace{0.1cm} finance"
     12.industry "\hspace{0.1cm} real estate" 13.industry "\hspace{0.1cm} science" 14.
    industry "\hspace{0.1cm} other professional etc" 15.industry  "\hspace{0.1cm} admin
    and support services" 16.industry "\hspace{0.1cm} public admin., defence etc" 17.
    industry "\hspace{0.1cm} education" 18.industry "\hspace{0.1cm} health services" 19.
    industry "\hspace{0.1cm} residential care and social work" 20.industry "\hspace{0.1cm
    } arts, entertainment etc" 21.industry "\hspace{0.1cm} other services" 22.industry "\
    hspace{0.1cm} activities of hhs as employers"   23.industry "\hspace{0.1cm}
    activities of extra-terr. organis."  24.industry "\hspace{0.1cm} missing" 25.industry
     "\hspace{0.1cm} n/a"
local region 1.region "Region: Vienna" 2.region "\hspace{0.1cm} 100,000+ residents" 3.
    region "\hspace{0.1cm} 10,000-100,000 residents" 4.region "\hspace{0.1cm} less than
    10,000 residents" 5.region "\hspace{0.1cm} missing"
local proxy proxy_always "Always proxy interview" 0.proxy_partner "\hspace{0.1cm} no
    proxy" 1.proxy_partner "\hspace{0.1cm} partner is proxy" 2.proxy_partner "\hspace{0.1
    cm} someone else is proxy"
local civil_serv 0.civil_serv "\hspace{0.1cm} not civil servant" 1.civil_serv "\hspace
    {0.1cm} yes" 2.civil_serv "\hspace{0.1cm} missing"
local int_month 4.int_month "\hspace{0.1cm} Apr" 5.int_month "\hspace{0.1cm} May"  6.
    int_month "\hspace{0.1cm} Jun"  7.int_month "\hspace{0.1cm} Jul" 8.int_month "\hspace
    {0.1cm} Aug"  9.int_month "\hspace{0.1cm} Sep"  10.int_month "\hspace{0.1cm} Oct" 11.
    int_month "\hspace{0.1cm} Nov"
local int_type 1.int_type "\hspace{0.1cm} interview in person" 2.int_type "\hspace{0.1cm}
     interview by phone"
```

```
local int_same 1.int_same "\hspace{0.1cm} same interviewer: yes" 2.int_same "\hspace{0.1
    cm} no"  3.int_same "\hspace{0.1cm} missing" 4.int_same "\hspace{0.1cm} n/a"  5.
    int_same "\hspace{0.1cm} don't know"
local country 1.country_birth "\hspace{0.1cm} Austria" 2.country_birth "\hspace{0.1cm}
    EU15/EFTA" 3.country_birth "\hspace{0.1cm} new EU12" 4.country_birth "\hspace{0.1cm}
    former Yugosl."  5.country_birth "\hspace{0.1cm} Turkey" 6.country_birth "\hspace{0.1
    cm} other" 7.country_birth "\hspace{0.1cm} missing"
local health 1.health "\hspace{0.1cm} very good" 2.health "\hspace{0.1cm} good"  3.health
     "\hspace{0.1cm} fair" 4.health "\hspace{0.1cm} bad"  5.health "\hspace{0.1cm} very
    bad"  6.health "\hspace{0.1cm} missing"
local paid_jobcourse 1.paid_jobcourse "\hspace{0.1cm} Mostly paid with own resources" 2.
    paid_jobcourse "\hspace{0.1cm} Employer" 3.paid_jobcourse "\hspace{0.1cm} Labour
    market agency" 4.paid_jobcourse "\hspace{0.1cm} Other institutions" 5.paid_jobcourse
    "\hspace{0.1cm} missing" 6.paid_jobcourse "\hspace{0.1cm} n/a" 7.paid_jobcourse "\
    hspace{0.1cm} Don't know"
local paid_jobcourse_tr 1.paid_jobcourse "\hspace{0.1cm} Mostly paid with own resources"
    2.paid_jobcourse "\hspace{0.1cm} Employer" 3.paid_jobcourse "\hspace{0.1cm} \textbf{
    Labour market agency}" 4.paid_jobcourse "\hspace{0.1cm} Other institutions" 5.
    paid_jobcourse "\hspace{0.1cm} missing" 6.paid_jobcourse "\hspace{0.1cm} n/a" 7.
    paid_jobcourse "\hspace{0.1cm} Don't know"
foreach var in ui ua aclc {
        foreach type in s a {
                local `var'_rec`type' 1.`var'_rec`type' "\hspace{0.1cm} yes" 2.`var'_rec`
                    type' "\hspace{0.1cm} missing/imputed"
        }
}
foreach var in earnspr {
        foreach d in a {
                local `var'_months_`d' 0.`var'_months_`d' "\hspace{0.1cm} 0" 1.`var'
                    _months_`d' "\hspace{0.1cm} 1" 2.`var'_months_`d' "\hspace{0.1cm} 2"
                    3.`var'_months_`d' "\hspace{0.1cm} 3"         4.`var'_months_`d' "\
                    hspace{0.1cm} 4" 5.`var'_months_`d' "\hspace{0.1cm} 5"  6.`var'
                    _months_`d' "\hspace{0.1cm} 6"  7.`var'_months_`d' "\hspace{0.1cm} 7"
                     8.`var'_months_`d' "\hspace{0.1cm} 8"  9.`var'_months_`d' "\hspace
                    {0.1cm} 9"  10.`var'_months_`d' "\hspace{0.1cm} 10" 11.`var'_months_`
                    d' "\hspace{0.1cm} 11" 12.`var'_months_`d' "\hspace{0.1cm} 12"
        }
}
foreach var in ui ua aclc {
        local `var'_months_a `var'_months_a "Admin benefit duration (in months)"
}
local temp_earnspr temp_earnspr "Job-training-year earnings (in thousand)"

* labels for reference values
qui {
        local ref_earnspr_f 1.earnspr_false "Earnings: true --"
        local ref_earnspr_fs 2.earnspr_false "Earnings: true --"
        local ref_ua_f 1.ua_false "UA: true --"
        local ref_ua_fs 2.ua_false "UA: true --"
        local ref_ui_f 1.ui_false "UI: true --"
        local ref_aclc_f 1.aclc_false "ACLC: true --"
        local ref_age 4.age_gr "Age: 35-39"
        local ref_nch 1.nch "No children in the hh"
        local ref_nad 2.nad "1 adult in the hh"
        local ref_region 2.region "Region: Vienna"
        local ref_edu 2.edu "Education: low"
        local ref_occup 2.occup "Occupation: elementary"
        local ref_occup_ui 2.occup "Occupation: elementary"
        local ref_occup_ua 2.occup "Occupation: elementary"
        local ref_industry 1.industry "Industry: manufacturing"
        local ref_industry_ui 1.industry "Industry: manufacturing"
        local ref_industry_ua 6.industry "Industry: manufacturing"
        local ref_wave_year 1.folge#2009.year "Wave 1 $\times$ year 2008"
        local ref_wave 2.folge "Wave 1"
        local ref_year 2009.year "Survey year: 2008"
        local ref_proxy 1.proxy_partner "No proxy"
        local ref_civil_serv 1.civil_serv "Civil servant: no"
        local ref_civil_serv_ui 2.civil_serv "Civil servant: no"
        local ref_civil_serv_ua 2.civil_serv "Civil servant: no"
        local ref_int_month 4.int_month "Month of interview: Mar"
        local ref_int_type 2.int_type "Interview in person"
        local ref_int_same 2.int_same "Same interviewer: yes"
        local ref_country 2.country_birth "Country of birth: Austria"
```

```
        local ref_country_ua 3.country_birth "Country of birth: Austria"
        local ref_health 1.health "Health: very bad"
        local ref_paid_jobcourse 3.paid_jobcourse "Job training: did not take"
        foreach var in ui ua aclc {
                local ref_`var'_months_a 2.`var'_months_a "Benefit duration (in months):
                    1"
        }
        local ref_uben_f 2.uben_false#c.error_uben "Admin——survey benefits: true ——"
}
*****************************************************************************
* globals for variables labels, reference values, list of variables to be dropped,
    control variables
* these differ by table
*****************************************************************************
* Tables 4, S1, S2, S5 – Probability of reporting the unemployment insurance (UI) in the
    survey, conditional on receiving it
foreach var in ui {
        * OLS
        global labels_order`var' _cons *sex* ln* *earnspr_g_a *dearnspr *_g_a *_f* *
            months* 3*jobcourse *jobcourse *proxy* *age_gr* *edu* *country* *incouple* *
            nch* *nad* *region* *civil* *occup* *industry* *health* *int*month* *int*type
            * *int*same* *folge* // order of coefficients

        global labels_order`var's _cons ln* *earnspr_g_a *dearnspr *_g_a *_f* *months*
            3*jobcourse *jobcourse *proxy* *age_gr* *edu* *country* *incouple* *nch* *nad
            * *region* *civil* *occup* *industry* *health* *int*type* *int*month* *int*
            same* *folge* // order of coefficients for table by gender

        global labels_controls`var' *age* *incouple* *nad* *nch* *int* *country* *edu* *
            civil* *region* *occup* *industry* *health* *folge* // indicator for
            inclusion of controls

        global labels_shdrop`var' 3.earnspr_false 3.ua_false 5.ua_false 3.aclc_false 2.
            aclc_false 4.aclc_false 5.aclc_false 0.proxy_partner 0*jobcourse 5*jobcourse
            6*jobcourse // what coefficients to drop from short table

        global labels_shdrop`var's 3.earnspr_false 1.ua_false 3.ua_false 5.ua_false 3.
            aclc_false 2.aclc_false 4.aclc_false 5.aclc_false 0.proxy_partner 0*jobcourse
             4*jobcourse 5*jobcourse 6*jobcourse // what coefficients to drop from short
            table by gender

        global labels_drop`var' 3.earnspr_false 3.ua_false 5.ua_false 3.aclc_false 2.
            aclc_false 4.aclc_false 5.aclc_false 8.age_gr 14.age_gr 0.nch 1.nad 1.region
            5.region 1.edu 10.occup 1.occup 2.industry 3.industry 4.industry 5.industry
            11.industry 13.industry 22.industry 24.industry 1.folge 0.proxy_partner 0.
            civil_serv 1.civil_serv 3.int_month 11.int_month 1.int_type 1.int_same 3.
            int_same 5.int_same 1.country_birth 7.country_birth 5.health 6.health 1.folge
             *2008.year 0*jobcourse 5*jobcourse 6*jobcourse // what coefficients to drop
            from long table

        * logit
        global labels_log_order`var' *sex* ln* *earnspr_g_a *dearnspr *_g_a *_f* *months*
              3*jobcourse *jobcourse *proxy* *age_gr* *edu* *country* *incouple* *nch* *
            nad* *region* *civil* *occup* *industry* *health* *int*month* *int*type* *int
            *same* *folge* *year* // order of coefficients

        global labels_log_shdrop`var' 3.earnspr_false 3.ua_false 3.aclc_false 2.
            aclc_false 5.aclc_false         0.proxy_partner 0*jobcourse 5*jobcourse 6*
            jobcourse // what coefficients to drop from short table

        global labels_log_controls`var' *age* *incouple* *nad* *nch* *int* *country* *edu
            * *civil* *region* *occup* *industry* *health* *folge* *year* // indicator
            for inclusion of controls

        * variable labels
        global labels_`var' `cons' `dearnspr' `earnspr_g_a' `tdearnspr' `tearnspr_g_a' `
            lnui_a' `lnua_a' `lnaclc_a' `earnspr_cat' `ui_cat' `ua_cat' `aclc_cat' `sex'
            `incouple' `age' `nch' `nad' `edu' `wave' `occup' `industry' `region' `proxy'
             `civil_serv' `int_month' ``var'_months_a' `int_type' `int_same' `country' `
            health' `paid_jobcourse' `year'

        * labels for reference values
        global labels_ref`var' `ref_earnspr_f' `ref_ua_f' `ref_ui_f' `ref_aclc_f' `
```

```
            ref_age ' `ref_nch ' `ref_nad ' `ref_region ' `ref_edu ' `ref_occup_ui ' `
            ref_industry_ui ' `ref_wave ' `ref_wave_year ' `ref_proxy ' `ref_civil_serv_ui ' `
            ref_int_month ' `ref_`var'_months_a ' `ref_int_type ' `ref_int_same ' `
            ref_country ' `ref_health ' `ref_paid_jobcourse ' `ref_year '

        global labels_ref`var's `ref_earnspr_f ' `ref_ua_fs ' `ref_ui_f ' `ref_aclc_f ' `
            ref_age ' `ref_nch ' `ref_nad ' `ref_region ' `ref_edu ' `ref_occup ' `ref_industry
            ' `ref_wave ' `ref_wave_year ' `ref_proxy ' `ref_civil_serv ' `ref_int_month ' `
            ref_`var'_months_a ' `ref_int_type ' `ref_int_same ' `ref_country ' `ref_health '
            `ref_paid_jobcourse ' `ref_year '
}

* Tables 5, S3, S4, S6 - Probability of reporting the unemployment assistance (UA) in the
     survey, conditional on receiving it
foreach var in ua {
        * OLS
        global labels_order`var' _cons *sex* ln* *earnspr_g_a *dearnspr *_g_a *_f* *
            months*  3*jobcourse *jobcourse *proxy* *age_gr* *edu* *country* *incouple* *
            nch* *nad* *region* *civil* *occup* *industry* *health* *int*month* *int*type
            * *int*same* *folge* // order of coefficients

        global labels_order`var's _cons ln* *earnspr_g_a *dearnspr *_g_a *_f* *months*
            3*jobcourse *jobcourse *proxy* *age_gr* *edu* *country* *incouple* *nch* *
            civil* *nad* *region* *occup* *industry* *health* *int*month* *int*type* *int
            *same* *folge* // order of coefficients for table by gender

        global labels_controls`var' *age* *incouple* *nad* *nch* *int* *country* *edu* *
            civil* *region* *occup* *industry* *health* *folge* // indicator for
            inclusion of controls

        global labels_shdrop`var' 3.earnspr_false 3.ui_false 3.aclc_false 2.aclc_false 4.
            aclc_false 5.aclc_false 0.proxy_partner 0*jobcourse 4*jobcourse 6*jobcourse
            // what coefficients to drop from short table

        global labels_shdrop`var's 3.earnspr_false 3.ui_false 3.aclc_false 2.aclc_false
            4.aclc_false 5.aclc_false 0.proxy_partner 0*jobcourse 2*jobcourse 4*jobcourse
             6*jobcourse // what coefficients to drop from short table by gender

        global labels_drop`var' 3.earnspr_false 3.ui_false 3.aclc_false 2.aclc_false 4.
            aclc_false 5.aclc_false 8.age_gr 14.age_gr 0.nch 1.nad 1.region 1.edu 10.
            occup 1.occup 1.industry 2.industry 3.industry 4.industry 5.industry 11.
            industry 12.industry 13.industry 14.industry 22.industry 24.industry 1.folge
            0.proxy_partner 0.civil_serv 1.civil_serv 3.int_month 10.int_month 11.
            int_month 1.int_type 1.int_same 1.country_birth 2.country_birth 5.health 1.
            folge *2008.year 0*jobcourse 4*jobcourse 6*jobcourse // what coefficients to
            drop from long table

        * logit
        global labels_log_order`var' *sex* ln* *earnspr_g_a *dearnspr *_g_a *_f* *months*
              3*jobcourse *jobcourse *proxy* *age_gr* *edu* *country* *incouple* *nch* *
            nad* *region* *civil* *occup* *industry* *health* *int*month* *int*type* *int
            *same* *folge* *year* // order of coefficients

        global labels_log_controls`var' *age* *incouple* *nad* *nch* *int* *country* *edu
            * *civil* *region* *occup* *industry* *health* *folge* *year* // indicator
            for inclusion of controls

        global labels_log_shdrop`var' 3.earnspr_false 3.ui_false 3.aclc_false 2.
            aclc_false 5.aclc_false         0.proxy_partner 0*jobcourse 4*jobcourse 6*
            jobcourse // what coefficients to drop from short table

        * variable labels
        global labels_`var' `cons' `dearnspr' `earnspr_g_a' `tdearnspr' `tearnspr_g_a' `
            lnui_a' `lnua_a' `lnaclc_a' `earnspr_cat' `ui_cat' `ua_cat' `aclc_cat' `sex'
            `incouple' `age' `nch' `nad' `edu' `wave' `occup' `industry' `region' `proxy'
             `civil_serv ' `int_month ' `'var'_months_a ' `int_type ' `int_same ' `country ' `
            health ' `paid_jobcourse ' `year '

        * labels for reference values
        global labels_ref`var' `ref_earnspr_f ' `ref_ua_f ' `ref_ui_f ' `ref_aclc_f ' `
            ref_age ' `ref_nch ' `ref_nad ' `ref_region ' `ref_edu ' `ref_occup_ua ' `
            ref_industry_ua ' `ref_wave ' `ref_wave_year ' `ref_proxy ' `ref_civil_serv_ua ' `
            ref_int_month ' `ref_`var'_months_a ' `ref_int_type ' `ref_int_same ' `
```

```
            ref_country_ua ' `ref_health ' `ref_paid_jobcourse ' `ref_year '

        global labels_ref `var's `ref_earnspr_f ' `ref_ua_f ' `ref_ui_f ' `ref_aclc_f ' `
            ref_age ' `ref_nch ' `ref_nad ' `ref_region ' `ref_edu ' `ref_occup ' `ref_industry
            ' `ref_wave ' `ref_wave_year ' `ref_proxy ' `ref_civil_serv ' `ref_int_month ' `
            ref_`var'_months_a ' `ref_int_type ' `ref_int_same ' `ref_country ' `ref_health '
            `ref_paid_jobcourse ' `ref_year '
}

* Table 8 — OLS model of the error in earnings on the reverse error in sum of benefits
foreach var in error_earnspr { // earnings
        global labels_order`var' _cons error_uben 2*false* 4*false* 5*false* 1*false* *
            months* *incouple* *age_gr* *edu* *nch* *nad* *health* *civil* *occup* *
            industry* *region* *country* *proxy* *int*type* *int*same* *folge* // order
            of coefficients

        global labels_controls`var' *age* *incouple* *nad* *nch* *int*type* *int*same* *
            country* *edu* *region* *occup* *industry*  *health* *folge* *proxy* *
            int_month* *earnspr_months_* *civil_serv* // indicator for inclusion of
            controls

        global labels_shdrop`var' // what coefficients to drop from short table

        * variable labels
        global labels_`var' `cons' `sex' `incouple' `age' `nch' `nad' `edu' `wave' `occup
            ' `industry' `region' `proxy' `civil_serv ' `int_month' `int_type' `int_same'
            `country' `health'        `paid_jobcourse' `year' `error_uben' `uben_cat' `
            earnspr_months_a'

        * labels for reference values
        global labels_ref`var' `ref_age ' `ref_nch ' `ref_nad ' `ref_region ' `ref_edu ' `
            ref_occup ' `ref_industry ' `ref_wave ' `ref_proxy ' `ref_civil_serv ' `
            ref_int_month ' `ref_int_type ' `ref_int_same ' `ref_country ' `ref_health ' `
            ref_earnspr_months_a ' `ref_uben_f '

}

* Tables 10, 11, 12, 13, S7, S8 — applications for bias in the returns to education and
    to job training
foreach var in lnearnspr earner { // other earnings models
        * application for bias in the returns to education
        global labels_controls`var'_edu *age_gr* *civil* *region* *country* *proxy* *int*
            type* *int*same* *folge* *month* // indicator for inclusion of controls
        global labels_drop`var'_edu 1.edu // what coefficients to drop
        global labels_order`var'_edu *edu* *age_gr* *civil* *region* *country* *proxy* *
            int*type* *int*same* *folge* _cons // order of coefficients

        * application for bias in the returns to job training
        global labels_controls`var'_jtr *edu* *age_gr* *civil* *region* *country* *proxy*
             *int*type* *int*same* *folge* *month* // indicator for inclusion of controls

        global labels_drop`var'_jtr 0.* 4.* // what coefficients to drop
        global labels_order`var'_jtr 3*jobcourse *jobcourse *earnspr* *edu* *age_gr* *
            civil* *region* *country* *folge* _cons // order of coefficients

        * variable labels
        global labels_`var' `cons' `dearnspr' `earnspr_g_a' `lnearnspr_a' `lnearnspr_s' `
            sex' `incouple' `age' `nch' `nad' `edu' `wave' `occup' `industry' `region' `
            proxy' `civil_serv' `int_month' `int_type' `int_same' `country' `health'   `
            paid_jobcourse_tr' `temp_earnspr'

        * labels for reference values
        global labels_ref`var' `ref_age ' `ref_nch ' `ref_nad ' `ref_region ' `ref_edu ' `
            ref_occup ' `ref_industry ' `ref_wave ' `ref_proxy ' `ref_civil_serv ' `
            ref_int_month ' `ref_`var'_months_a ' `ref_int_type ' `ref_int_same ' `
            ref_country ' `ref_health ' `ref_paid_jobcourse '
}
```
